# CDCA3-MYC positive feedback loop promotes bladder cancer progression via ENO1-mediated glycolysis

**DOI:** 10.1186/s13046-025-03325-7

**Published:** 2025-02-20

**Authors:** Dexin Shen, Xiang Yu, Xuefeng Fan, Yu Liang, Dongmei Lu, Zongpan Ke, Lei Wang, Ping Xiang, Jun Xiao

**Affiliations:** 1 Department of Urology, The First Affiliated Hospital of USTC, Division of Life Sciences and Medicine, University of Science and Technology of China, Hefei, 230001 China; 2 Core Facility Center for Medical Sciences, The First Affiliated Hospital of USTC, University of Science and Technology of China, Hefei, 230001 China; 3 Department of Urology, The Second Affiliated Hospital with Nanjing Medical University, Nanjing, China

**Keywords:** Bladder cancer, CDCA3, MYC, TRIM28, Glycolysis

## Abstract

**Background:**

Bladder cancer (BLCA) ranks among the most prevalent malignancies of the urinary system, with its clinical diagnosis predominantly reliant on invasive procedures. Traditional chemotherapy regimens exhibit significant limitations, underscoring the urgency of identifying novel diagnostic biomarkers and strategies to enhance chemotherapy efficacy. CDCA3 has been recognized as a facilitator of BLCA progression, activated by MYBL2. However, its precise regulatory mechanisms in BLCA pathogenesis remain incompletely elucidated.

**Methods:**

To investigate the functional role of CDCA3 in BLCA, MTT and colony formation assays were employed to assess cellular proliferation, while flow cytometry was utilized to evaluate apoptosis and intracellular ROS levels. The expression of CDCA3, ENO1, TRIM28, and MYC was analyzed through WB and qRT-PCR, and Co-IP assays were conducted to delineate interactions among CDCA3, TRIM28, and MYC.

**Results:**

CDCA3, a key regulator of the cell cycle, facilitates BLCA glycolysis by modulating the transcriptional expression of α-Enolase (ENO1), thereby enhancing BLCA progression. Mechanistically, CDCA3 recruits TRIM28, which stabilizes MYC, while MYC transcriptionally upregulates *CDCA3*, establishing a self-reinforcing CDCA3-MYC feedback loop. A risk prediction model incorporating the expression profiles of *CDCA3* and *ENO1* was developed to evaluate the overall survival of patients with BLCA. This model provides a prognostic tool to predict survival outcomes in patients with BLCA based on *CDCA3* and *ENO1* expression levels.

**Conclusions:**

This study delineates a novel role for CDCA3 in the regulation of BLCA glycolysis and identifies its interaction with MYC as a critical positive feedback mechanism, providing fresh insights into the molecular mechanisms underlying BLCA progression.

**Supplementary Information:**

The online version contains supplementary material available at 10.1186/s13046-025-03325-7.

## Introduction

Bladder cancer (BLCA) ranks as the sixth most common malignancy globally [1], with its incidence and mortality rates rising steadily each year, posing a considerable public health challenge. The field currently lacks effective and specific molecular biomarkers for BLCA diagnosis, as existing methods predominantly rely on invasive cystoscopy. This procedure is often painful and poorly tolerated by many patients.

For non-muscle invasive bladder cancer (NMIBC), intravesical chemotherapy combined with transurethral resection of the bladder tumor remains the standard therapeutic approach. However, muscle-invasive bladder cancer (MIBC) exhibits resistance to traditional chemotherapy and is primarily treated through radical cystectomy. NMIBC accounts for approximately 75% of newly diagnosed BLCA cases, characterized by low malignancy, invasion, and metastatic potential. Despite this, NMIBC carries a recurrence rate of 50–70% within five years post-treatment, and 10–15% of cases progress to MIBC. In contrast, MIBC is highly malignant and invasive, with a significant risk of metastasis and a five-year survival rate below 50% [2]. Recent advances in BLCA treatment have introduced novel therapeutic options, including PD-L1 inhibitors [3, 4], FGFR inhibitors [5], and antibody-drug conjugates (ADCs) [6]. Nevertheless, cisplatin- and gemcitabine-based chemotherapy remains the first-line treatment. Unfortunately, these strategies have yet to effectively curb BLCA progression or substantially improve overall survival rates, underscoring the critical need for new diagnostic biomarkers and therapeutic targets to overcome the persistent challenges in BLCA management.

To thrive in the nutrient-deprived microenvironment, tumor cells undergo metabolic reprogramming to secure the energy required for their growth and invasion. As one of the fourteen hallmarks of cancer [7], this reprogramming is predominantly characterized by aerobic glycolysis, commonly known as the Warburg effect, which is observed across various solid tumors. Despite the availability of oxygen, tumor cells favor glycolysis over oxidative phosphorylation, converting large amounts of glucose into lactate rather than fully oxidizing it to CO_2_ and H_2_O. This metabolic shift is driven by the overexpression of key glycolytic enzymes, such as hexokinase-2 (HK2), pyruvate kinase (PKM), and lactate dehydrogenase (LDHA), which enable the rapid conversion of pyruvate to lactate. This process alleviates mitochondrial stress and maintains redox homeostasis, facilitating tumor cell survival and proliferation.

The progression of BLCA is intrinsically linked to heightened glycolytic activity. Metabolomics studies reveal that BLCA cells consume significantly more pyruvate and secrete higher levels of lactate compared to normal bladder epithelial cells, a metabolic pattern associated with increased malignancy [8, 9]. Burns et al. demonstrated that BLCA cells with pronounced mesenchymal features exhibit accelerated lactate secretion and elevated LDHA expression [10]. Silencing LDHA selectively induces apoptosis in BLCA cells without affecting normal bladder epithelial cell viability. Similarly, Li et al. found that cisplatin-resistant BLCA cells display enhanced glycolytic activity, with increased lactate levels promoting H3K18 lactylation at the promoter regions of YY1 and YBX1, thereby contributing to cisplatin resistance [11]. CircXRN2 activates Hippo pathway by stabilizing LATS1, inhibiting glycolytic activity in bladder cancer (BLCA) cells and reducing lactate production, which leads to the inhibition of H3K18 lactylation modifications, subsequently inhibiting the progression of BLCA [12]. While inhibitors targeting glycolytic enzymes have shown potential in suppressing BLCA progression, their clinical application remains constrained. For instance, the traditional HK2 inhibitor 2-DG has been associated with adverse effects, including headaches and hyperglycemia [13]. Consequently, identifying novel molecular markers related to glycolysis in BLCA holds significant promise for overcoming current diagnostic and therapeutic challenges.

Cell Division Cycle Associated-3 (CDCA3), a critical subunit of the SCF-E3 ubiquitin ligase complex, facilitates the degradation of Wee1 and is indispensable in regulating the G2/M phase transition of the cell cycle. CDCA3 has been identified as a key driver in the progression of various malignancies [14–16]. In prostate cancer, CDCA3 accelerates cell cycle progression by activating the NF-κB signaling pathway, thereby promoting cellular proliferation [17]. In lymphoma, its expression is influenced by sphingolipid metabolism and targeted by SPHK2 [18]. Additionally, in ccRCC, long non-coding RNA SNHG12 stabilizes the SP1 protein, enhancing the transcriptional activation of *CDCA3* and contributing to sunitinib resistance [19]. In BLCA, *CDCA3* overexpression is driven by MYBL2 activation, underscoring its role in tumor progression [20, 21].

Intracellular metabolic patterns are closely intertwined with the cell cycle. As a pivotal component of the cyclin D-CDK4/6-Rb signaling pathway, CDK6 directly phosphorylates PFKP at S679 and PKM2 at S37, thereby increasing pentose phosphate metabolism and serine metabolism in tumor cells, elevating intracellular NADPH levels, and promoting tumor cell growth [22]. Furthermore, CDK9 mediates the phosphorylation of WTAP, facilitating its abnormal location in the cytoplasm, thereby alleviating the inhibition of CD36 and CCL2, promoting fatty acid uptake, and contributing to the progression of non-alcoholic steatohepatitis [23]. These findings prompt us to investigate whether CDCA3, beyond its primary role in cell cycle regulation, exerts a previously unrecognized regulatory effect on cell metabolism. Supporting this hypothesis, research on lung cancer hints at a potential role for CDCA3 in glycolysis regulation [24], though the precise mechanisms remain unclear. To better elucidate the role of CDCA3 in promoting BLCA progression, this study seeks to explicate the metabolic regulatory functions of CDCA3 and its underlying mechanisms.

Our findings demonstrate that CDCA3 regulates glycolysis in BLCA by transcriptionally activating *ENO1*. Furthermore, CDCA3 directly interacts with MYC and recruits TRIM28, mitigating MYC degradation through the ubiquitin-proteasome pathway. Concurrently, MYC enhances *CDCA3* transcription, forming a positive feedback loop that drives BLCA progression through ENO1-mediated glycolysis.

## Materials and methods

### Cell culture

All cell lines used in this study were obtained from the Cell Bank of the Chinese Academy of Sciences. T24 and 5637 BLCA cells were maintained in the 1640 medium, UM-UC3 BLCA cells in the MEM medium, and HEK293T cells in the DMEM medium. All media were supplemented with 10% fetal bovine serum (FBS) and 1% penicillin-streptomycin. T24 BLCA cells, derived from an 81-year-old female patient, represent high-grade BLCA. UM-UC3 BLCA cells, originating from an adult male patient, also represent high-grade BLCA. Meanwhile, 5637 BLCA cells, derived from a 68-year-old male patient, are indicative of low-grade BLCA.

### Nucleic acid transfection

Transfections were performed using the KeygenMAX3000 Reagent (KGA9705-1.5, KeyGen BioTECH). For siRNA transfection, a mixture of 5 µL siRNA and 5 µL MAX3000 reagent was combined with 200 µL Opti-MEM for approximately 2 × 10^5^ cells. For plasmid transfection, 1 µg of plasmid DNA was mixed with 5 µL MAX3000 reagent and 200 µL Opti-MEM for about 3 × 10^5^ cells. siRNAs were sourced from Hippo Biotechnology Co., Ltd. (Supplementary Table 1). Plasmids, including GFP-CDCA3, HA-MYC, Flag-TRIM28, truncated-CDCA3, and truncated-MYC constructs, were obtained from Juyan Biotechnology Co., Ltd., Hefei, China. The myc-Ub plasmid was generously provided by Dr. Jingtian Yu of Wuhan University [25], and the Flag-MYC plasmid was procured from Miaoling Co., Ltd., Wuhan, China. The Flag-ENO1 plasmid was used as previously reported [26] and the transfection efficiency was presented in Supplementary Fig. 1A.

### RNA extraction and qRT-PCR

Post-transfection, approximately 1.5 × 10^6^ cells were harvested and washed with PBS. Total RNA was extracted using the Sparkjade SPARKeasy Cell RNA Kit (AC0205-B) in accordance with the manufacturer’s instructions. qRT-PCR was performed using the TIANGEN FastReal SYBR Green Kit (FP217-01), with β-actin serving as the normalization control, and quantitative analysis conducted via the 2^−ΔΔCt^ method. Primer sequences are listed in Supplementary Table 2.

### Western blot (WB) and co-immunoprecipitation (Co-IP)

For protein analysis, transfected cells (approximately 1.5 × 10^6^) were collected, washed with PBS, and lysed with 200 µL RIPA buffer (BI-WB013, SBJbio) containing 1 mM PMSF (BI-WB081, SBJbio). Proteins were resolved via SDS-PAGE (GF1810, GeneFist, China).

Co-IP assays were performed on approximately 5 × 10^6^ cells using the BeaverBeads™ Protein A/G Immunoprecipitation Kit (22202-100, Beaver). Cell lysates were incubated with the primary antibody at 4 °C for 12 h, followed by incubation with Protein A/G magnetic beads for 1 h at 4 °C. The beads were washed five times with washing buffer, eluted in 1× loading buffer at 95 °C, and analyzed via WB.

To minimize interference from the IgG heavy chain, secondary antibodies (Abbkine, A25022) specific to avoid such cross-reactivity were used for WB analysis.

The list of antibodies utilized in this study is provided in Supplementary Table 3.

### Cell proliferation assays

For MTT assays, following transfection with siRNAs or plasmids, approximately 3000 cells were seeded per well in a 96-well plate. MTT solution (BS186, Biosharp, China) was added, and the cells were incubated for 3 h at 37 °C. Crystals formed were dissolved using 150 µL DMSO, and absorbance was measured at 570 nm.

For clone formation assays, transfected cells (approximately 1000 per well) were seeded in a 6-well plate and cultured under corresponding treatment conditions for 10 days. The cells were then fixed with 4% paraformaldehyde (PFA) for 30 min and stained with 0.1% crystal violet solution for 30 min.

### Measurement of ECAR and OCR

For Seahorse XFe96 metabolic analysis, 8000 cells per well were seeded in XF96 cell culture plates with 80 µL medium and incubated at 37 °C with 5% CO_2_ for 1 h, followed by the addition of 70 µL medium. Background wells received 150 µL medium without cells. After overnight incubation, the XF96 sensor cartridge was hydrated with 200 µL sterile water and incubated overnight at 37 °C without CO_2_. XF calibration fluid (20 mL) was prepared and pre-incubated under the same conditions. The medium’s pH was adjusted to 7.4, pre-warmed to 37 °C, and used to wash the wells (three times with 200 µL assay medium, leaving 20 µL to cover cells). Subsequently, 180 µL assay medium was added to each well, and the plate was incubated for 1 h at 37 °C without CO_2_. Drug solutions were prepared from concentrated stocks in an assay medium and sequentially loaded into sensor cartridge ports. The Seahorse XFe96 Analyzer was preheated and calibrated (~ 20 min) before analyzing metabolic parameters.

### Metabolite detection

Intracellular lactate and pyruvate levels were quantified using the Lactate Assay Kit (KTB1100, Abbkine, China) and the Pyruvate Acid Assay Kit (KTB1121, Abbkine, China), respectively.

Intracellular cholesterol level was quantified using the Amplex Red Cholesterol and Cholesterol Ester Detection Kit (Beyotime, S0211S, China).

### Flow cytometry assays

Cell apoptosis was evaluated using the Annexin V-FITC/PI Apoptosis Detection Kit (A5001-02P, Simubiotech, China). A total of 1 × 105 cells were collected and incubated with 100 µL Annexin V binding buffer containing 5 µL FITC and 5 µL PI reagent in the dark for 30 min. After incubation, 400 µL binding buffer was added to terminate the reaction, and samples were placed on ice for subsequent detection.

Intracellular reactive oxygen species (ROS) levels were measured using DCFH-DA (HY-D0940, MCE, USA). Approximately 1 × 105 cells were resuspended in 1 mL PBS containing 1 µM DCFH-DA and incubated in the dark for 30 min. After incubation, the samples were washed twice, resuspended in 1 mL PBS, and placed on ice for further analysis. Flow cytometry (FACS Fortessa, BD, USA) was used for detecting apoptosis and ROS levels.

### Molecular docking assay

Protein structural domains of CDCA3 and MYC were retrieved from the Protein Data Bank (PDB; http://www.rcsb.org/). Protein-protein interactions and visualizations were analyzed using PyMOL (Version 2.4) and PDBePISA (https://www.ebi.ac.uk/pdbe/pisa/).

### Dual-luciferase reporter assay

The CDCA3 promoter plasmid was utilized as previously described [21]. HEK293T cells were seeded into 24-well plates and cultured for 24 h. When the cell density reached approximately 50% confluence, 1 µg of HA-MYC plasmid or 1 µg of HA-Vector plasmid was co-transfected with 1 µg of the CDCA3 promoter plasmid. Following a 48-hour incubation, cells were gently washed twice with PBS and lysed at room temperature for 30 min. Luciferase activity was measured using the Dual-Luciferase^®^ Assay Kit (Cat. #E1910, Promega) in accordance with the manufacturer’s instructions.

### Chemicals

Details of all chemicals and their working concentrations are provided in Supplementary Table 4.

### Construction of risk predictive model

The GSE13507 and GSE32894 BLCA datasets were obtained from the GEO database, consisting of 165 and 224 BLCA samples with survival data, respectively. To eliminate batch effects between the datasets, we applied the “Combat” package to correct the expression matrix, thereby integrating the two datasets and obtaining expression data from a total of 389 BLCA patients for subsequent analysis. To investigate the prognostic value of the CDCA3 and ENO1 genes in bladder cancer patients, we performed univariate and multivariate Cox regression analyses using the survival and survminer packages. Based on the results of the multivariate Cox regression analysis, we developed a risk scoring model using the following formula: Risk score = (CDCA3 expression level × CDCA3 multivariate Cox regression coefficient) + (ENO1 expression level × ENO1 multivariate Cox regression coefficient). Patients were stratified into high- and low-risk groups based on the median risk score. Kaplan-Meier survival curves were then plotted to compare survival outcomes between the groups. Additionally, heatmaps were generated to visualize gene expression patterns across different risk groups. To enhance the clinical applicability of the model, we assessed the independent prognostic value of the risk score and clinical variables (including tumor grade, age, and gender) through univariate and multivariate Cox regression analyses. Based on the identified independent prognostic factors—risk score, tumor grade, and age—we constructed a nomogram for personalized prognosis prediction using the RMS package. To validate the predictive performance and practical applicability of the nomogram model, calibration curves and receiver operating characteristic (ROC) curves were generated using the timeROC package, ensuring an accurate assessment of the model’s reliability and discriminative power. Two patients were deleted due to the loss of pathological characteristics. Patients’ characteristics were listed in Supplementary Table 5.

### Gene set enrichment analysis (GSEA)

GSEA was performed to investigate the role of CDCA3 in BLCA. Data from the GSE32894 dataset and the Cancer Cell Line Encyclopedia (CCLE) were analyzed. Samples were classified into “High” and “Low” groups based on CDCA3 expression levels. Annotation files containing sample information were uploaded to GSEA software (version 4.3.3) for pathway enrichment analysis. Metabolism related hallmarks via GSE32894 and CCLE datasets were presented in Supplementary Tables 6&7.

### Construction of gemcitabine-resistant T24 BLCA cell lines

To establish gemcitabine-resistant T24 BLCA cell lines, parental T24 cells were treated with 2 µM gemcitabine for 48 h when cell confluence reached approximately 50%. After treatment, the medium containing gemcitabine was removed, and cells were washed three times with PBS before being cultured in a fresh medium. Once single colonies form, cells were collected and passaged for subsequent rounds of treatment. A total of 10 rounds of gemcitabine exposure were conducted to generate resistant cell lines.

### Construction of stable transfection cell lines with lentivirus

CDCA3-targeted lentivirus (shCDCA3), ENO1-targeted lentivirus (shENO1), and negative control lentivirus (shNC) were purchased from GenePharma (Shanghai, China). Following transfection with the respective lentivirus for 48 h, T24 BLCA cells were passaged, and puromycin (2 µg/mL) was added for further selection. The transfection efficiency of CDCA3 and ENO1 targeted lentivirus were validated via WB and qRT-PCR assays (Supplementary Fig. 1C-F). The sequences of the CDCA3- and ENO1-targeted lentivirus, along with the negative control lentivirus, are provided in Supplementary Table 1.

### In vivo experiments and immunohistochemistry (IHC)

The Ethics Committee for Experimental Animals at The First Affiliated Hospital of the University of Science and Technology of China approved this study (Approval Number: 202404122018000509629).

Male BALB/c nude mice, purchased from Zhejiang Vital River Laboratory Animal Technology Co., Ltd., were randomly assigned to experimental groups. A total of 5 × 10^6^ shNC or shCDCA3 T24 cells were subcutaneously injected into the mice. Xenograft sizes were measured every three days starting six days post-injection, and tumor volume was calculated using the formula V = (length × width^2^)/2. Drug treatment regimens are detailed in the corresponding figures. All mice were sacrificed on day 30 following xenografting, after which tumor weight and volume were recorded. IHC assays were conducted as previously described [26].

### LC/MS analysis for CDCA3-interacted proteins

Protein samples were mixed with 200 µL of 8 M urea in Nanosep devices and centrifuged at 12,000 g for 20 min at 20 °C. Reduction was carried out by adding 200 µL of 8 M urea containing 10 mM DTT, followed by incubation at 37 °C for 2 h. After solution removal by centrifugation, 200 µL of 8 M urea with 50 mM IAA was added, and samples were incubated in the dark for 15 min. Following multiple washes, digestion was initiated by adding 100 µL of 25 mM ammonium bicarbonate containing 0.01 µg/µL trypsin and incubating for 12 h. The final washes were centrifuged, and peptide fractions were collected and lyophilized. Peptides were re-suspended in ultrapure water with 0.1% formic acid and loaded onto a nanoViper C18 trap column. Chromatographic separation was performed using an Easy nLC 1000 system with a 90-minute elution gradient. Tandem MS data were acquired on a ThermoFisher Fusion mass spectrometer and analyzed for protein identification using PEAKS Online 11.

### Statistical analysis

Statistical analyses were conducted using GraphPad Prism (version 10.1.3). Results from three independent experiments are expressed as mean ± standard deviation (SD). Statistical significance was assessed using two-tailed Student’s t-tests or one-way ANOVA, with *p* < 0.05 considered statistically significant.

**Fig. 1:**
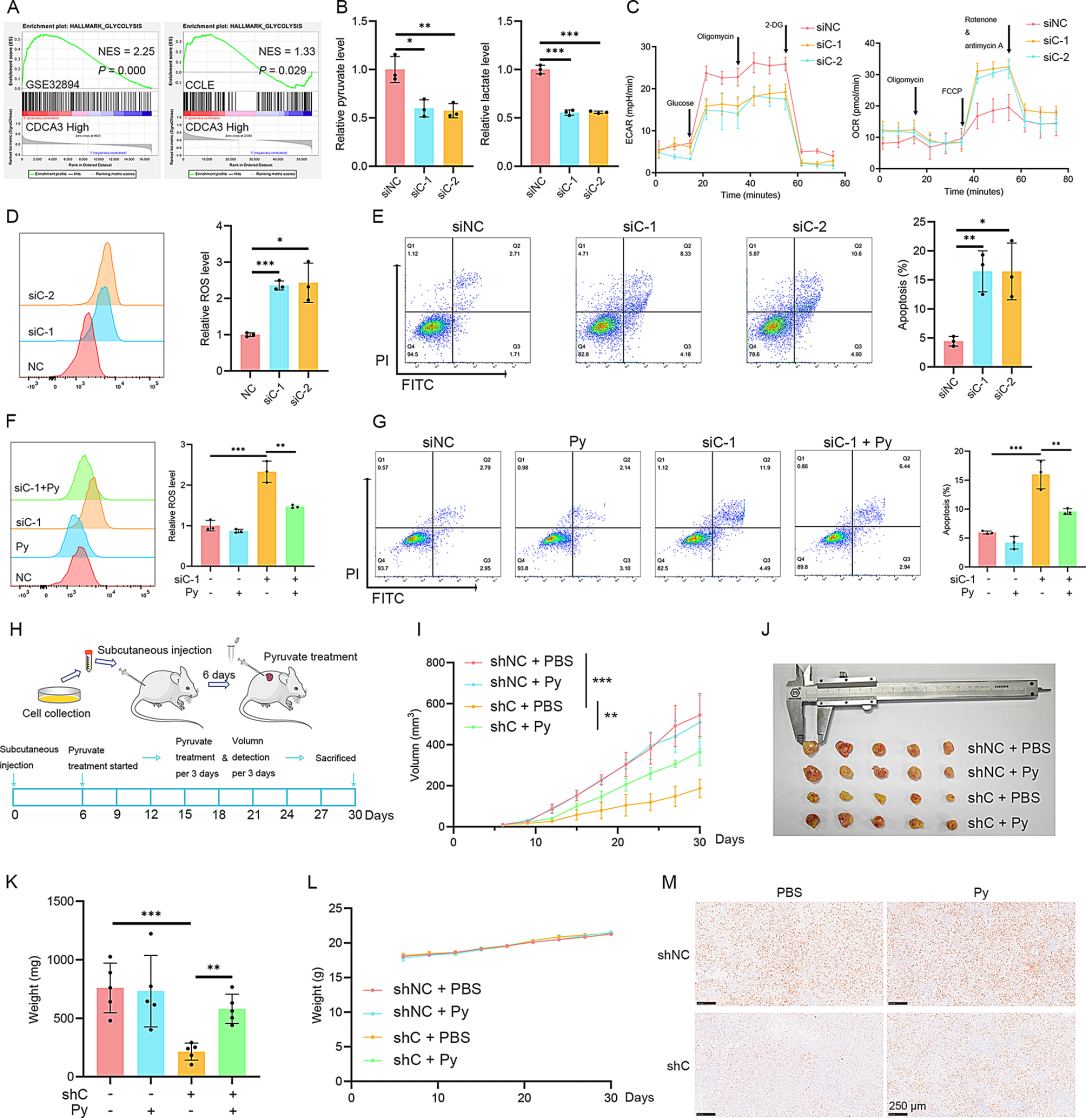
CDCA3 promotes BLCA progression via enhancing glycolysis. **(A**) GSEA enrichment analysis of CDCA3 in BLCA cohorts. **(B)** Assessment of intracellular levels of pyruvate and lactate in T24 cells following CDCA3 silencing (*n* = 3). **(C)** Assessment of extracellular acidification rate (ECAR) and oxygen consumption rate (OCR) in T24 cells following CDCA3 silencing (*n* = 3). **(D)** Assessment of intracellular ROS levels following CDCA3 silencing in T24 cells (*n* = 3). **(E)** Assessment of apoptosis following CDCA3 silencing in T24 cells (*n* = 3). **(F)** Assessment of intracellular ROS levels following the addition of pyruvate (2 mM) to CDCA3-silenced T24 cells (*n* = 3). **(G)** Assessment of apoptosis following the addition of pyruvate (2 mM) to CDCA3-silenced T24 cells (*n* = 3). **(H)** Construction of an in vivo model and subsequent pyruvate treatment. **(I)** Changes in the volume of xenograft tumors across each group (*n* = 5). **(J)** Overview of dissected tumors from each group (*n* = 5). **(K)** Weights of the xenograft tumors from each group (*n* = 5) following dissection. **(L)** Changes in body weights of mice across each group (*n* = 5). **(M)** Immunohistochemical staining of Ki-67 of xenograft tissues

## Results

### CDCA3 promotes BLCA proliferation via enhancing glycolysis

Analysis of the GSEA results from the GSE32894 BLCA dataset and the CCLE dataset reveal that CDCA3 is critically involved in the regulation of glycolysis in BLCA (Fig. 1A). Consistent with these observations, silencing CDCA3 significantly reduces intracellular pyruvate and lactate levels (Fig. 1B; Supplementary 2 A), suppresses the extracellular acidification rate (ECAR), and increases the oxygen consumption rate (OCR) (Fig. 1C). Conversely, overexpression of CDCA3 elevates intracellular pyruvate and lactate levels in both T24 BLCA cells and 5637 BLCA cells (Supplementary Fig. 2B, C). Given the indispensable role of glycolysis in maintaining redox homeostasis and cell survival, the impact of CDCA3 silencing on intracellular ROS levels was further investigated. Flow cytometry analysis demonstrates that silencing CDCA3 leads to an abnormal accumulation of ROS and induces apoptosis in BLCA cells (Fig. 1D, E; Supplementary Fig. 2D, E).

**Fig. 2:**
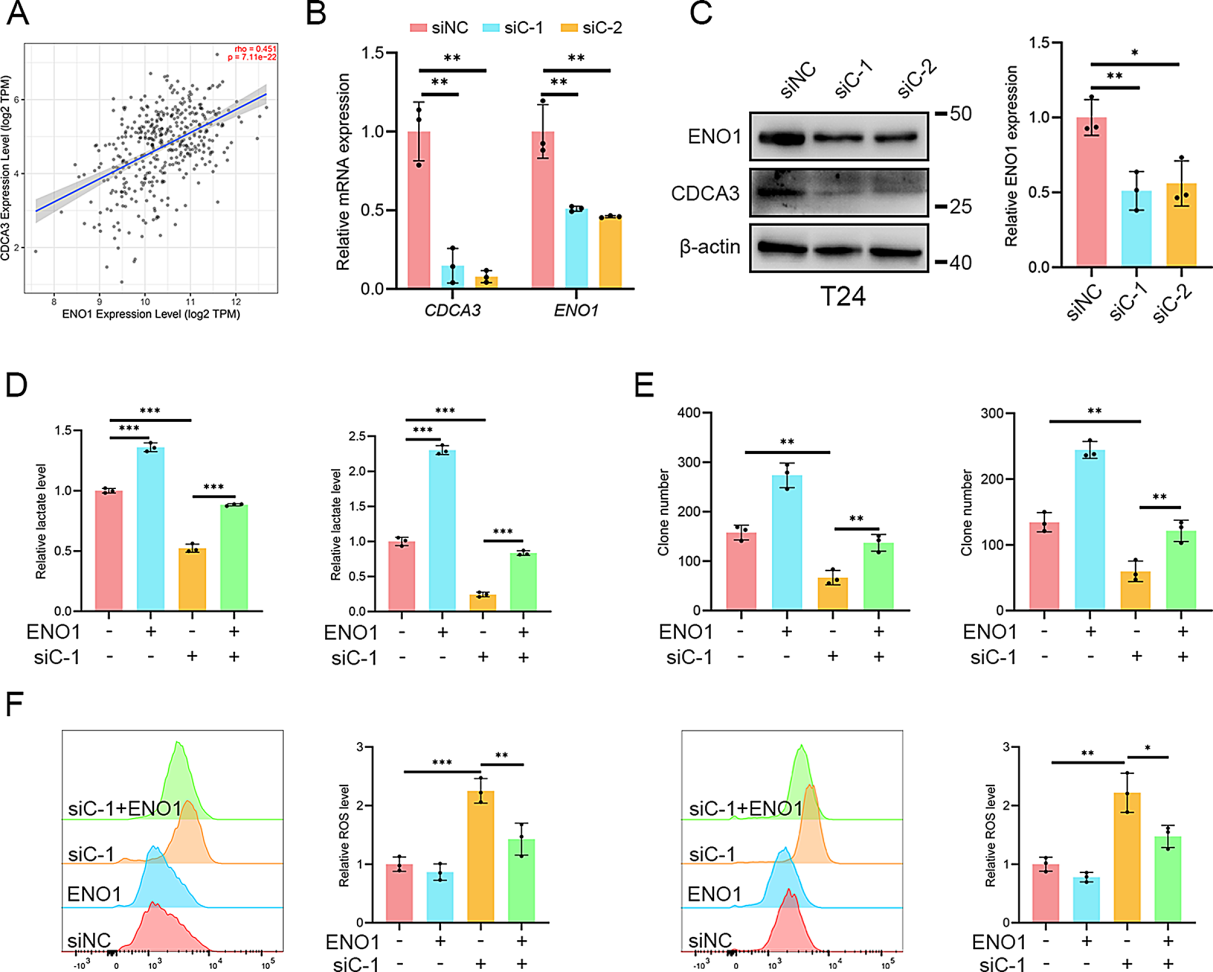
CDCA3 enhances BLCA glycolysis via α-Enolase. **(A)** Correlation of *CDCA3* and *MYC* in TIMER 2.0 database. **(B)** qRT-PCR assays measuring the mRNA levels of *ENO1* following CDCA3 silencing in T24 BLCA cells. **(C)** WB assays measuring the protein levels of ENO1 following CDCA3 silencing in T24 BLCA cells and densitometric analysis. **(D)** Assessment of intracellular levels of lactate in T24 and 5637 BLCA cells following CDCA3-silencing and ENO1-overexpressing. **(E)** Statistics of clone formation assays performed in T24 and 5637 BLCA cells following CDCA3-silencing and ENO1-overexpressing. **(F)** Assessment of intracellular ROS level in T24 and 5637 BLCA cells following CDCA3-silencing and ENO1-overexpressing

**Fig. 3:**
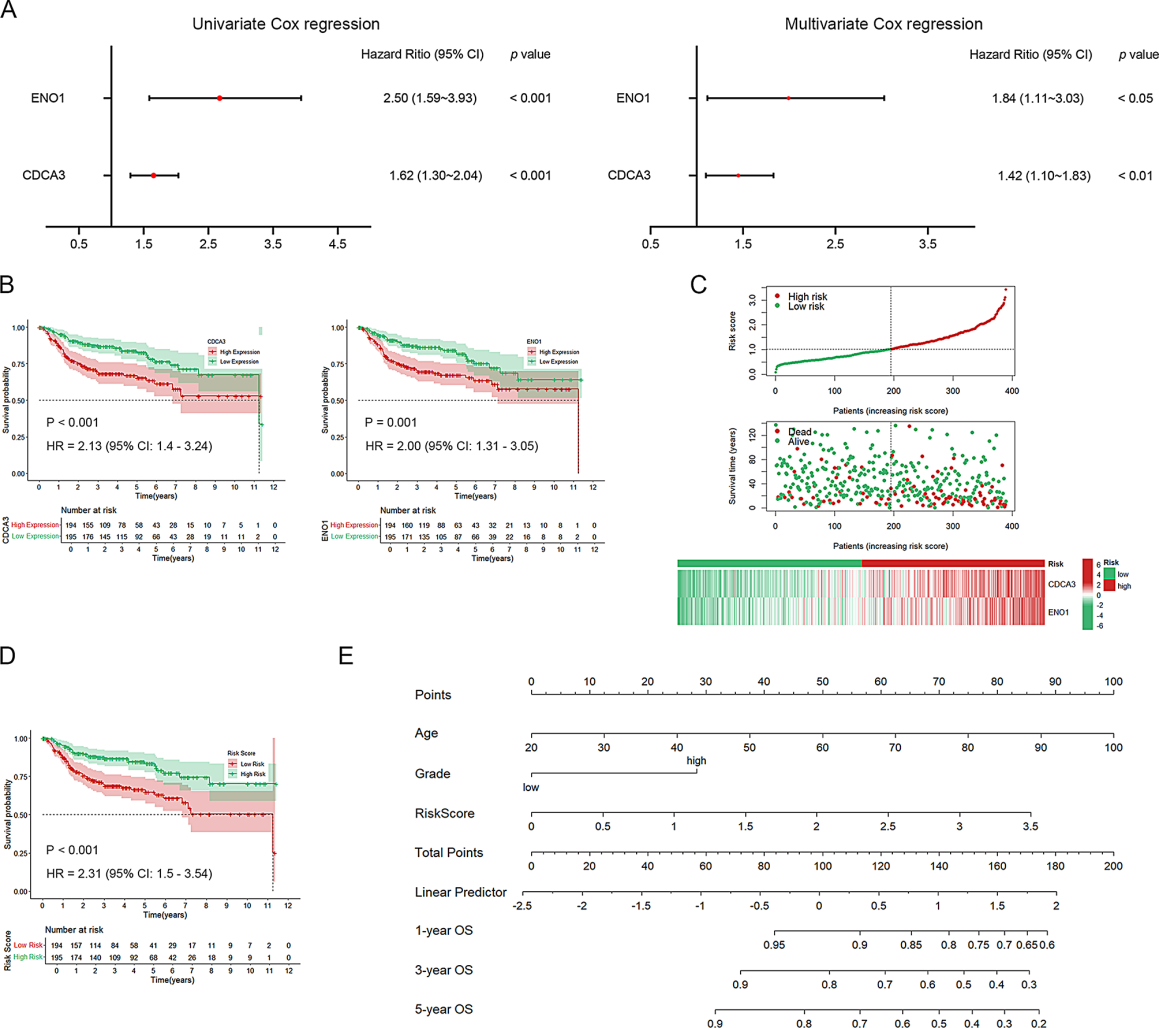
Construction of a risk predictive model. **(A)** Univariate Cox and multivariate Cox regression analyses were performed for *CDCA3* and *ENO1*, respectively. **(B)** K-M curve to analyze the relationship between *CDCA3*/*ENO1* and OS of BLCA patients in the model. **(C)** Heatmap of *CDCA3* and *ENO1* gene expression, risk score curve and scatter plot of survival status in BLCA patients. **(D)** K-M analysis of BLCA patients in the high- and low-risk groups. **(E)** A nomogram model was constructed to predict 1-, 3-, and 5-year survival of BLCA patients based on three independent prognostic factors (risk score, age and grade)

To confirm that CDCA3 promotes BLCA cell proliferation by enhancing glycolysis, pyruvate, a terminal metabolite of glycolysis, was supplemented to simulate an enhanced-glycolysis condition. Supplementing pyruvate effectively mitigates the redox imbalance and reduces cell death caused by CDCA3 silencing (Fig. 1F, G; Supplementary Fig. 2F, G). Clone formation assays further show that pyruvate supplementation rescues the proliferative capacity of BLCA cells following CDCA3 silencing (Supplementary Fig. 2H). Additionally, in vivo experiments demonstrate that silencing CDCA3 inhibits T24 BLCA tumor growth, whereas intraperitoneal injection of pyruvate restores the proliferation of sh-CDCA3 T24 cells (Fig. 1H-M). Moreover, considering that pyruvate is essential for various metabolic pathways, we analyzed the changes in other metabolites after silencing CDCA3 and supplementing pyruvate. We measured intracellular cholesterol levels, since it is suggested to be regulated by CDCA3, according to Supplementary Tables 6&7. However, silencing CDCA3 resulted in only a minimal decrease in cholesterol, and pyruvate supplementation did not restore cholesterol levels (Supplementary Fig. 3A), but effectively restored the reduced lactate level (Supplementary Fig. 3B). These results establish that CDCA3 regulates glycolysis in BLCA cells and promotes their proliferation through its glycolytic-enhancing effects.

**Table 1:** Correlation between *CDCA3* and glycolysis enzymes

	TIMER 2.0 (TCGA)		GSE13507		GSE32894	
	Cor	*p*	Cor	*p*	Cor	*p*
*GLUT1*	0.014	0.78	0.4061	< 0.001	-0.1685	0.003
*HK2*	0.02	0.68	0.2559	< 0.001	0.0067	0.9072
*GPI*	0.25	< 0.001	**0.5108**	**< 0.001**	0.1835	0.0012
*PFKP*	0.24	< 0.001	0.0585	0.3524	0.1175	0.0392
*ALDOA*	0.037	0.46	0.1137	0.0129	0.05633	0.3245
*TPI1*	**0.59**	**< 0.001**	0.1978	0.0015	**0.4778**	**< 0.001**
*GAPDH*	**0.40**	**< 0.001**	0.4641	< 0.001	0.1723	0.0024
*PGK1*	0.31	< 0.001	0.1122	0.0736	0.1043	0.0676
*PGAM1*	0.17	< 0.001	Not found		-0.0295	0.6057
*ENO1*	**0.45**	**< 0.001**	**0.6572**	**< 0.001**	**0.4444**	**< 0.001**
*PKM2*	0.07	0.19	0.2257	< 0.001	-0.0901	0.1147
*LDHA*	0.31	< 0.001	0.3852	< 0.001	**0.1960**	**< 0.001**
*LDHB*	0.09	0.06	-0.1146	0.0678	-0.1807	0.0015

### CDCA3 enhances BLCA glycolysis via α-Enolase

To elucidate the mechanism by which CDCA3 enhances glycolysis in BLCA, the potential regulation of glycolytic enzyme expression by CDCA3 was investigated. Correlation analysis at the transcriptional level across multiple public BLCA datasets identified ENO1 as consistently positively correlated with CDCA3 (Table 1; Fig. 2A). Given that ENO1 has been reported to facilitate BLCA progression [26], its role in mediating the effects of CDCA3 on glycolysis was further explored. qRT-PCR and WB assays revealed that silencing CDCA3 significantly reduces ENO1 expression (Fig. 2B, C; Supplementary Fig. 4A). Metabolite analysis demonstrated that ENO1 overexpression rescues the reduced lactate levels caused by CDCA3 silencing (Fig. 2D). Similarly, clone formation assays showed that overexpressing ENO1 restores the proliferative capacity of BLCA cells impaired by CDCA3 silencing (Fig. 2E; Supplementary Fig. 4B). Additionally, ENO1 overexpression mitigates the intracellular redox imbalance induced by CDCA3 silencing (Fig. 2F). To determine whether CDCA3-mediated regulation of glycolysis depends on ENO1, we generated stable ENO1-silenced cells and subsequently silenced or overexpressed CDCA3 to examine changes in intracellular lactate levels. The results indicated that, in the absence of ENO1, CDCA3 silencing did not further reduce lactate levels in sh-ENO1 T24 cells, and CDCA3 overexpression failed to restore lactate levels (Supplementary Fig. 4C, D). Collectively, these findings demonstrate that ENO1 mediates CDCA3’s effect on glycolysis in BLCA.

**Fig. 4:**
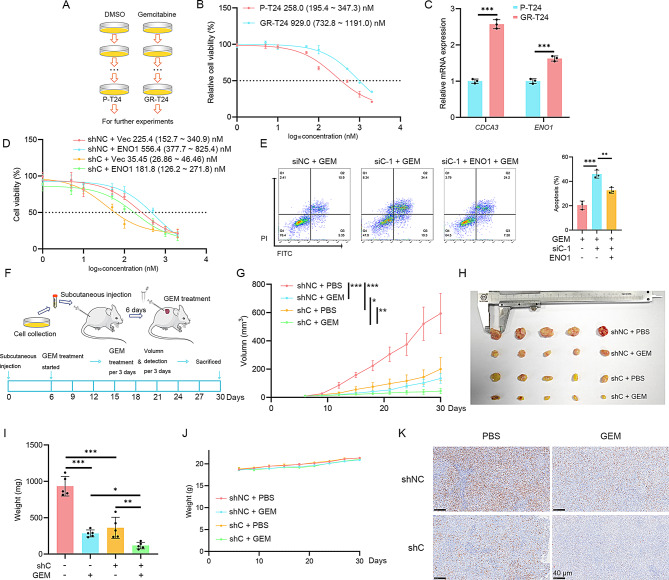
Targeting CDCA3-ENO1 axis is able to promote gemcitabine chemotherapy. **(A)** Methods of construction of gemcitabine-resistant T24 BLCA cells. **(B)** MTT assays for detecting the sensitivity of P-T24 and GR-T24 cells to gemcitabine. **(C)** The mRNA level of *CDCA3* and *ENO1* in P-T24 and GR-T24 cells. **(D)** Cell viability of shNC T24 cells and shCDCA3 T24 cells with or without ENO1-overexpressing under 48 h GEM treatment. **(E)** Apoptosis analysis of T24 cells with silencing CDCA3 and (or) overexpressing ENO1 under 48 h of 500 nM GEM treatment (48 h, *n* = 3). **(F)** Construction of an in vivo model and subsequent GEM treatment. **(G)** Changes in the volume of xenograft tumors across each group (*n* = 5). **(H)** Overview of dissected tumors from each group (*n* = 5). **(I)** Weights of the xenograft tumors from each group following dissection (*n* = 5). **(J)** Changes in body weights of mice across each group (*n* = 5). **(K)** Immunohistochemical staining of Ki-67 of xenograft tissues

**Fig. 5:**
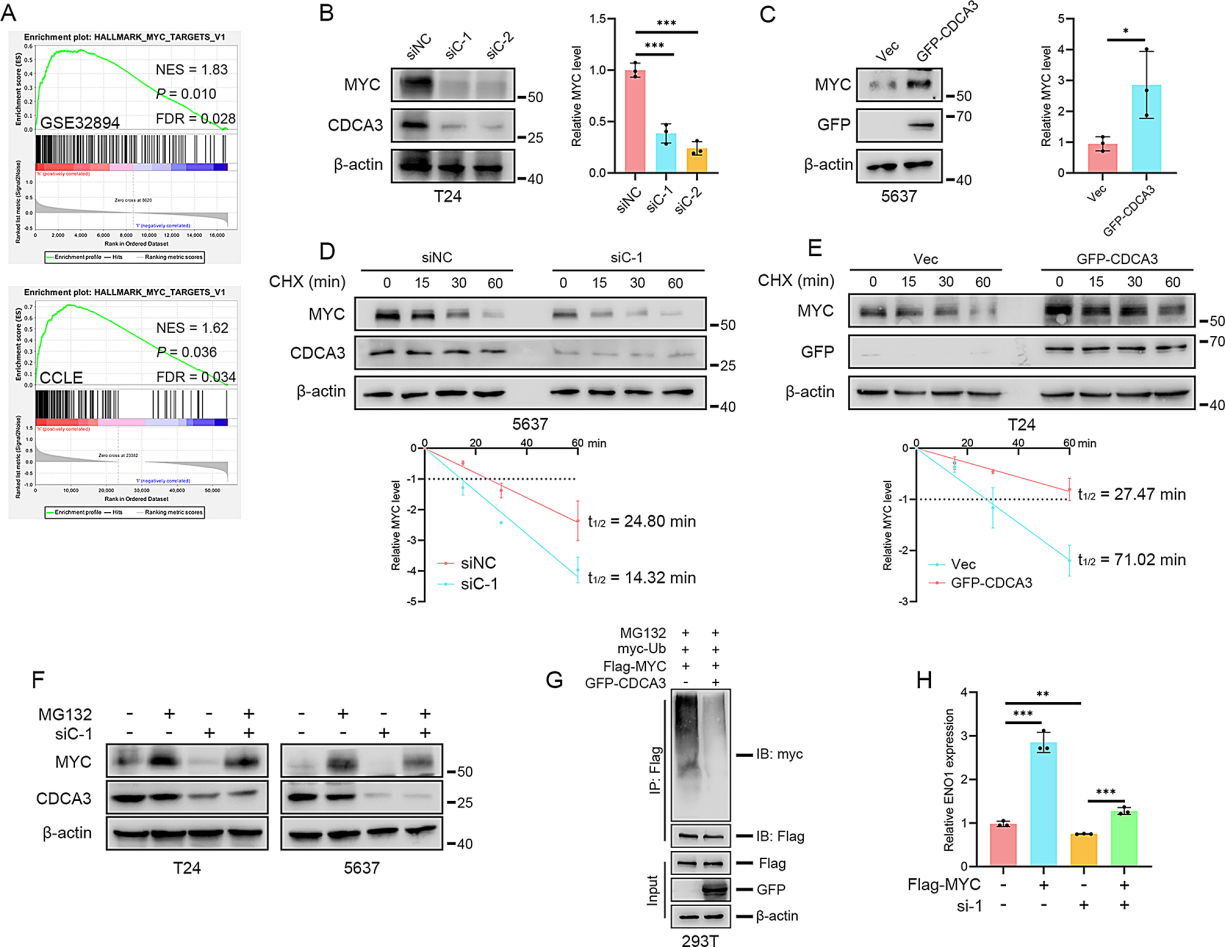
CDCA3 activates ENO1 transcription via maintaining MYC protein stability. **(A)** GSEA enrichment analysis of CDCA3-related pathways in BLCA cohorts. **(B)** WB assays of MYC and CDCA3 protein levels following CDCA3 silencing in T24 BLCA cells and densitometric analysis. **(C)** WB assays of MYC and CDCA3 protein levels following CDCA3 overexpression in 5637 BLCA cells and densitometric analysis. **(D)** WB assays assessing the effect of CDCA3 silencing on MYC protein stabilization in 5637 BLCA cells following CHX treatment (50 µg/mL) and densitometric analysis. **(E)** WB assays assessing the effect of CDCA3 overexpression on MYC protein stabilization in T24 BLCA cells following CHX treatment (50 µg/mL) and densitometric analysis. **(F)** WB assays assessing MYC protein levels following treatment of CDCA3-silenced BLCA cells with MG132 (10 µM, 4 h). **(G)** Ubiquitination assay assessing the exogenous ubiquitination levels of MYC following CDCA3 overexpression in 293T cells. **(H)** qRT-PCR assays measuring the mRNA levels of *ENO1* following MYC overexpression in CDCA3-silenced 5637 BLCA cells (*n* = 3)

The potential utility of CDCA3 and ENO1 as predictive biomarkers for BLCA was also evaluated. Integration of two BLCA datasets with survival data, followed by univariate Cox analysis, identified both genes as high-risk factors for BLCA. Multivariate Cox analysis further established that CDCA3 and ENO1 are independent prognostic indicators (Fig. 3A). Kaplan-Meier survival analysis indicated that high expression of either gene correlates with poorer prognosis (Fig. 3B). A risk prediction model was constructed based on the formula: Risk score = *CDCA3* × 0.3492 + *ENO1* × 0.6084, and patients were stratified into high- and low-risk groups. Heat maps of the risk score, survival status, and gene expression profiles demonstrated a higher mortality rate in the high-risk group (Fig. 3C, D). The AUC value of ROC curve analysis of the survival model to predict the overall survival status at 1-, 3-, and 5-year reach 0.708, 0.686 and 0.685, which indicated robust predictive performance (Supplementary Fig. 5A). Additionally, a nomogram incorporating the risk score, age, and tumor grade was constructed for a cohort of 387 patients, further demonstrating the predictive capacity of the model (Fig. 3E). Calibration plots analysis of each fitted line almost overlapped with the standard curve, confirming the model’s excellent accuracy (Supplementary Fig. 5B). The AUC value of ROC curve analysis of the nomogram model at 1-, 3-, and 5-year reach 0.769, 0.751 and 0.750, also presenting the ideal sensitivity and specificity of our predictive model (Supplementary Fig. 5C).

**Fig. 6:**
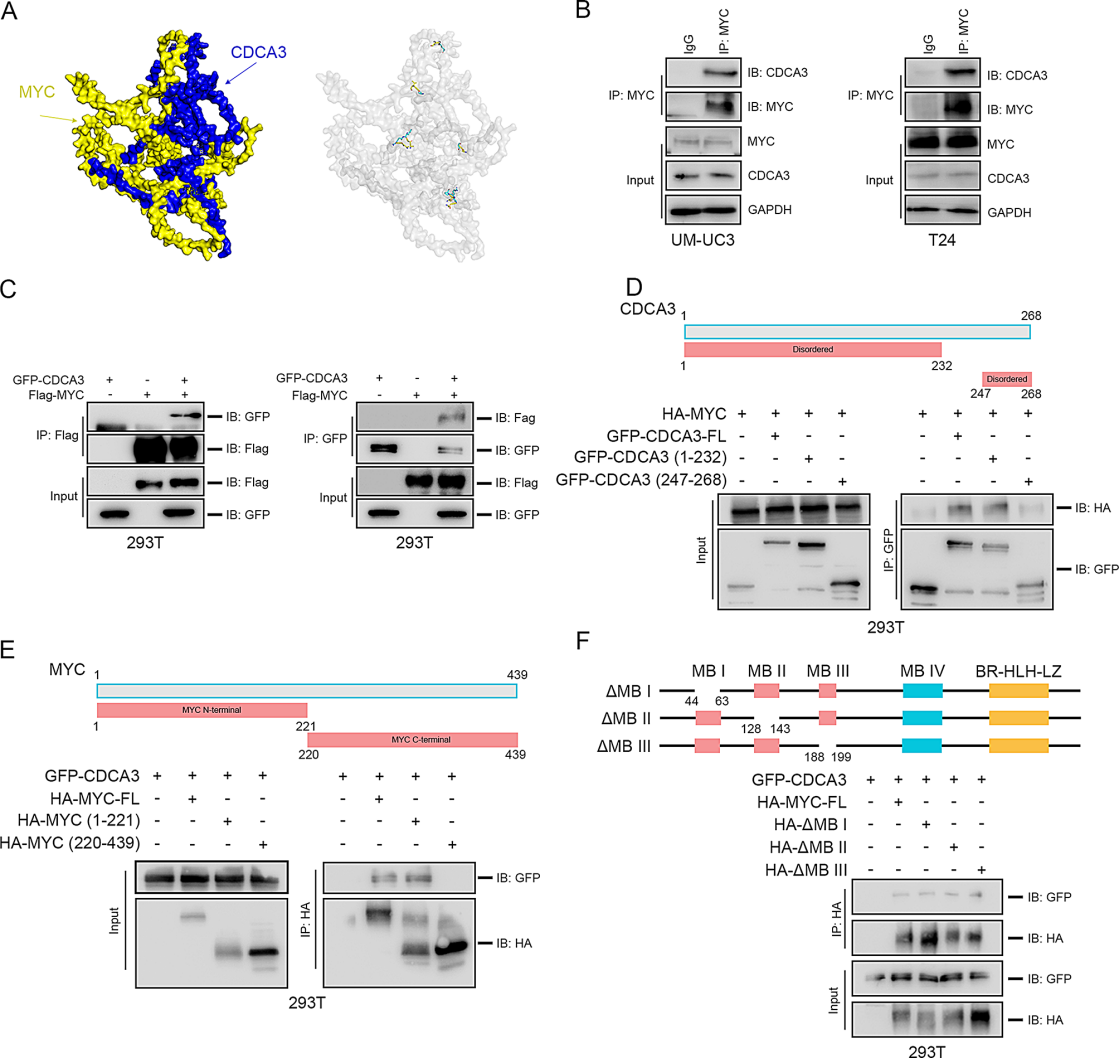
CDCA3 binds to MYC. **(A**) Diagram of the docking model and the interfacing residues between CDCA3 and MYC protein (CDCA3, blue; MYC, yellow). **(B)** Endogenous Co-IP assays detecting the interaction of CDAC3 and MYC performed in UM-UC3 (left panel) and T24 (right panel) BLCA cells, housekeeping protein GAPDH was used to verify loading consistency. **(C)** Exogenous Co-IP assays detecting the interaction of GFP-CDAC3 and Flag-MYC performed in 293T cells. **(D)** Exogenous Co-IP assays performed to detect the specific region of CDCA3 interacted with MYC in 293T cells. **(E)** Exogenous Co-IP assays performed to detect the specific region of MYC interacted with CDCA3 in 293T cells. **(F)** Exogenous Co-IP assays performed to detect the interaction between CDCA3 and various MYC deletion mutations in 293T cells

**Fig. 7:**
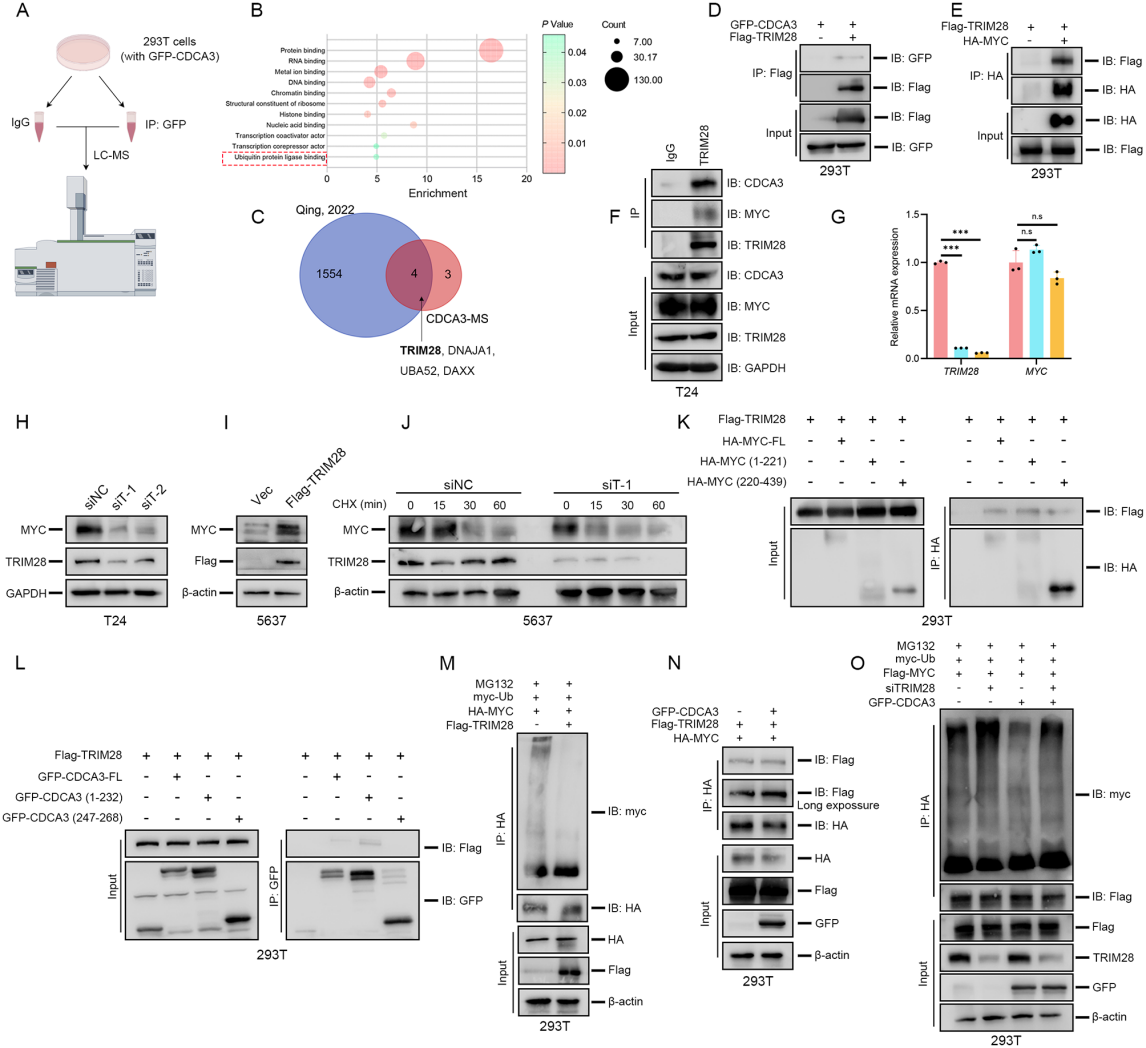
CDCA3 recruits TRIM28 to stabilize MYC. **(A**) Diagram of performing LS/MS analysis. **(B)** MF enrichment analysis of protein that interact with CDCA3. **(C)** Intersection of 7 CDCA3-interacted ubiquitin ligase and MYC-interacted proteins. **(D)** Exogenous Co-IP assays for detecting the interaction of GFP-CDAC3 and Flag-TRIM28 performed in 293T cells. **(E)** Exogenous Co-IP assays for detecting the interaction of HA-MYC and Flag-TRIM28 performed in 293T cells. **(F)** Endogenous Co-IP assays detecting the interaction of TRIM28, CDAC3 and MYC performed in T24 BLCA cells, housekeeping protein GAPDH was used to verify loading consistency. **(G)** qRT-PCR results of *MYC* and *TRIM28* mRNA levels following TRIM28 silencing in T24 BLCA cells. **(H)** WB results of MYC and TRIM28 protein levels following TRIM28-silencing in T24 BLCA cells. **(I)** WB results of MYC and TRIM28 protein levels following TRIM28-overexpressing in 5637 BLCA cells. **(J)** WB assays assessing the effect of TRIM28 silencing on MYC protein stabilization in T24 BLCA cells following CHX treatment (50 µg/mL). **(K)** Exogenous Co-IP assays performed to detect the specific region of MYC interacted with TRIM28 in 293T cells. **(L)** Exogenous Co-IP assays performed to detect the specific region of CDCA3 interacted with TRIM28 in 293T cells. **(M)** Ubiquitination assays assessing the exogenous ubiquitination levels of MYC following TRIM28 overexpression in 293T cells. **(N)** Exogenous Co-IP assays for detecting the interaction of HA-MYC and Flag-TRIM28 with co-transfecting GFP-CDCA3 performed in 293T cells. **(O)** Ubiquitination assays assessing the exogenous ubiquitination levels of MYC following TRIM28-silencing and GFP-CDCA3-overexpression in 293T cells

**Fig. 8:**
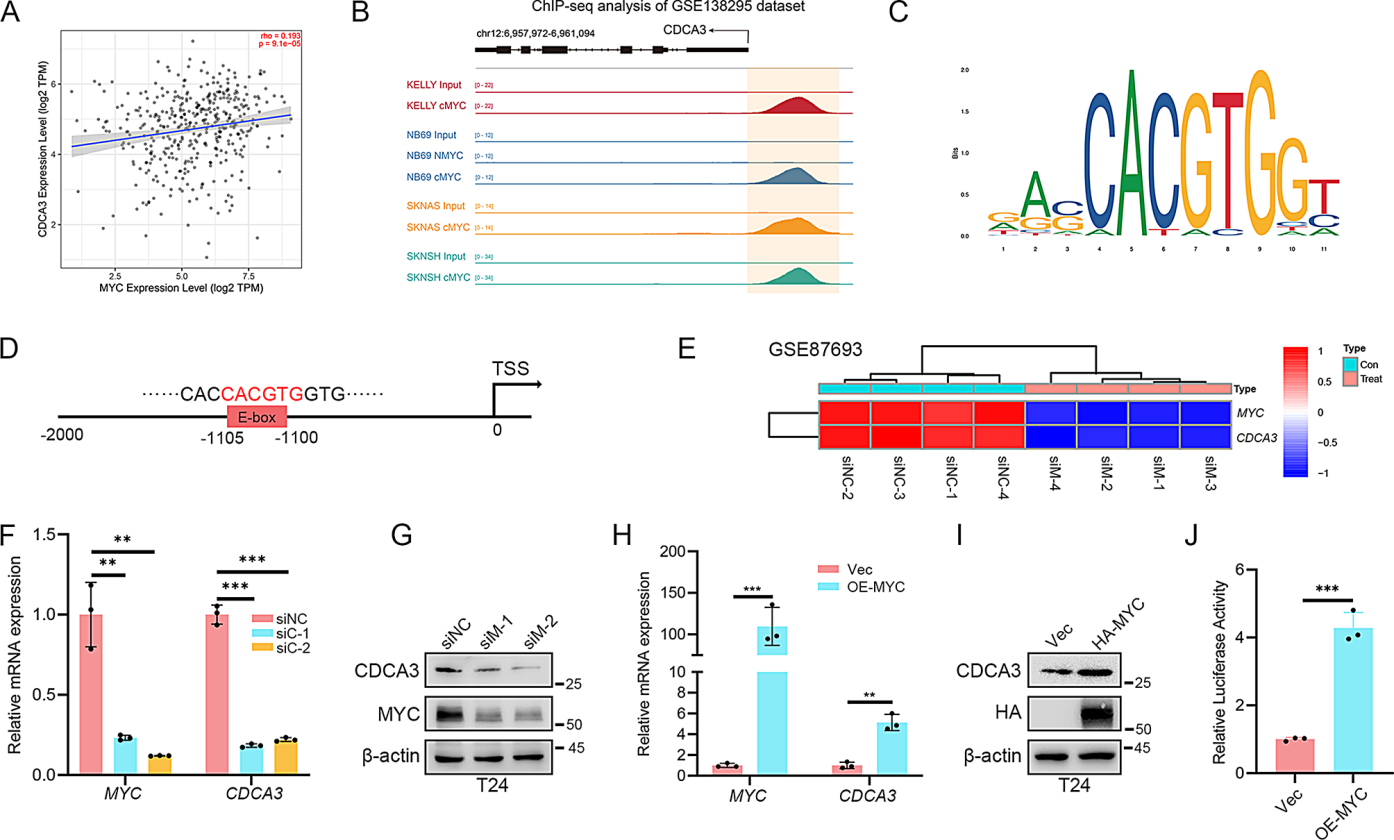
MYC promotes *CDCA3* transcription. **(A**) Spearman correlation analysis of the mRNA level of *CDCA3* and *MYC* from TIMER 2.0 database. **(B)** Genome browser tracks of MYC or NMYC occupancy in the *CDCA3* loci in SKNSH, SKNAS or NB69 cells (public dataset: GSE138295). **(C)** The binding sequences of MYC on the promoter region of targets genes. **(D)** The E-box site located on *CDCA3* promoter region. **(E)** A heatmap of *CDCA3* and *MYC* upon *MYC* knockdown in lymphoma Jurkat cells (*n* = 3). **(F)** qRT-PCR results of *CDCA3* and *MYC* mRNA level after silencing *MYC* in T24 BLCA cells (*n* = 3). **(G)** WB results of CDCA3 and MYC protein level after silencing MYC in T24 BLCA cells. **(H)** qRT-PCR of *CDCA3* and *MYC* mRNA level after overexpressing *MYC* in T24 BLCA cells (*n* = 3). **(I)** WB of CDCA3 and MYC protein level after overexpressing MYC in T24 BLCA cells. **(J)** Luciferase reporter assays of the CDCA3 promoter in Vector and HA-MYC-overexpressing 293T cells

**Fig. 9:**
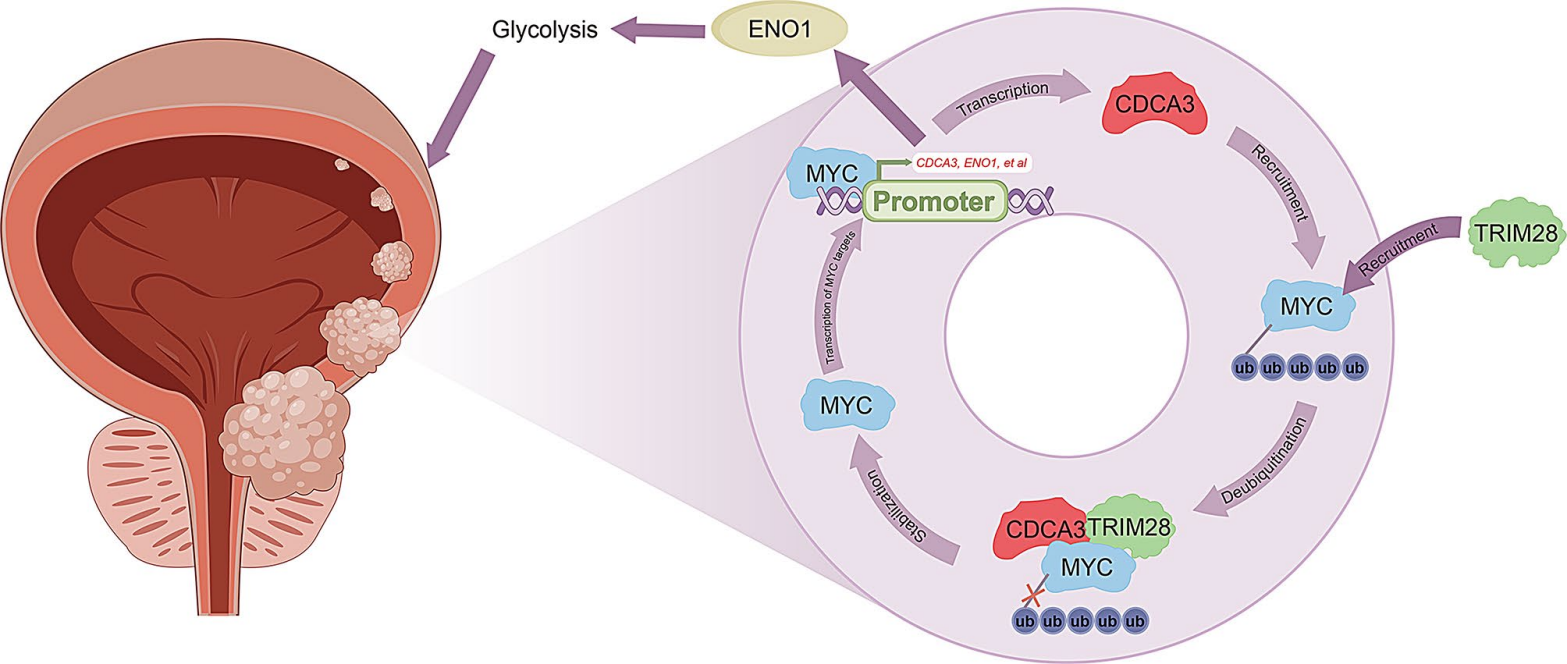
A simplified schematic diagram showing the CDCA3-MYC positive feedback loop promoting BLCA progression via ENO1-mediated glycolysis. CDCA3 stabilizes MYC by recruiting TRIM28 to decrease the ubiquitination of MYC. In turn, amplificated MYC transcriptionally activates *CDCA3*. The reciprocally enhanced CDCA3-MYC positive feedback loop promotes BLCA progression via ENO1-mediacted glycolysis

### Targeting CDCA3-ENO1 axis is able to promote gemcitabine chemotherapy

Previous studies have established that enhanced glycolysis and upregulated ENO1 are critical in conferring chemotherapy resistance [27, 28]. To investigate the role of the CDCA3-ENO1 axis in the chemotherapy response of BLCA, a gemcitabine-resistant T24 cell line (GR-T24) was generated (Fig. 4A, B), which exhibited elevated expression levels of both *CDCA3* and *ENO1* (Fig. 4C). IC50 curve analysis demonstrated that CDCA3-silenced cells (shCDCA3) showed higher sensitivity to GEM treatment, while overexpressing ENO1 partially restored the sensitivity (Fig. 4D). Apoptosis analysis also show that silencing CDCA3 cause more cell death under GEM treatment, while overexpressing ENO1 mitigated the effect (Fig. 4E). In vivo assays further confirmed that CDCA3 silencing enhances the efficacy of gemcitabine in BLCA cells. Cells transfected with shCDCA3-lentivirus displayed markedly reduced growth and significantly greater sensitivity to gemcitabine compared to cells transfected with shNC-lentivirus (Fig. 4F-J). IHC assays revealed a substantial reduction in Ki67 staining in shCDCA3-lentivirus transfected cells, indicating decreased proliferation (Fig. 4K). These results suggest that the CDCA3-ENO1 axis represents a potential therapeutic target for enhancing the efficacy of gemcitabine chemotherapy in BLCA.

### CDCA3 activates *ENO1* transcription via maintaining MYC protein stability

Given that CDCA3 is not classified as a transcription factor (TF), it was hypothesized that its regulation of *ENO1* transcription occurs via a specific TF. GSEA results revealed a strong association between CDCA3 and the MYC-related signaling pathway (Fig. 5A). Considering MYC’s pivotal role in regulating glucose metabolism and its established function as a transcriptional activator of *ENO1* [29], further exploration of CDCA3’s influence on the MYC pathway was undertaken. WB assays indicated that silencing CDCA3 reduces MYC protein levels, whereas CDCA3 overexpression increases MYC protein levels (Fig. 5B, C; Supplementary Fig. 5B, C). In contrast, qRT-PCR results demonstrated no significant impact of CDCA3 on *MYC* mRNA levels (Supplementary Fig. 6A). Notably, silencing CDCA3 markedly accelerated MYC protein degradation, while CDCA3 overexpression enhanced its stability (Fig. 5D, E; Supplementary Fig. 6D, E). The application of MG-132, a selective 26 S proteasome inhibitor, effectively prevented the reduction in MYC levels induced by CDCA3 silencing (Fig. 5F). Ubiquitination assays further revealed that CDCA3 overexpression reduces MYC ubiquitination (Fig. 5G), whereas silencing CDCA3 increases MYC ubiquitination (Supplementary Fig. 6F). Additionally, MYC overexpression restored *ENO1* mRNA levels that were diminished by CDCA3 knockdown (Fig. 5H). These results underscore the critical role of MYC in mediating the transcriptional regulation of *ENO1* by CDCA3.

### CDCA3 recruits TRIM28 to decrease MYC ubiquitination

Molecular docking analysis predicts a potential interaction between CDCA3 and MYC (Fig. 6A), which was validated by Co-IP assays demonstrating an endogenous interaction in UM-UC3 and T24 BLCA cells (Fig. 6B). Further confirmation was obtained through co-transfection of GFP-CDCA3 and Flag-MYC plasmids in 293T cells, supporting this interaction (Fig. 6C). Structural prediction indicates that full-length CDCA3 contains two “Disordered” domains at the N- and C-termini. Subsequent Co-IP assays identified that MYC interacts with the N-terminal “Disordered” domain of CDCA3 (Fig. 6D) and that CDCA3 associates with the N-terminus of MYC (Fig. 6E). Interestingly, the absence of a specific region at the N-terminus of MYC did not affect its interaction with CDCA3 (Fig. 6F), suggesting that CDCA3 does not directly interfere with the recognition of MYC by E3 ubiquitin ligases such as FBXW7 or SKP2. These results indicate that CDCA3 likely diminishes MYC ubiquitin-mediated degradation by recruiting a specific protein to reduce MYC ubiquitination.

To identify potential CDCA3-associated proteins involved in regulating MYC ubiquitination, a GFP-CDCA3 plasmid was transfected into 293T cells, followed by Co-IP and liquid chromatography-mass spectrometry (LC-MS) analysis (Fig. 7A). Seven CDCA3-interacting proteins with potential ubiquitin ligase activity were identified (Fig. 7B). By intersecting these proteins with previously reported MYC-interacting proteins [30], four candidates were found to associate with both CDCA3 and MYC (Fig. 7C). Among these, TRIM28 was prioritized due to its established role in reducing ubiquitin-mediated degradation of oncogenic proteins such as PD-L1, TRIM24 and YTHDC1 [31–33], as well as its involvement in BLCA progression via hTERT transcriptional activation [34]. This prompted further investigation into whether TRIM28 mediates CDCA3’s regulatory effect on MYC.

To investigate the interactions among CDCA3, MYC, and TRIM28, Co-IP assays were performed in 293T cells, confirming that TRIM28 associates with both CDCA3 and MYC (Fig. 7D & E). Furthermore, Co-IP assays conducted in T24 BLCA cells revealed endogenous interactions among TRIM28, CDCA3, and MYC (Fig. 7F). Silencing TRIM28 reduced MYC protein levels without affecting its mRNA levels (Fig. 7G, H), while TRIM28 overexpression increased MYC protein expression (Fig. 7I). Additionally, TRIM28 knockdown accelerated MYC degradation, which was rescued by MG-132 treatment (Fig. 7J; Supplementary Fig. 7A). Binding assays revealed that TRIM28 associates with both termini of MYC, with stronger affinity for the N-terminus (Fig. 6K), and also binds to the N-terminal domain of CDCA3 (Fig. 7L). Ubiquitination assays demonstrated that TRIM28 overexpression reduces MYC ubiquitination (Fig. 7M), while TRIM28 silencing increases it (Supplementary Fig. 7B). Notably, CDCA3 overexpression enhanced the TRIM28-MYC interaction (Fig. 7N). Notably, CDCA3 overexpression mitigated the increased ubiquitination of MYC caused by TRIM28 silencing, restoring MYC protein levels (Fig. 7O; Supplementary Fig. 7C). Functional assays confirmed that co-overexpression of CDCA3 and silencing of TRIM28 restored the proliferative capacity of BLCA cells (Supplementary Fig. 7D, E). The above results conclusively confirm that CDCA3 recruits TRIM28 to stabilize MYC by reducing its ubiquitin-mediated degradation, thereby promoting BLCA cell proliferation.

### MYC promotes *CDCA3* transcription

Previous studies have demonstrated transcriptional upregulation of CDCA3 in BLCA cells [20, 21]. Given the central role of MYC as a transcription factor, it is hypothesized that MYC also regulates *CDCA3* transcription. Analysis of the TIMER 2.0 database reveals that *CDCA3* is positively related to *MYC* (Fig. 8A). Examination of ChIP-seq data from neuroblastoma cells (GSE13829553) identifies a significant MYC binding peak within the *CDCA3* promoter region (Fig. 8B) [35], further supported by JASPAR database predictions of a potential E-box sequence in this region (Fig. 8C, D). RNA-seq data from public repositories (GSE87693) indicate that MYC silencing significantly reduces *CDCA3* mRNA levels (Fig. 8E) [36]. Consistent with these observations, our qRT-PCR and WB results confirm that MYC silencing decreases CDCA3 expression, while MYC overexpression enhances it (Fig. 8F-I & Supplementary Fig. 8). Additionally, a luciferase reporter assay demonstrates that MYC overexpression increases *CDCA3* promoter activity (Fig. 8J). Collectively, these results establish that MYC regulates CDCA3 expression at the transcriptional level.

## Discussion

The persistent activation of proliferative signaling is a hallmark of neoplastic cells [7]. Coupled with dysregulated cell cycle control, this continuous proliferation arises from aberrant expression of cell cycle regulatory proteins, which are indispensable for initiating and sustaining proliferation signals in malignant cells. Tumor cell metabolism exhibits phase-dependent variations synchronized with the cell cycle. During the G1 phase, cells predominantly utilize the tricarboxylic acid cycle to oxidize nutrients, whereas glycolysis becomes more prominent in the S phase, a process largely governed by the cell cycle regulator SKP2. Overexpression of SKP2 skews cellular metabolism towards glycolysis, thereby driving tumorigenesis [37]. This study reveals the critical role of another cell cycle regulator, CDCA3, in modulating glycolysis in BLCA, underscoring the significance of the CDCA3-MYC feedback loop in promoting BLCA progression.

MYC, a key oncogenic transcription factor, plays a central role in the metabolic reprogramming of cancer cells by activating the expression of various metabolic enzymes. The regulatory impact of MYC on ENO1 transcription is tightly linked to MYC protein stability [38, 39]. Ubiquitin-mediated degradation of MYC diminishes its binding to the ENO1 promoter, thereby reducing ENO1 transcription and suppressing glycolysis in tumor cells [29]. MYC, a short-lived protein, is predominantly degraded through the ubiquitin-proteasome pathway. This process involves key phosphorylation events, including ERK-mediated phosphorylation at Ser62 and GSK-3β-mediated phosphorylation at Thr58, which mark MYC for recognition and degradation by the E3 ubiquitin ligase FBXW7 via the 26 S proteasome [40, 41]. In BLCA, DNA polymerase POLD1 and the ubiquitin-specific protease USP43 competitively bind to MYC, preventing its interaction with FBXW7 and thereby stabilizing MYC protein levels. Furthermore, MYC transcriptionally activates POLD1 and USP43, forming reciprocal positive feedback loops (POLD1-MYC and USP43-MYC) that promote BLCA progression [42, 43]. This investigation identifies CDCA3 as a critical modulator of MYC stability, demonstrating that CDCA3 interacts with MYC and mitigates its ubiquitination and degradation by recruiting TRIM28. Moreover, MYC directly enhances CDCA3 transcription, establishing a reciprocal feedback loop. This interaction amplifies ENO1 transcription, thereby facilitating glycolysis and contributing to BLCA progression.

Two noteworthy phenomena emerged from this study. The first pertains to the subcellular localization of CDCA3, which is predominantly cytoplasmic [44, 45]. Despite this, our findings reveal that CDCA3 interacts with two proteins primarily located in the nucleus. Furthermore, Gene Ontology (GO) analysis of our LS-MS results indicates that CDCA3-interacting proteins are predominantly nuclear, suggesting that a subset of CDCA3 may translocate to the nucleus to contribute to BLCA progression. The second observation involves TRIM28, which interacts with both the N- and C-termini of MYC. As a transcriptional repressor, TRIM28 is known to silence endogenous retroviruses (ERVs) by binding their 5’UTRs through KRAB-ZNF interactions [46]. Interestingly, in BLCA, TRIM28 functions as a transcriptional activator, directly binding to a mutant hTERT promoter allele to upregulate its expression [34]. The interaction of TRIM28 with the C-terminus of MYC, a region that also interacts with the transcriptional activator MAX, hints at an unexplored role for TRIM28 in modulating MYC transcriptional activity. Further investigation into the roles of CDCA3 and TRIM28 in BLCA could unveil new avenues for targeted therapeutic development.

In conclusion, this study identifies a novel function of CDCA3 in regulating glycolysis. As a cell cycle regulator, CDCA3 interacts with MYC, preventing its degradation by recruiting TRIM28 to inhibit ubiquitination. This interaction enhances the glycolytic pathway mediated by ENO1 and promotes CDCA3 transcription in a positive feedback loop, contributing to BLCA progression (Fig. 9). These findings provide a new perspective on BLCA pathogenesis and offer potential strategies for future diagnostic and therapeutic advancements.

## Electronic supplementary material

Below is the link to the electronic supplementary material.

Supplementary Material 1

Supplementary Material 2

Supplementary Material 3

Supplementary Material 4

## Data Availability

Data from the GSE13507, GSE32894 and Cancer Cell Line Encyclopedia (CCLE) cohorts are publicly available and were used in this study. Proteins that interact with GFP-CDCA3 was provided in the data repository as supplementary information.

## Abbreviations

BLCA: Bladder Cancer
CDCA3: Cell Division Cycle Associated-3
ENO1: α-Enolase
TRIM28: Tripartite Motif Containing 28
GEM: Gemcitabine
WB: Western Blot
Co-IP: Co-immunoprecipitation
GEO: Gene Expression Omnibus
GSEA: Gene Set Enrichment Analysis
AUC: Area Under the Curve
ccRCC: clear cell renal cell carcinoma
