# Supplementary material for: CDCA3-MYC positive feedback loop promotes bladder cancer progression via ENO1-mediated glycolysis
Source: J Exp Clin Cancer Res. 2025 Feb 20;44:63. doi: 10.1186/s13046-025-03325-7 (PMC11841255; doi:10.1186/s13046-025-03325-7)

Figure 2C

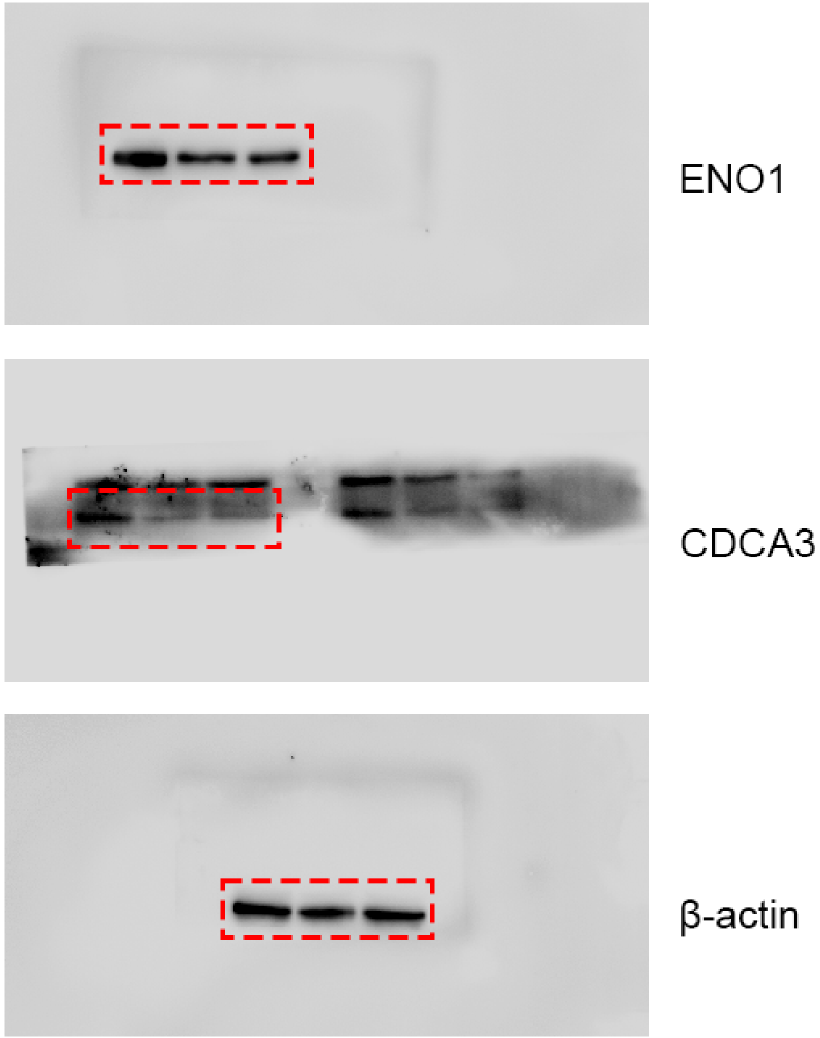

Figure 5B

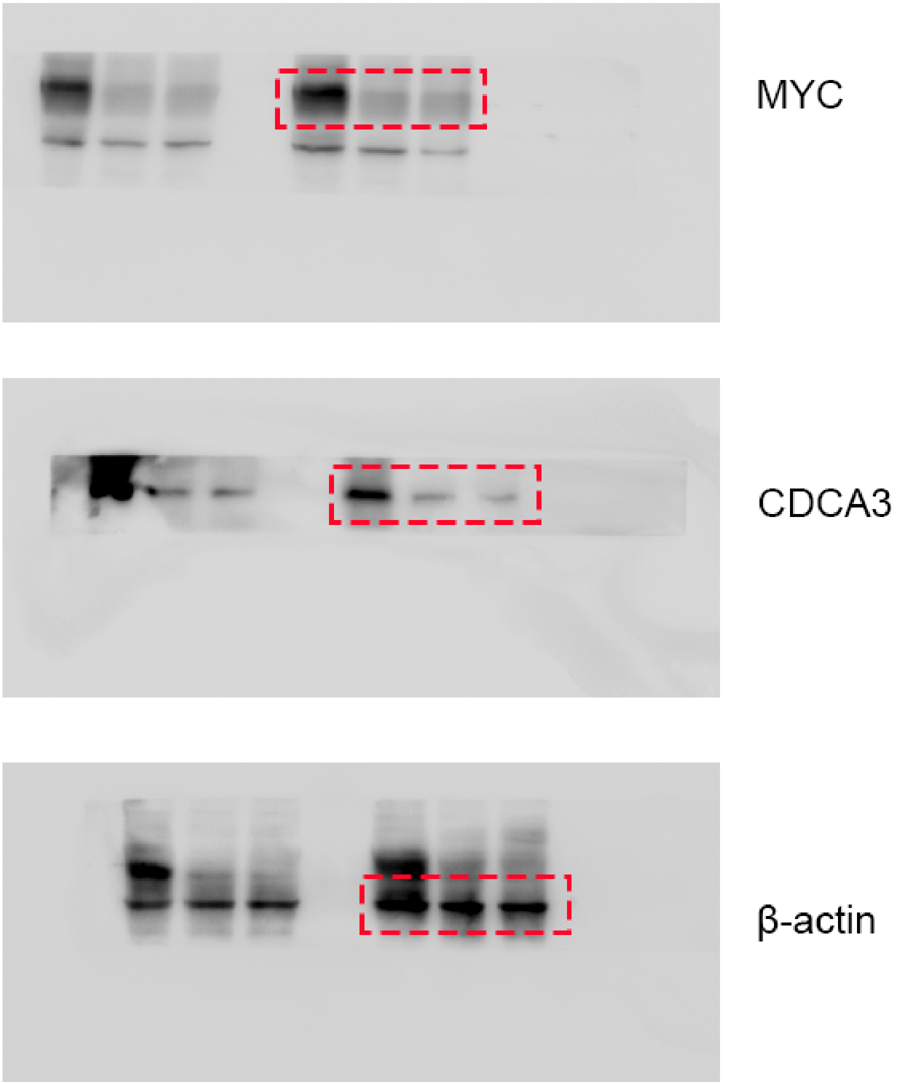

Figure 5C

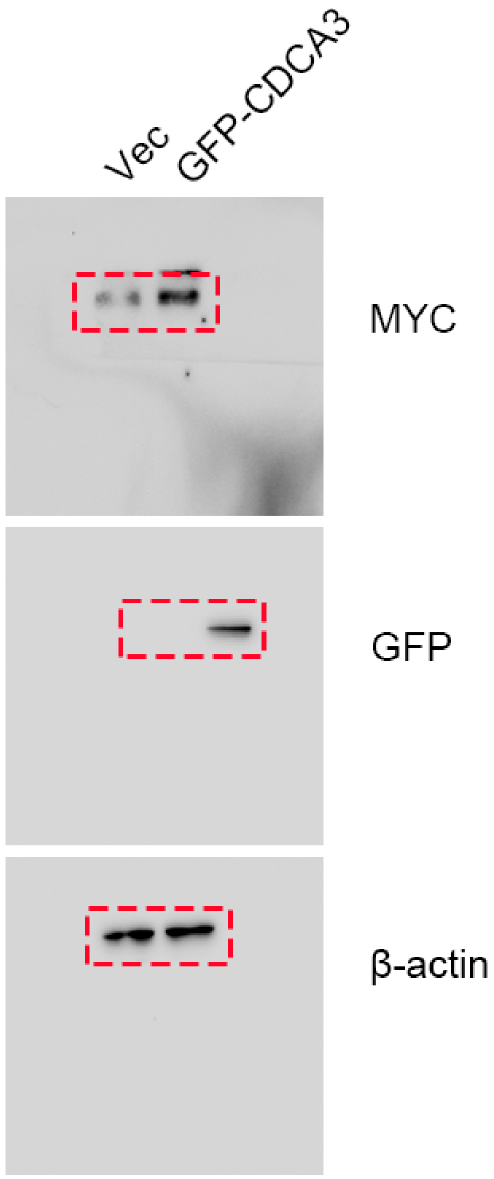

Figure 5D

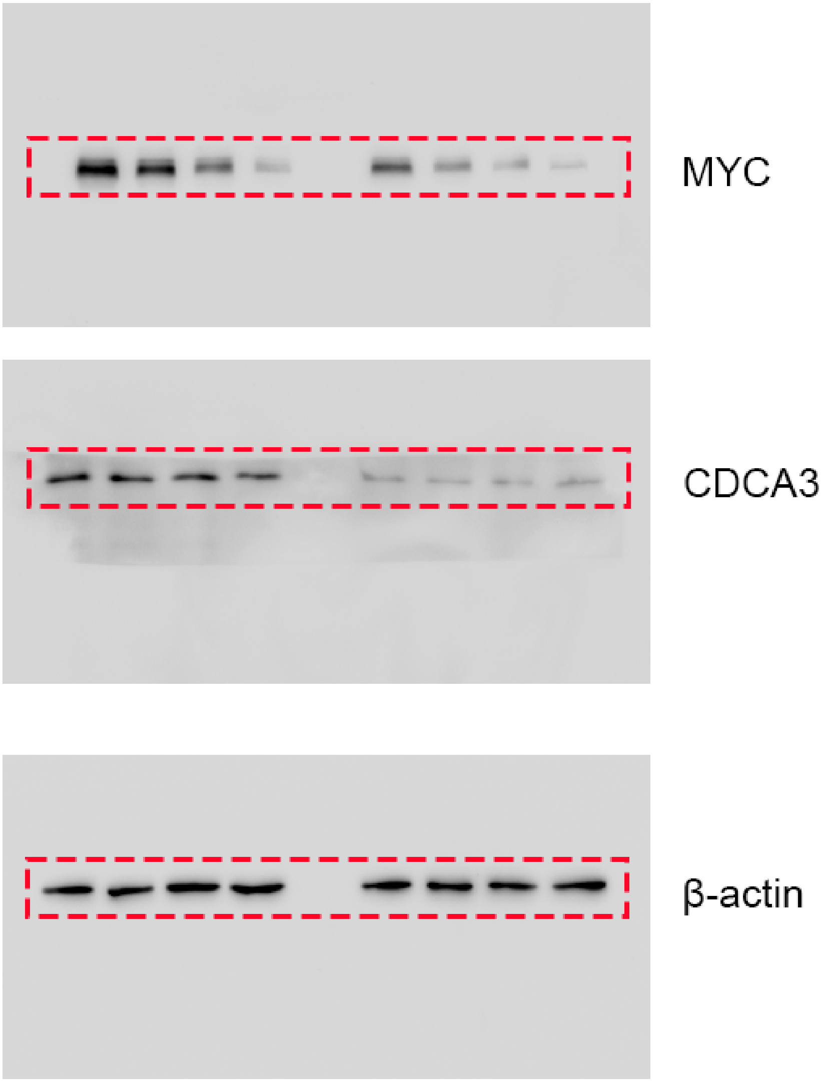

Figure 5E

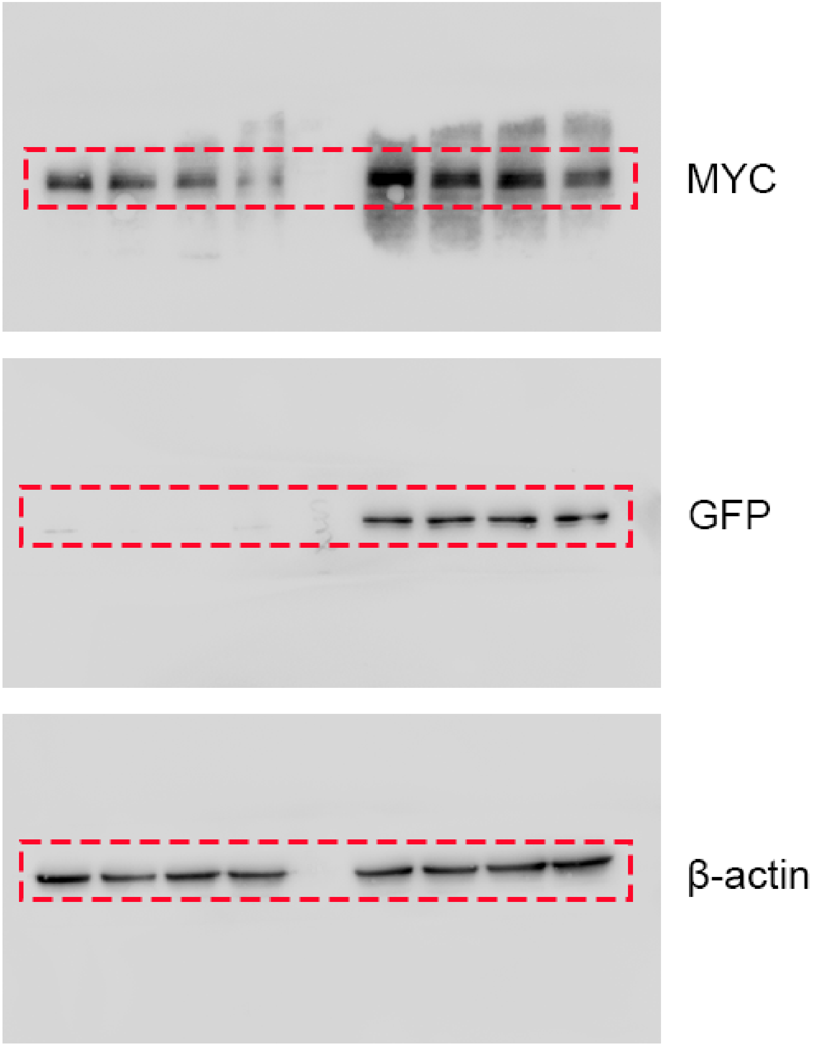

Figure 5F left panel

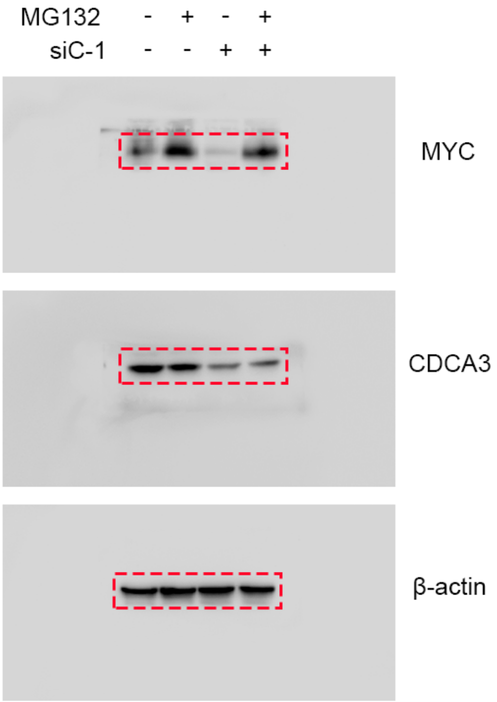

Figure 5F right panel

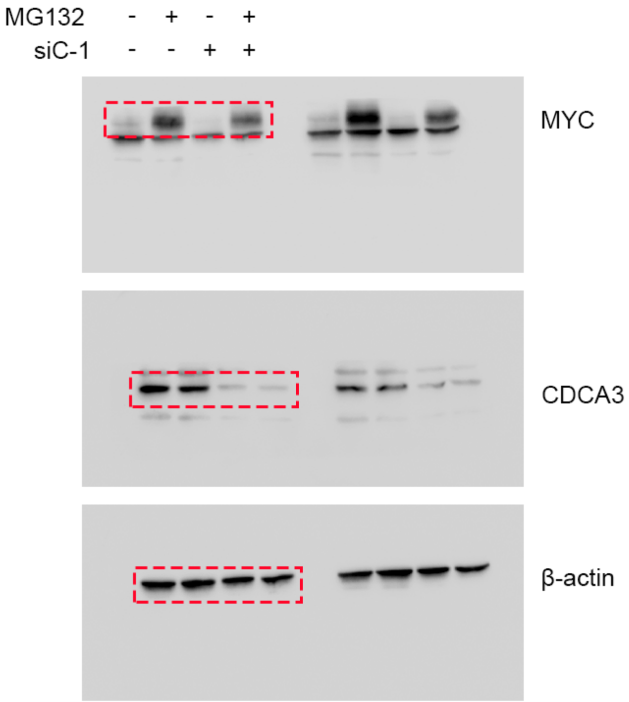

Figure 5G

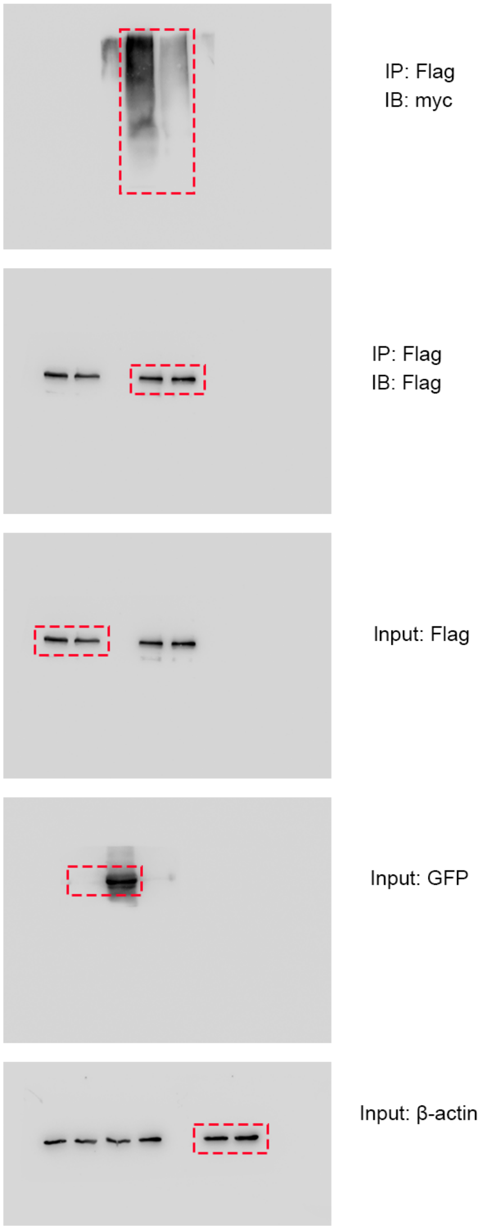
IP: Flag
IB: myc
IP: Flag
IB: Flag
Input: Flag
Input: GFP
Input: β-actin

Figure 6B left panel

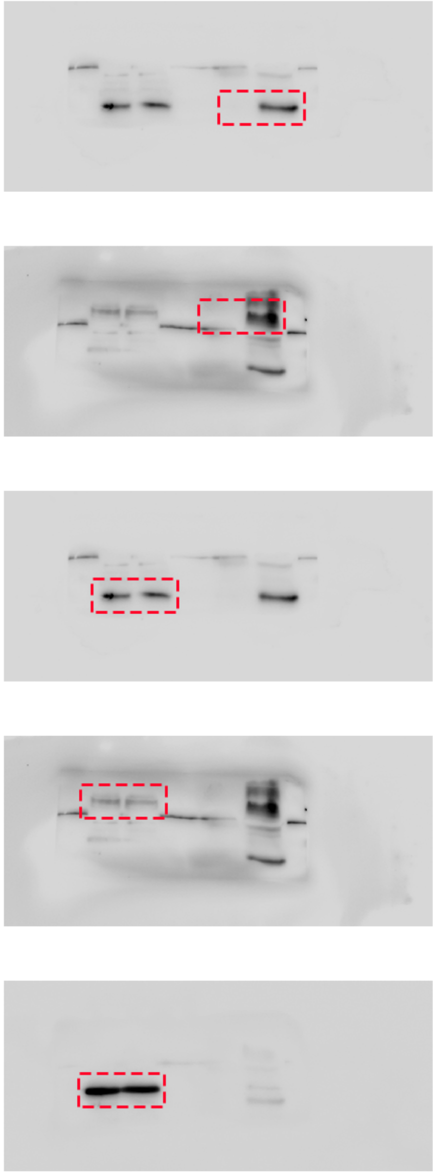

Figure 6B right panel

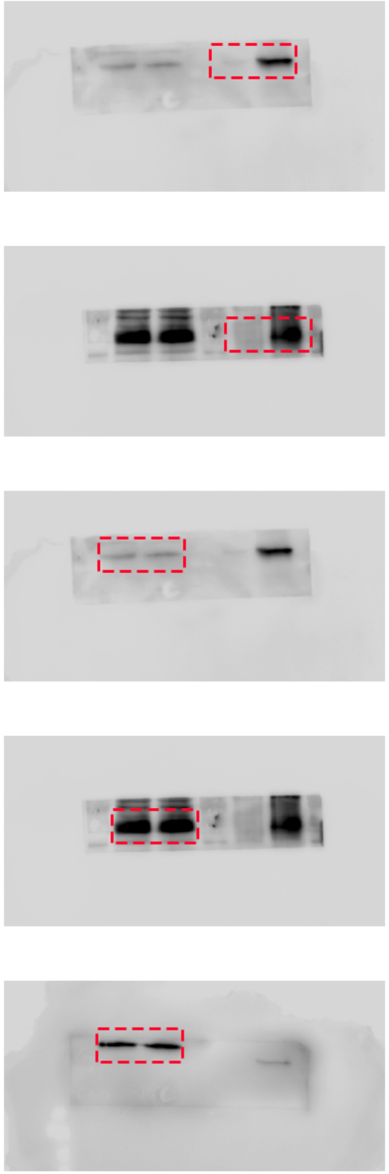

Figure 6C left panel

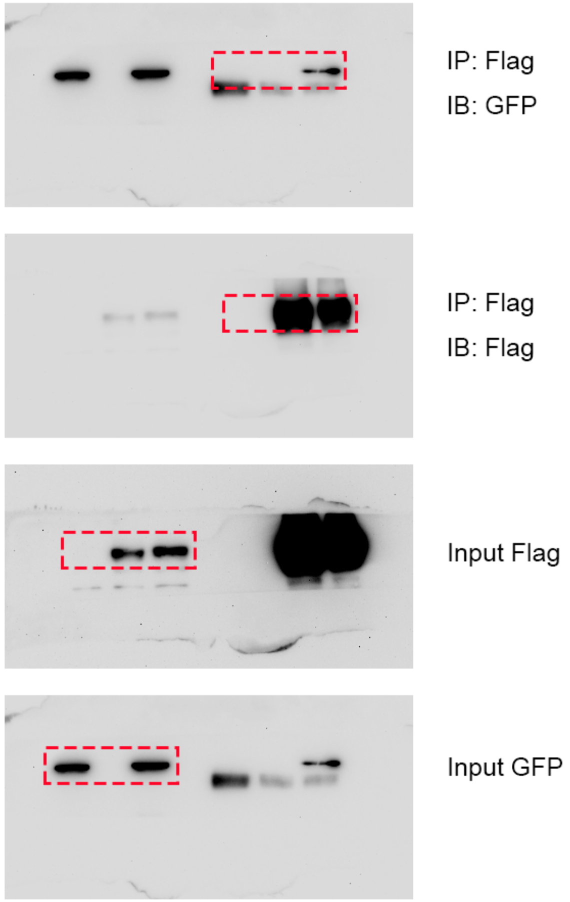

Figure 6C right panel

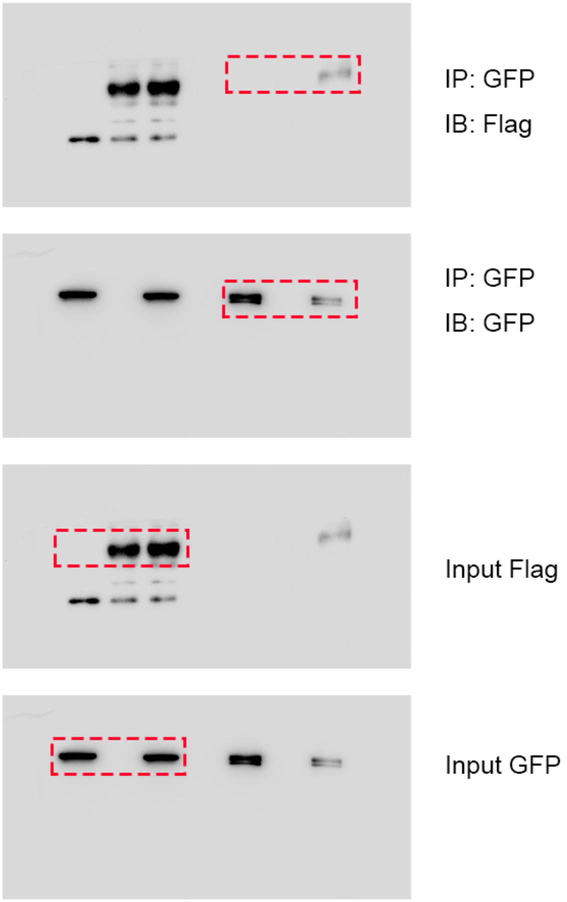

Figure 6D

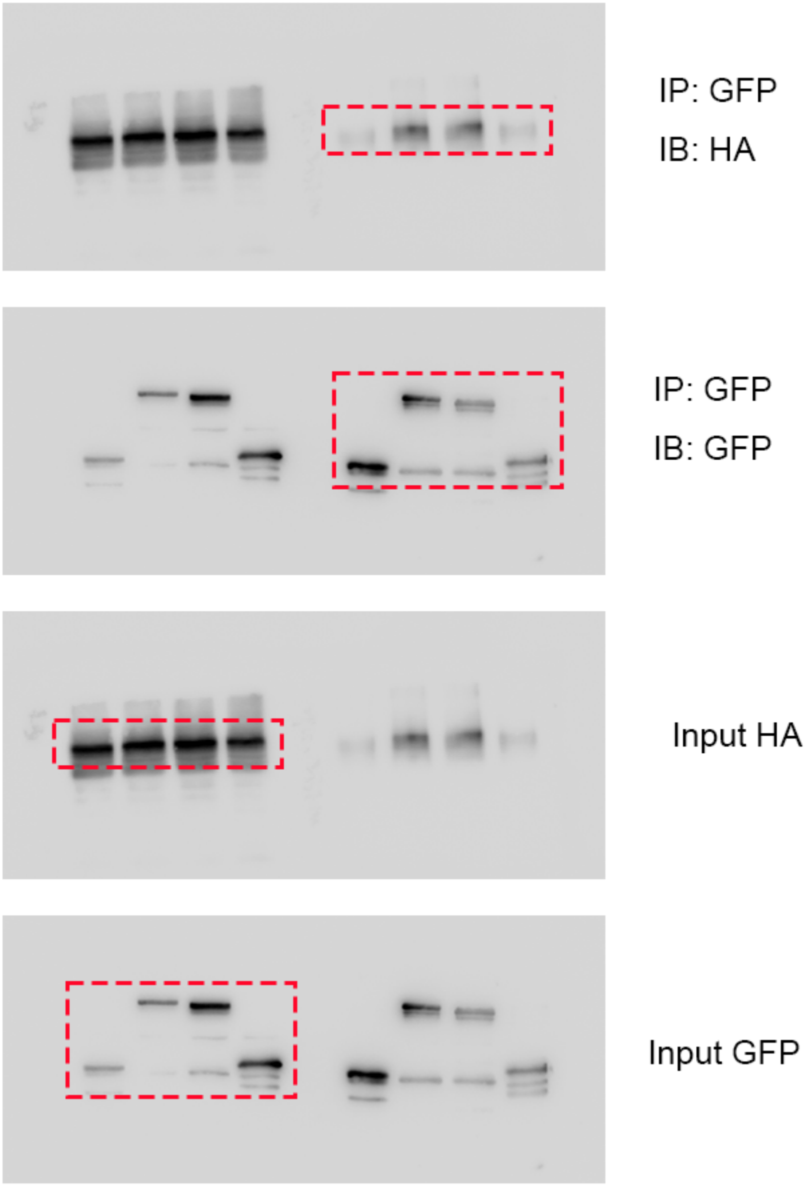

Figure 6E

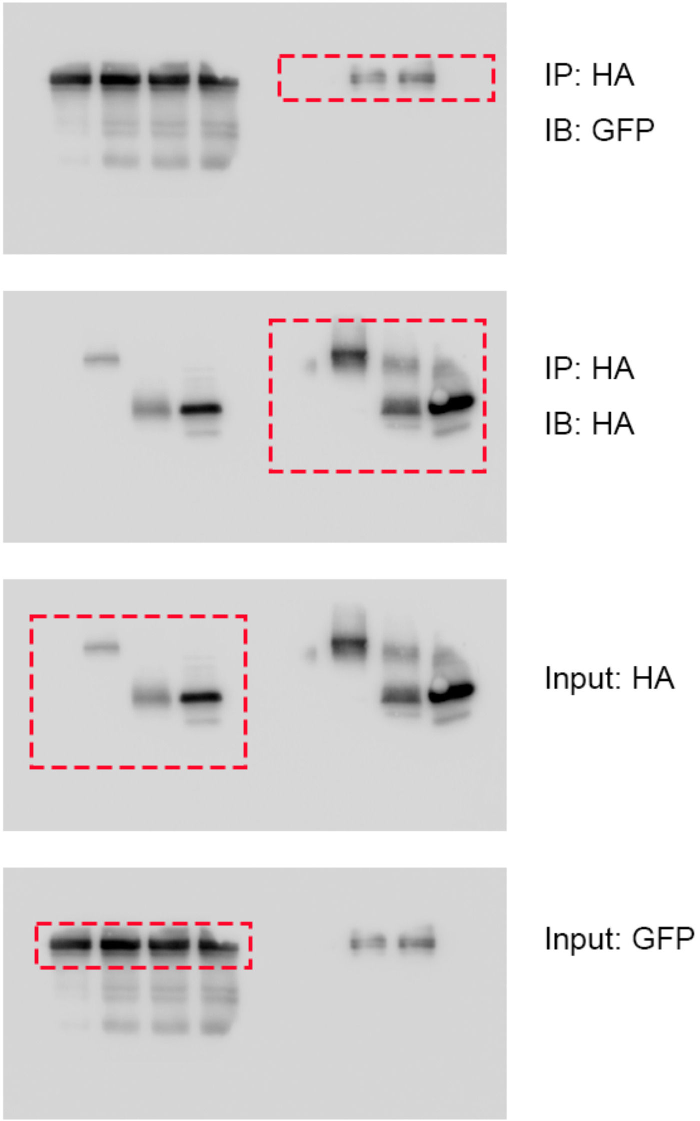

Figure 6F

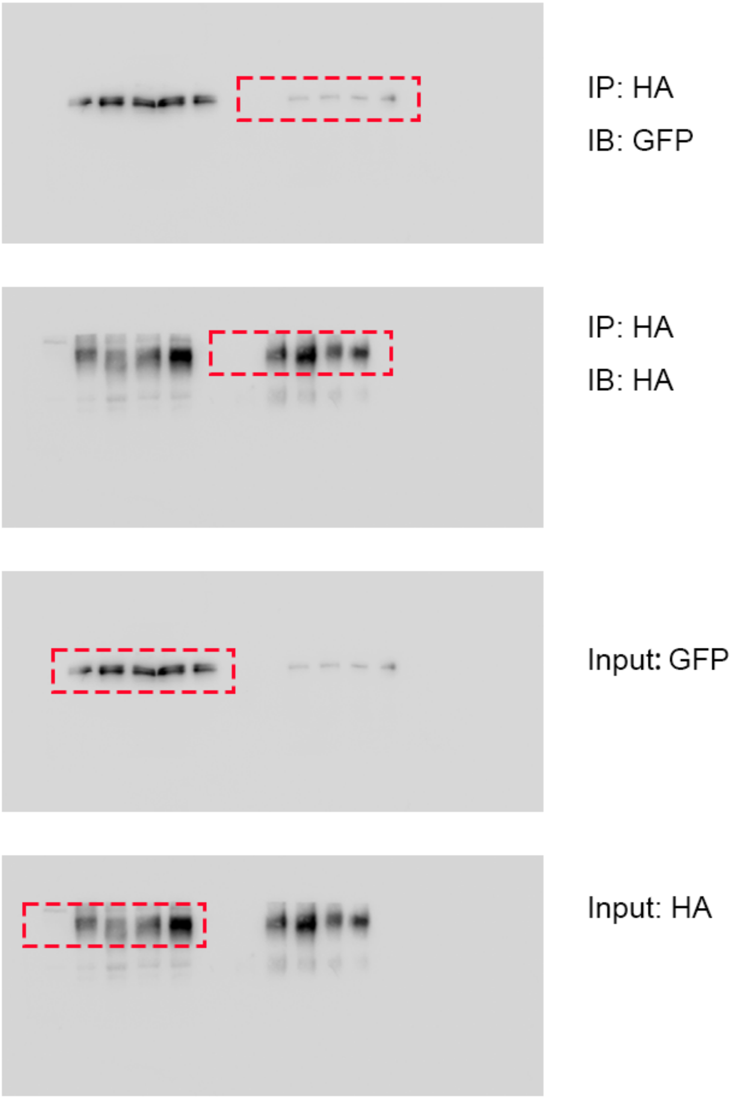

Figure 7D

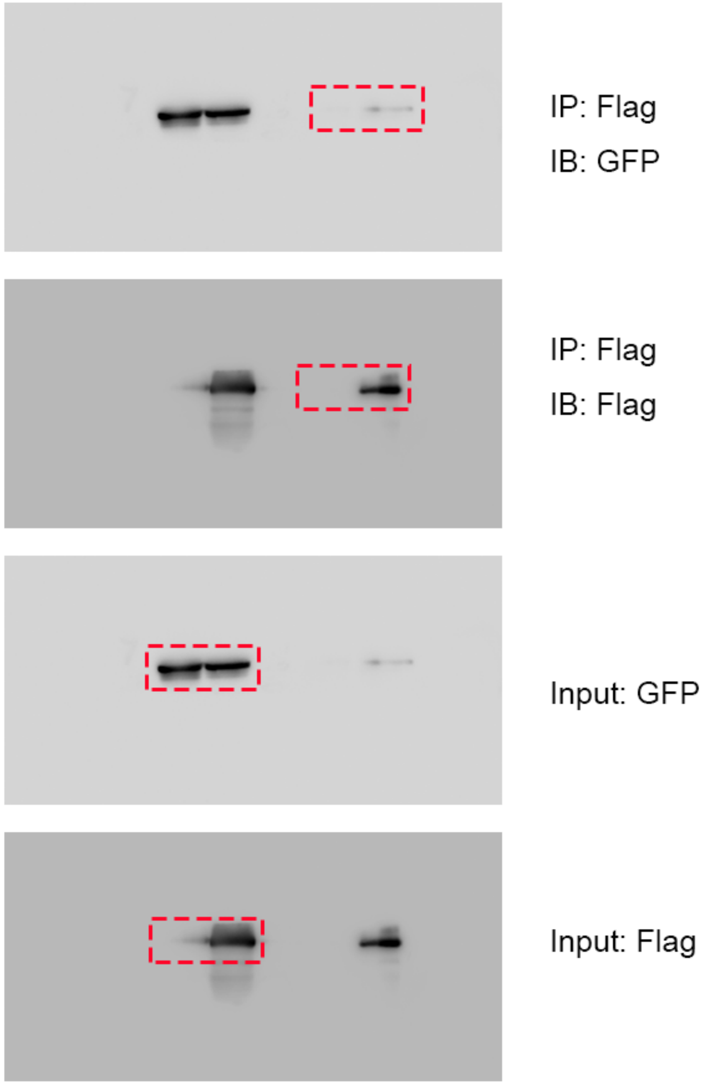

Figure 7E

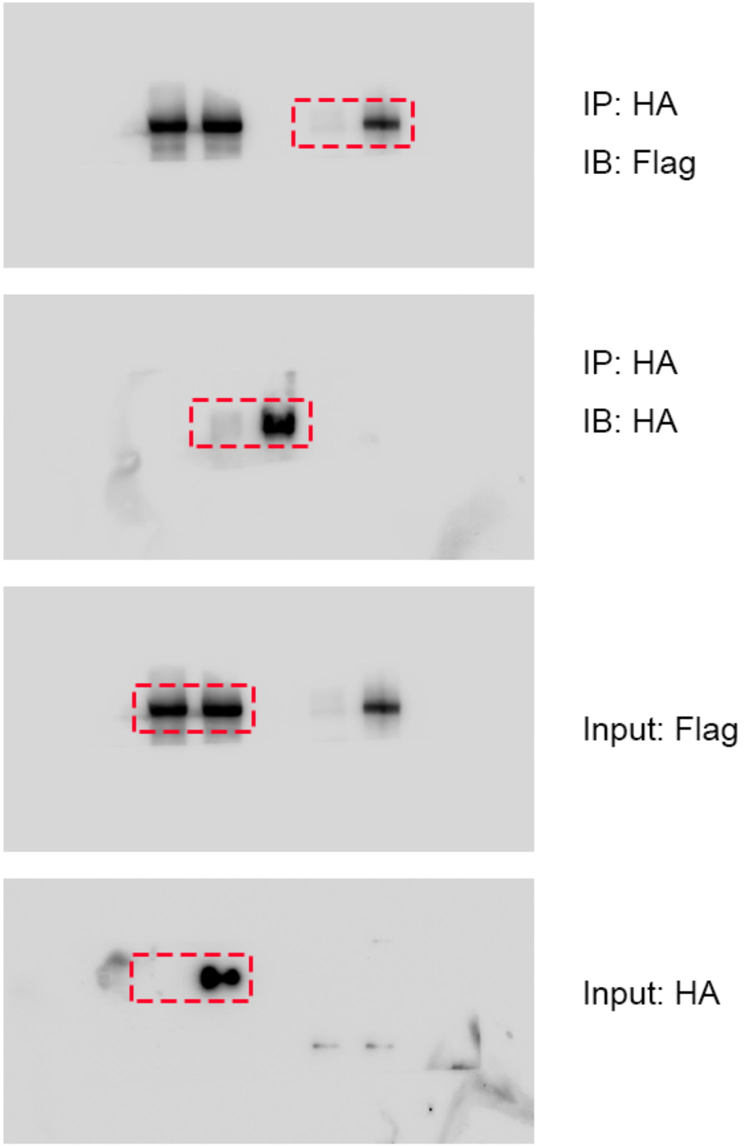

Figure 7F

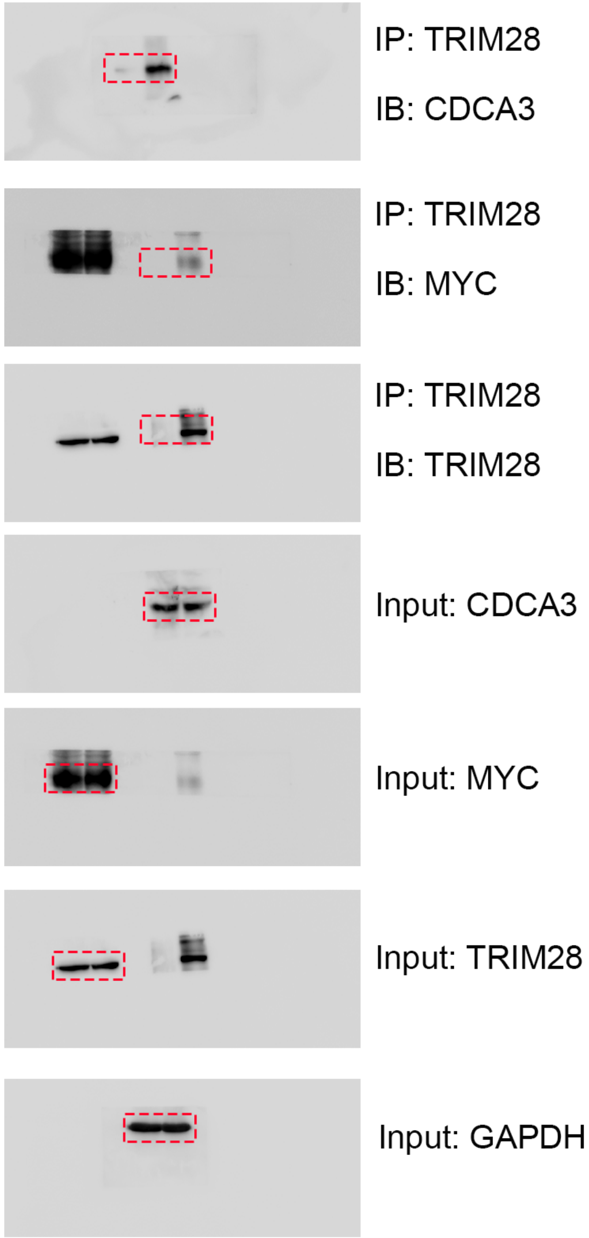
IP: TRIM28
IB: CDCA3
IP: TRIM28
IB: MYC
IP: TRIM28
IB: TRIM28
Input: CDCA3
Input: MYC
Input: TRIM28
Input: GAPDH

Figure 7H

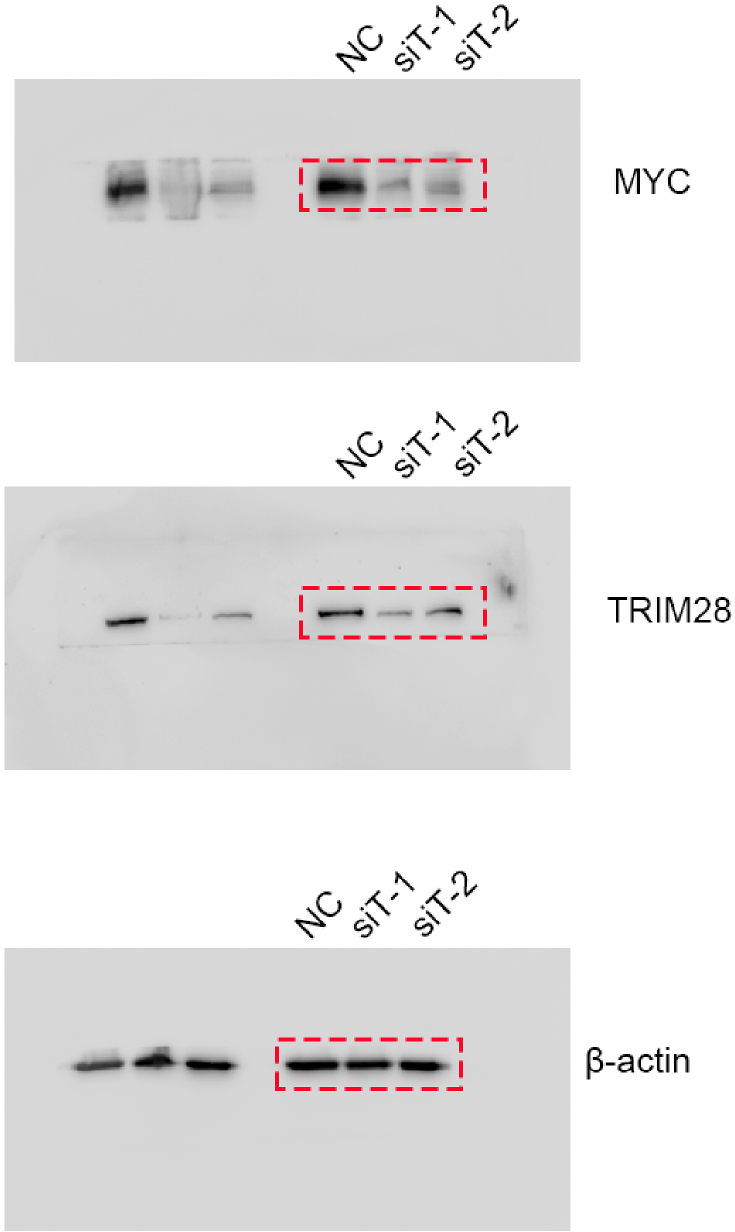

Figure 7I

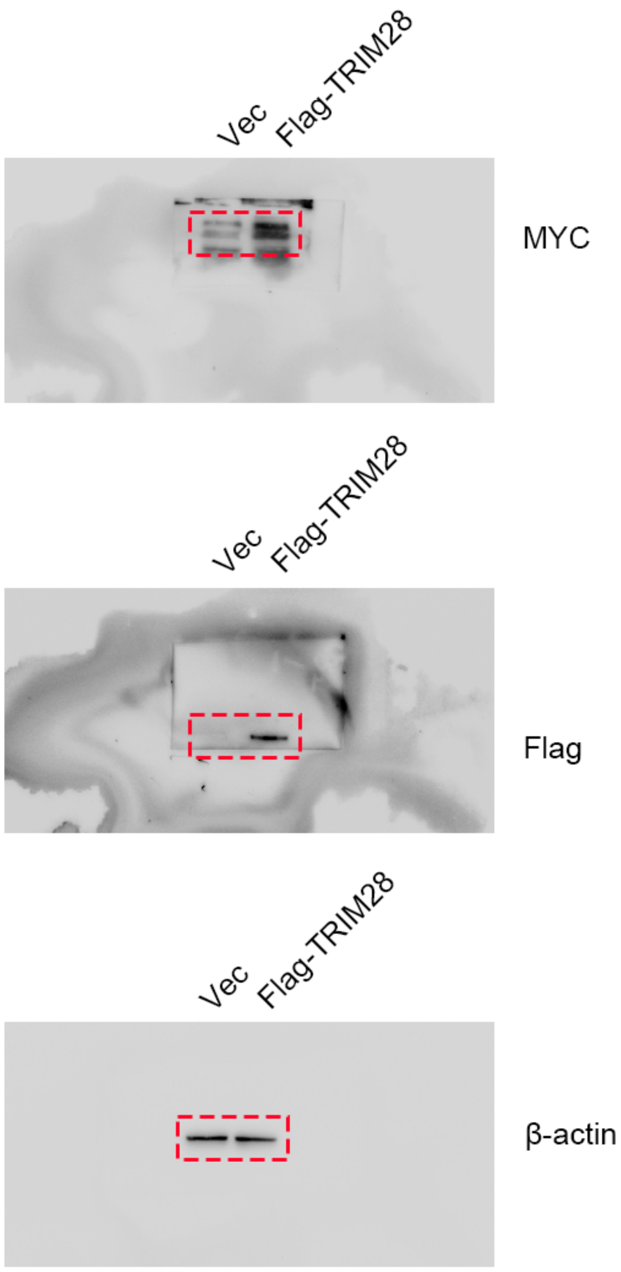
Vec
Flag-TRIM28
MYC
Vec
Flag-TRIM28
Flag
Vec
Flag-TRIM28
β-actin

Figure 7J

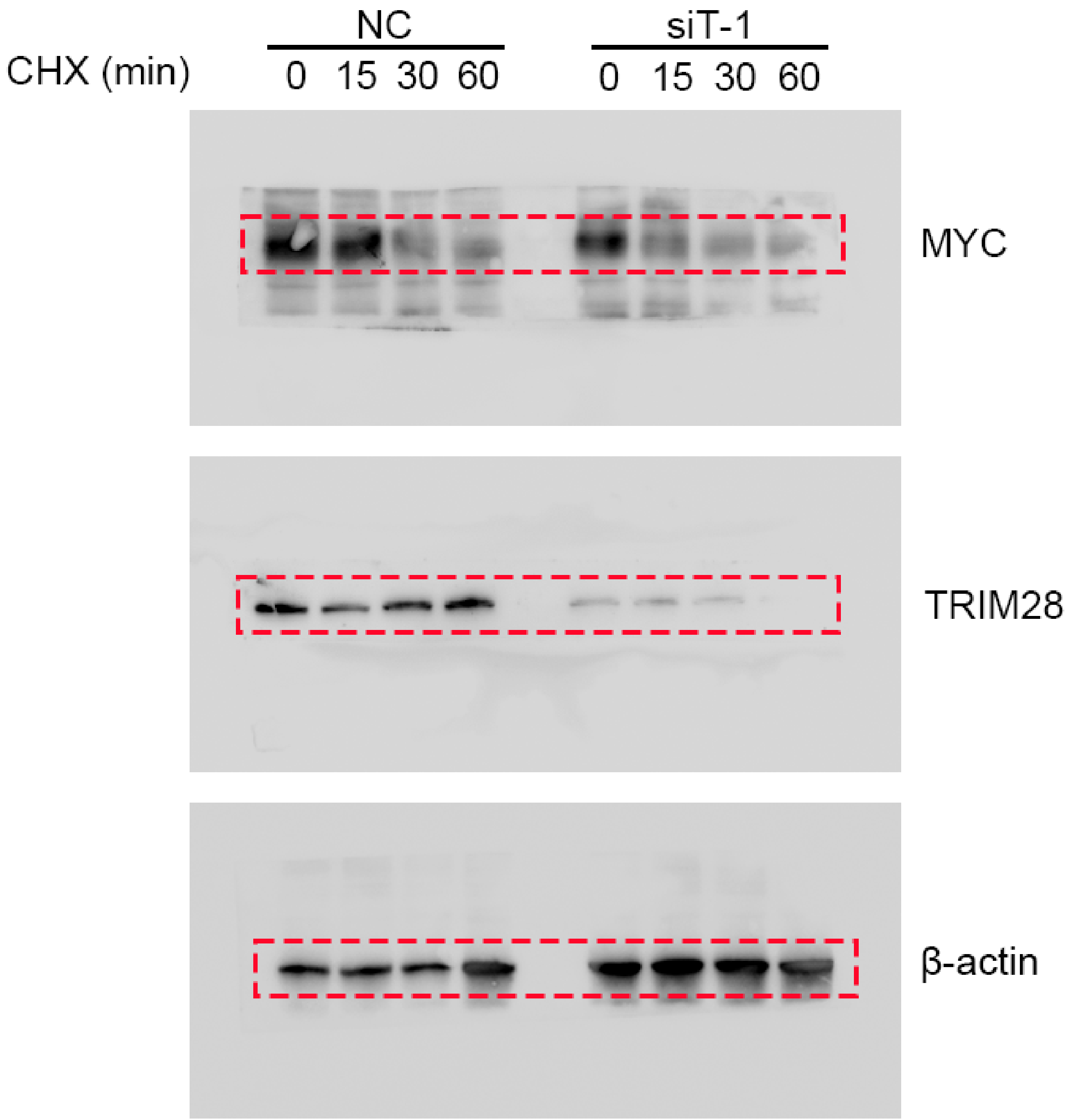

Figure 7K

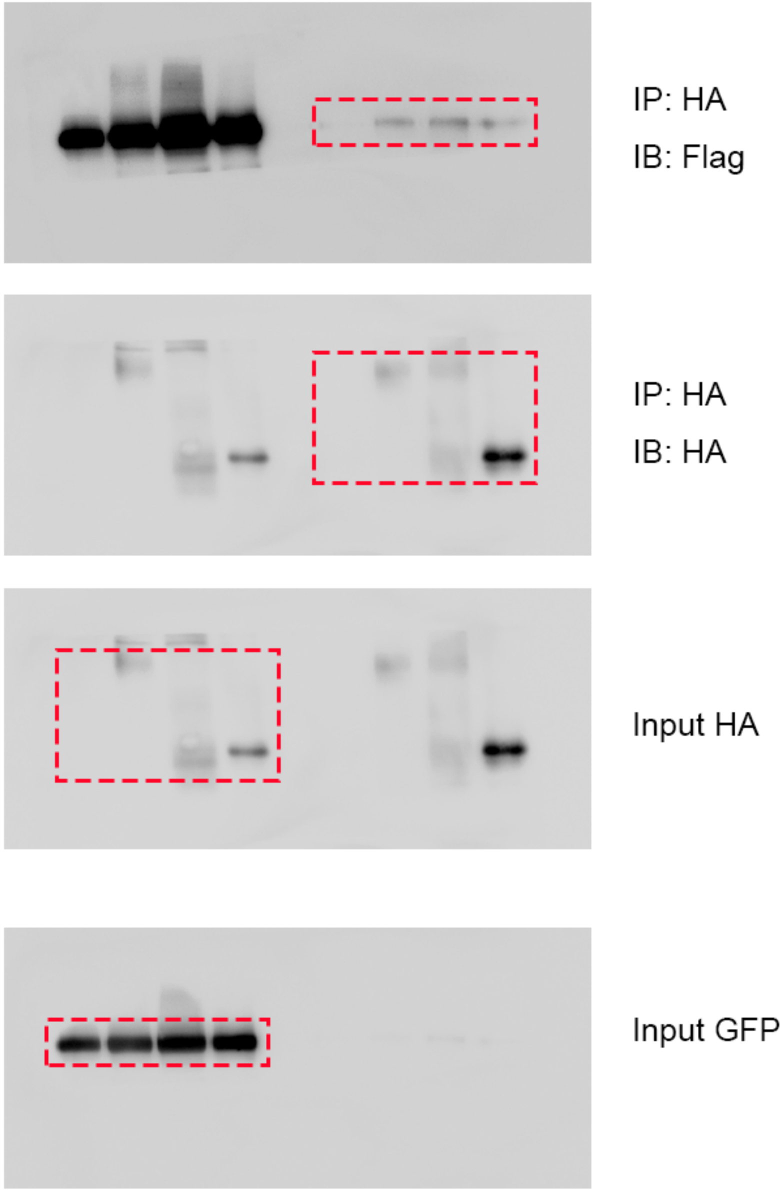

Figure 7L

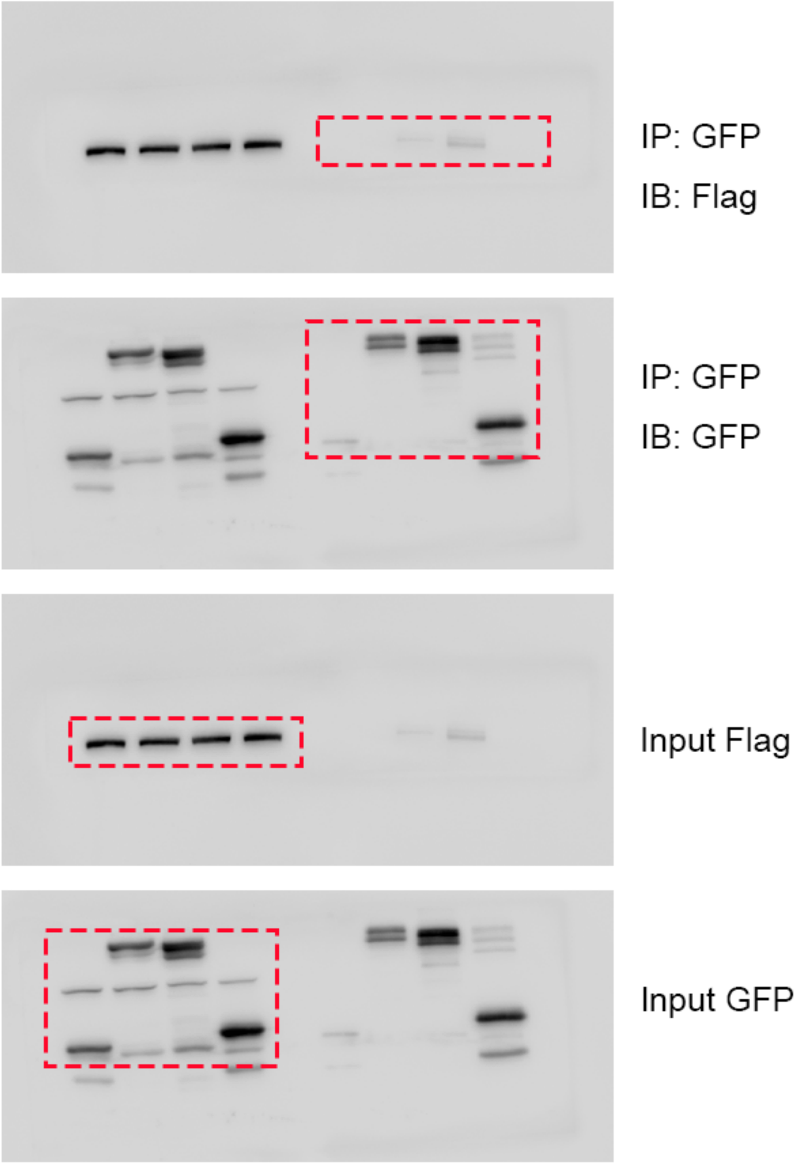

Figure 7M

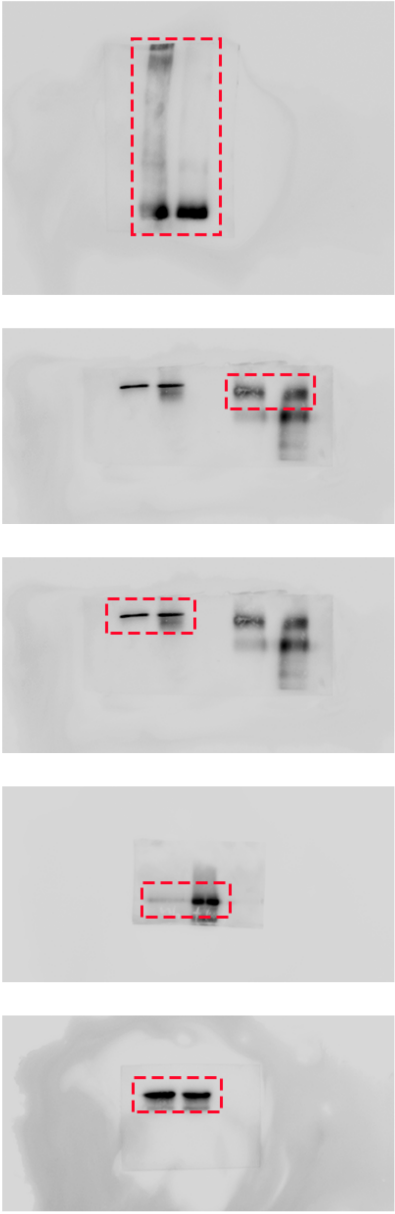

IP: HA
IB: myc

IP: HA
IB: HA

Input HA

Input Flag

Input β-actin

Figure 7N

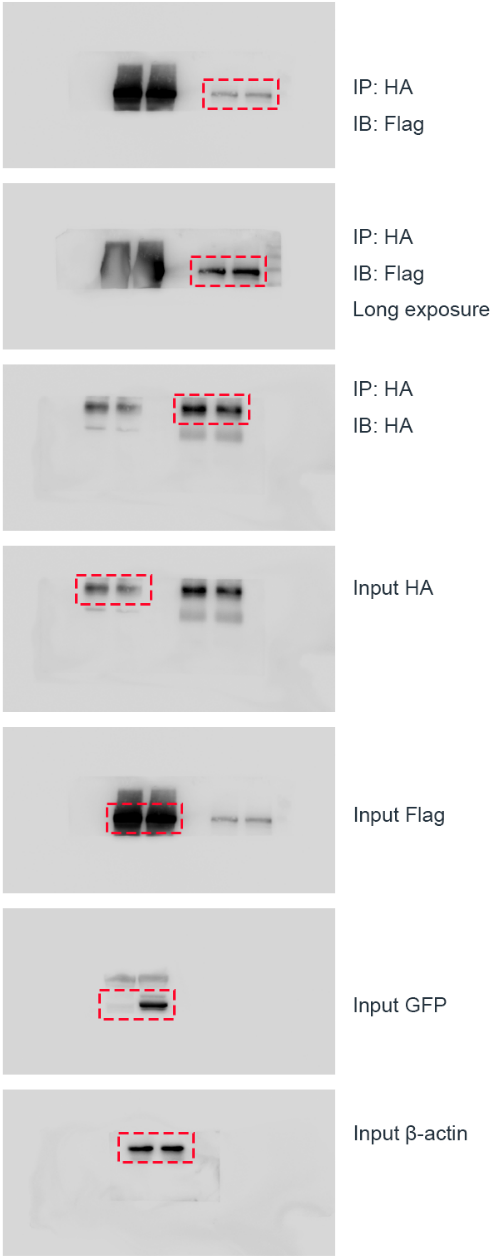
IP: HA
IB: Flag
IP: HA
IB: Flag
Long exposure
IP: HA
IB: HA
Input HA
Input Flag
Input GFP
Input β-actin

Figure 7O

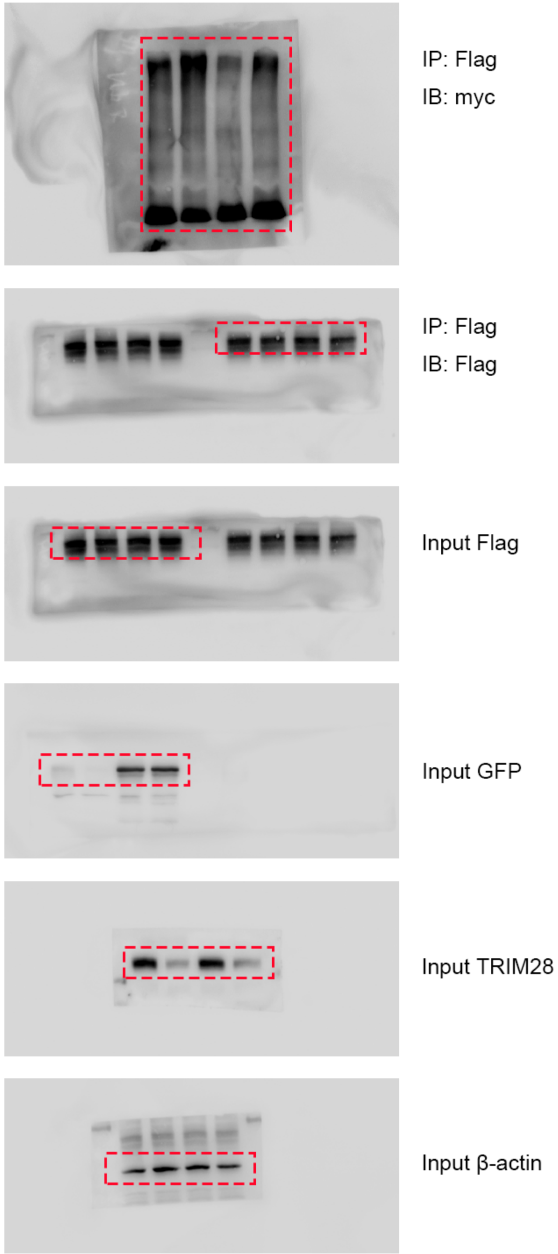
IP: Flag
IB: myc
IP: Flag
IB: Flag
Input Flag
Input GFP
Input TRIM28
Input β-actin

Figure 8G

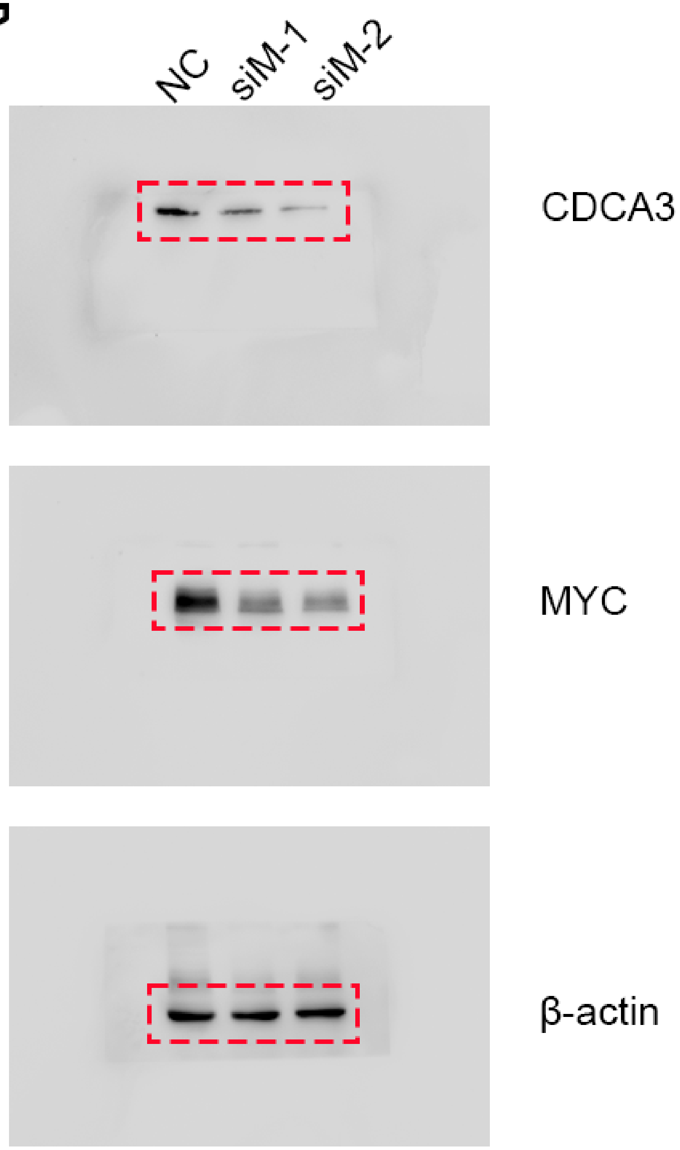

Figure 8I

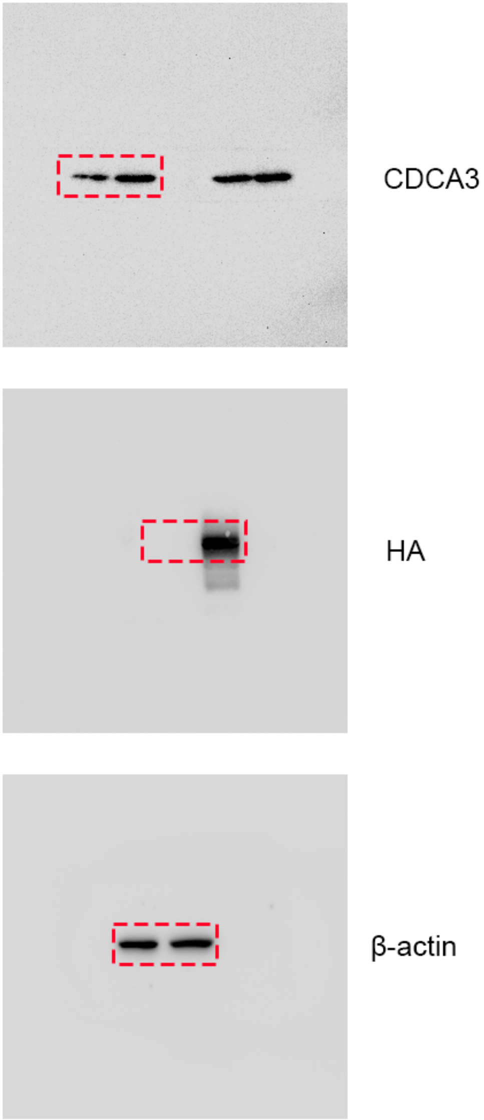

Supplementary Figure 1A

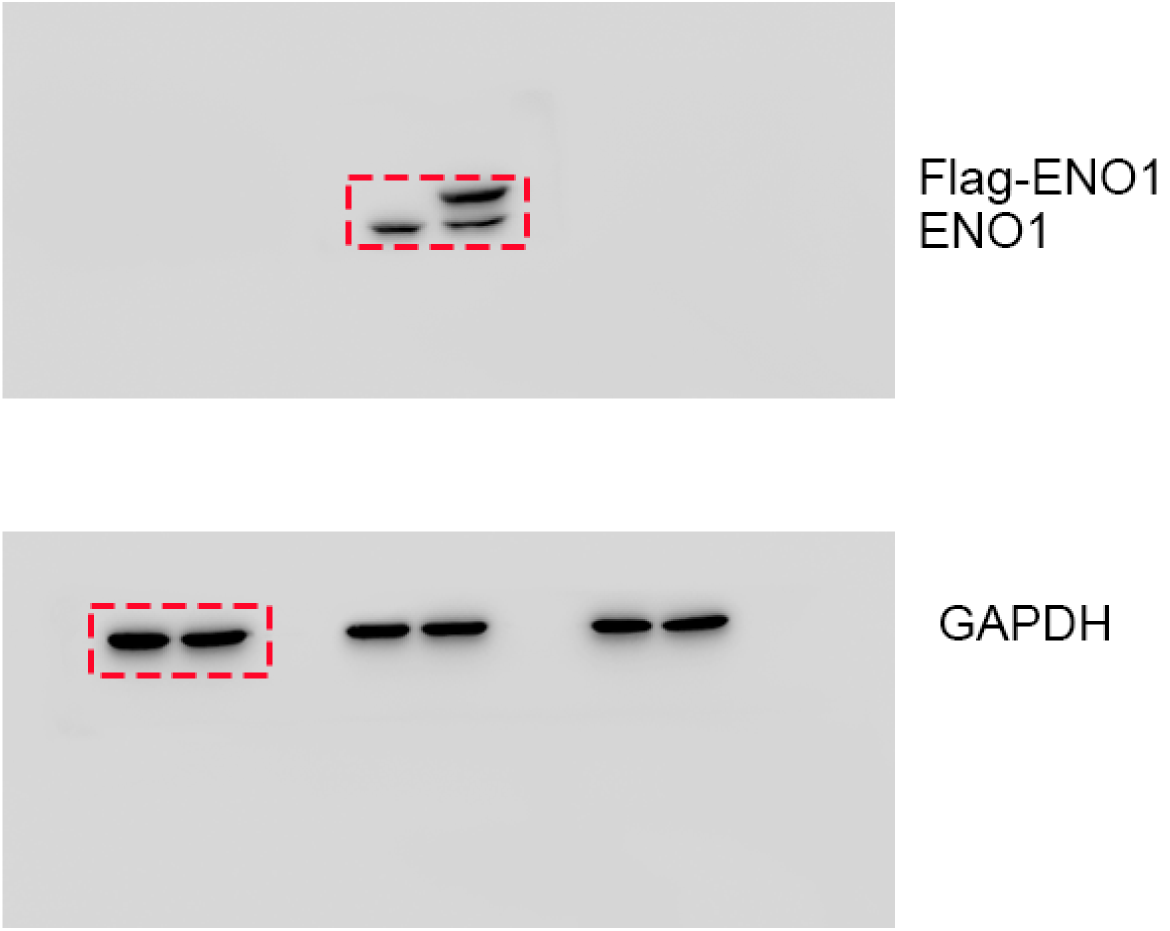

Supplementary Figure 1C

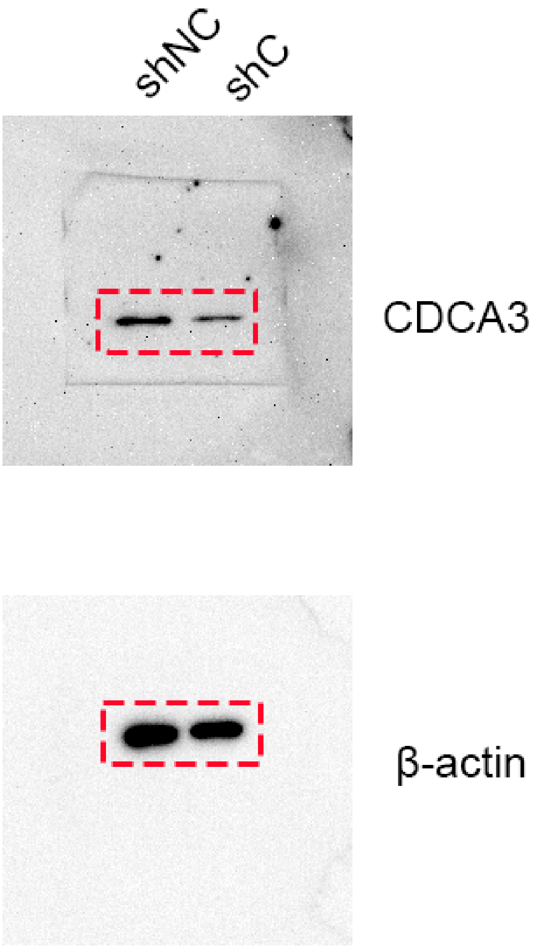

Supplementary Figure 1E

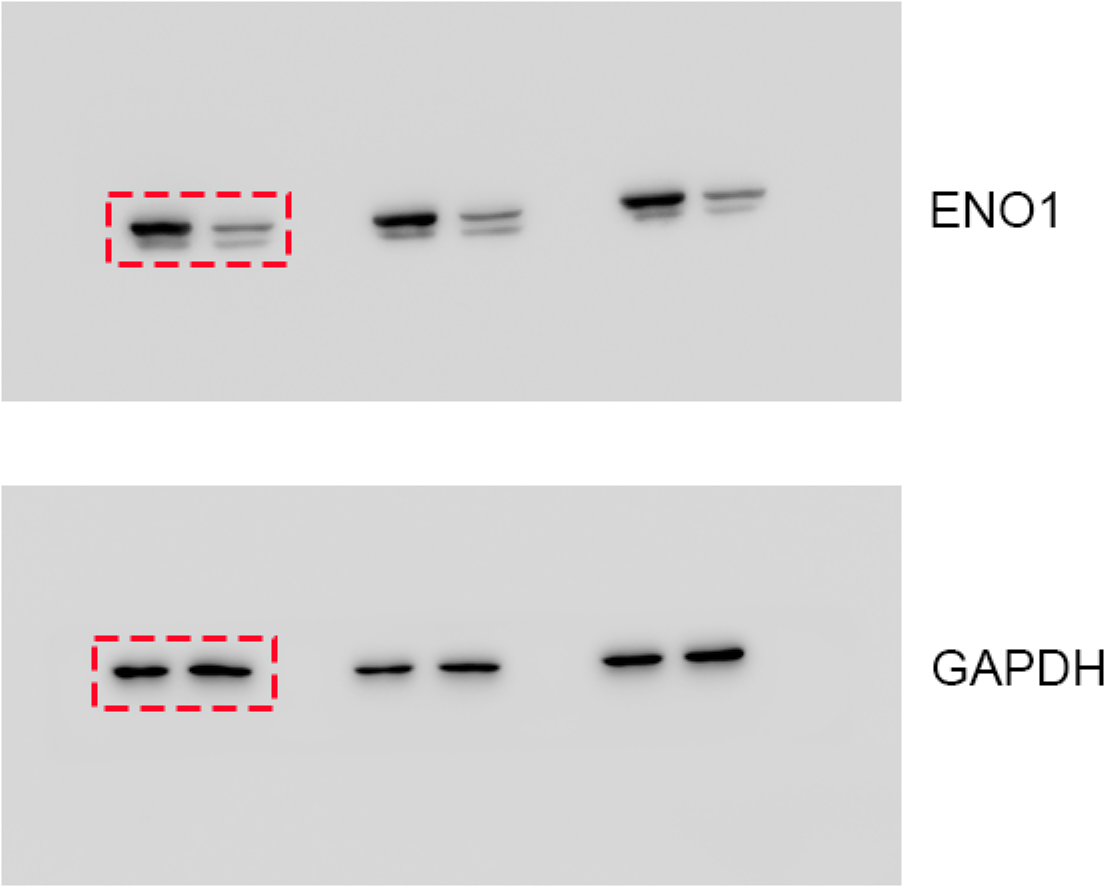

Supplementary Figure 6B

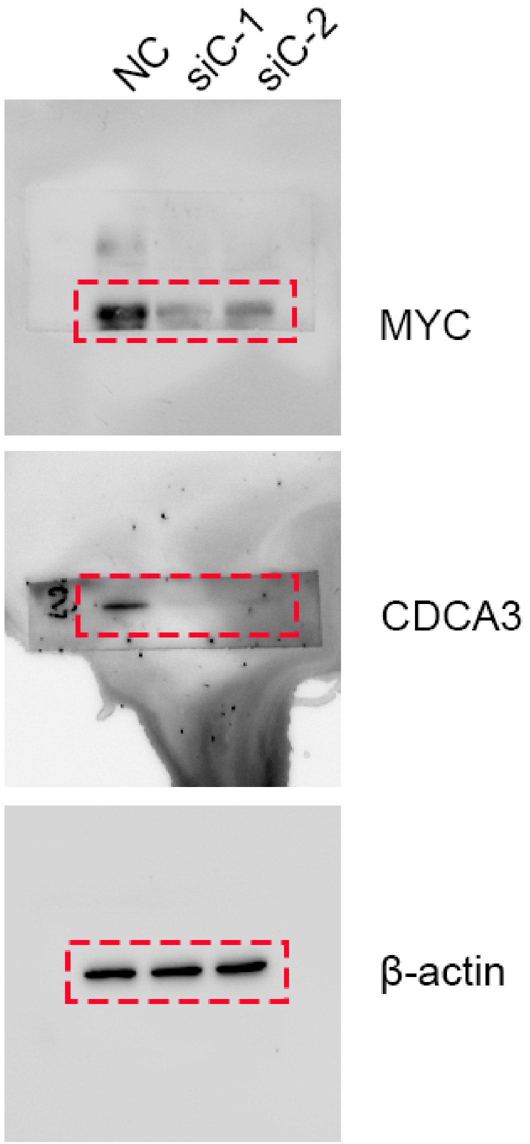

Supplementary Figure 5C

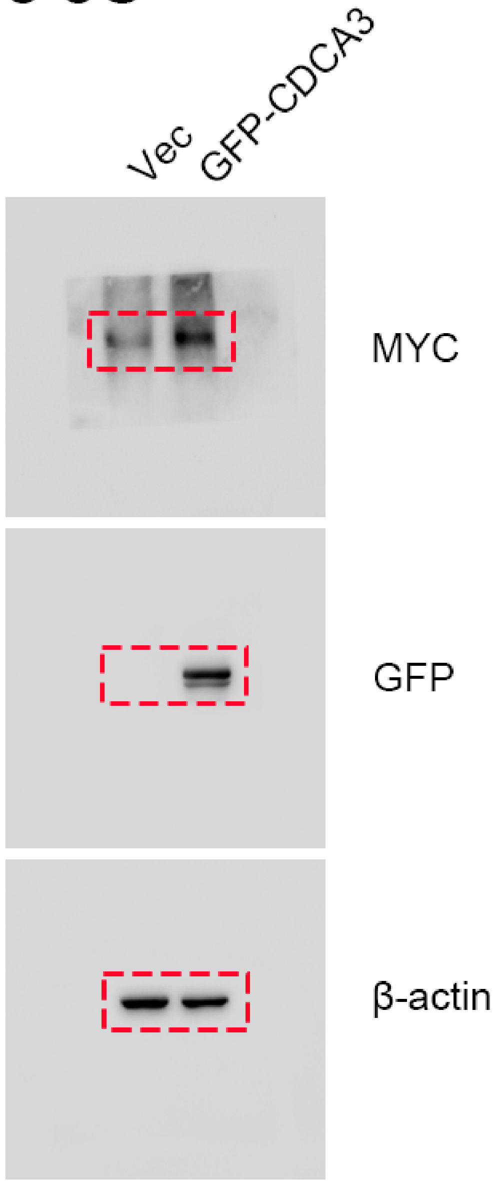

Supplementary Figure 6D

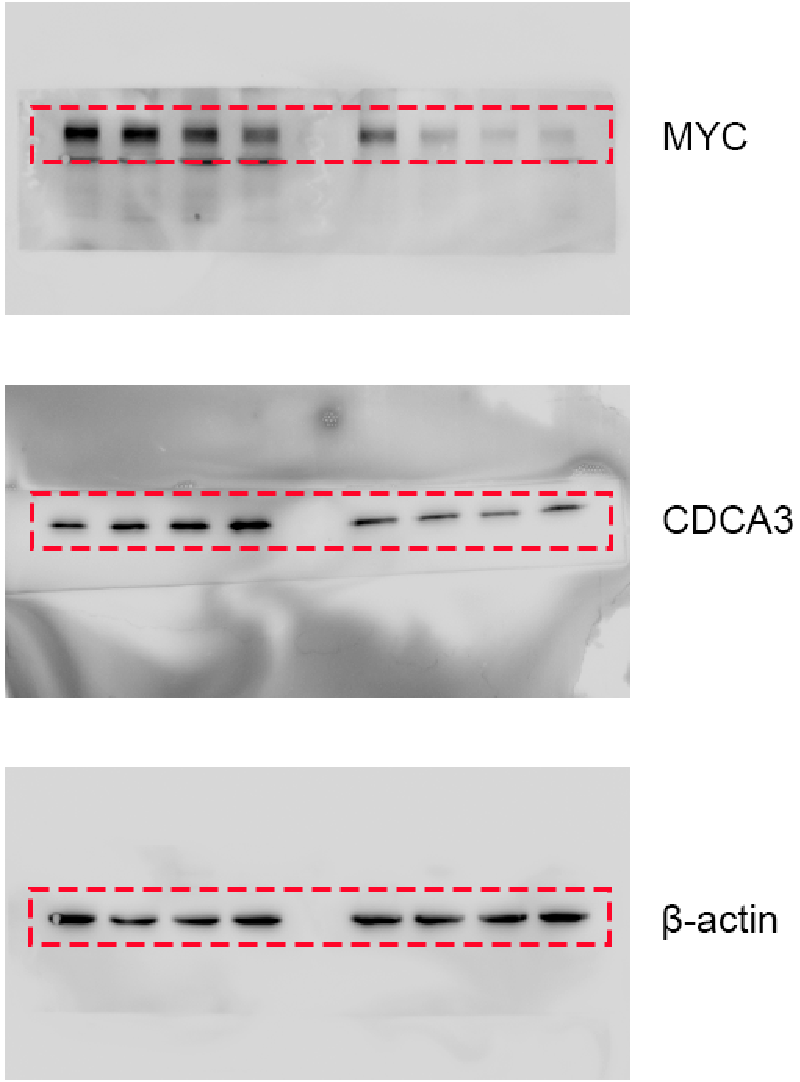

Supplementary Figure 6E

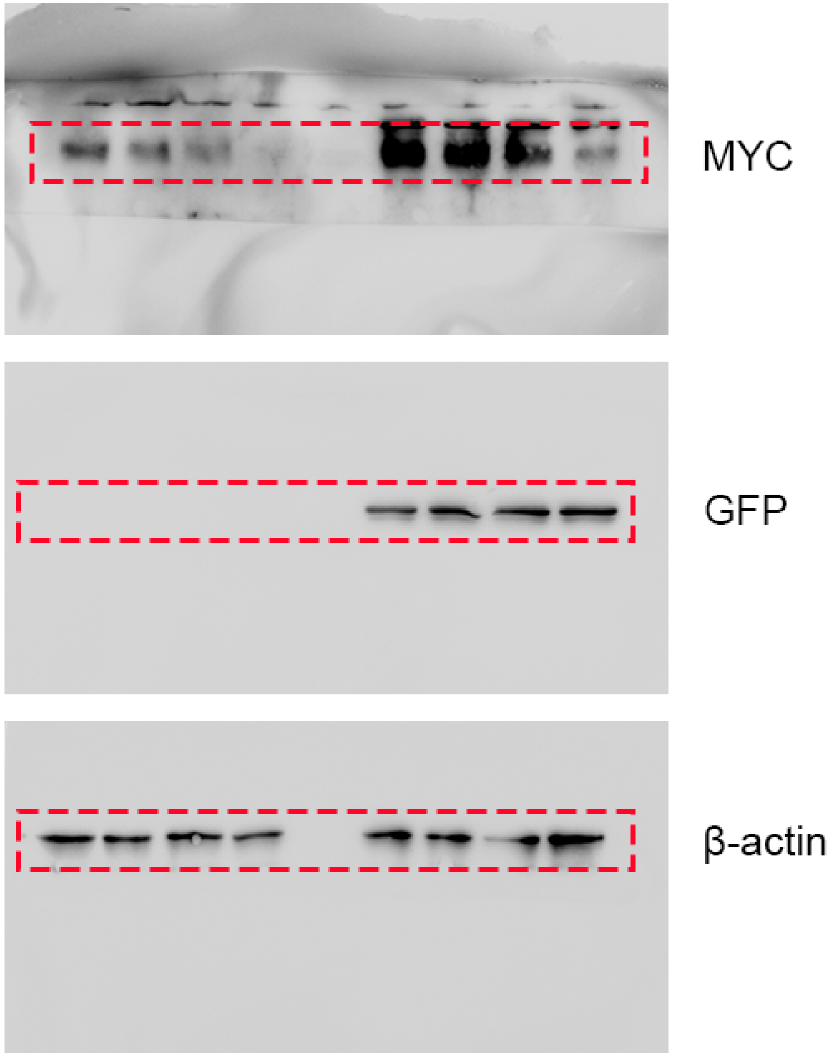

Supplementary Figure 6F left panel

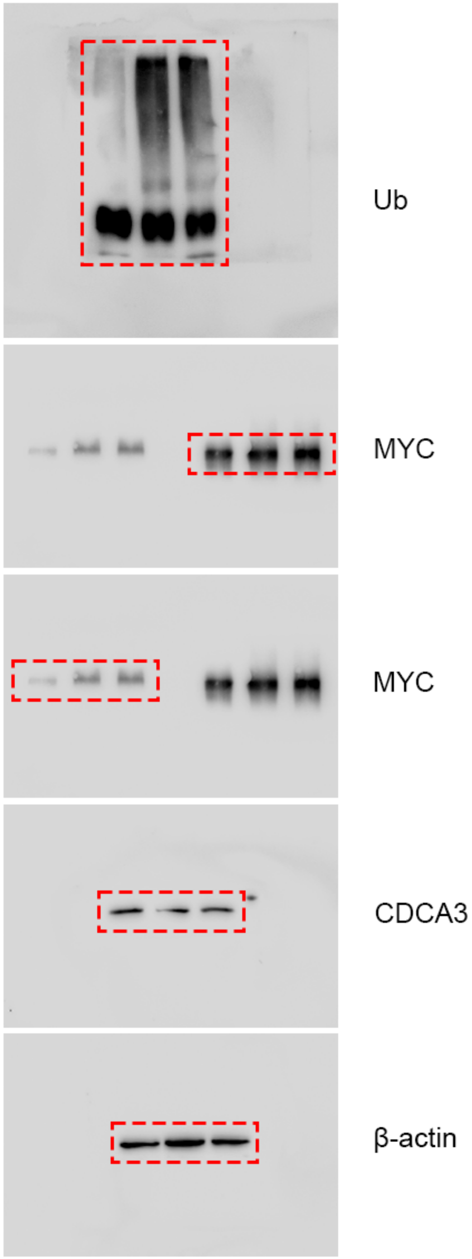

Supplementary Figure 6F right panel

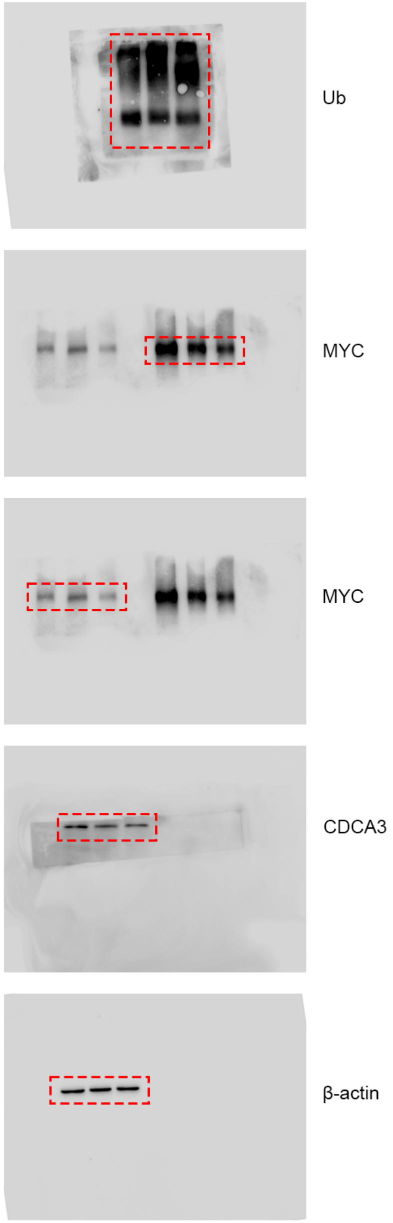

Supplementary Figure 7A

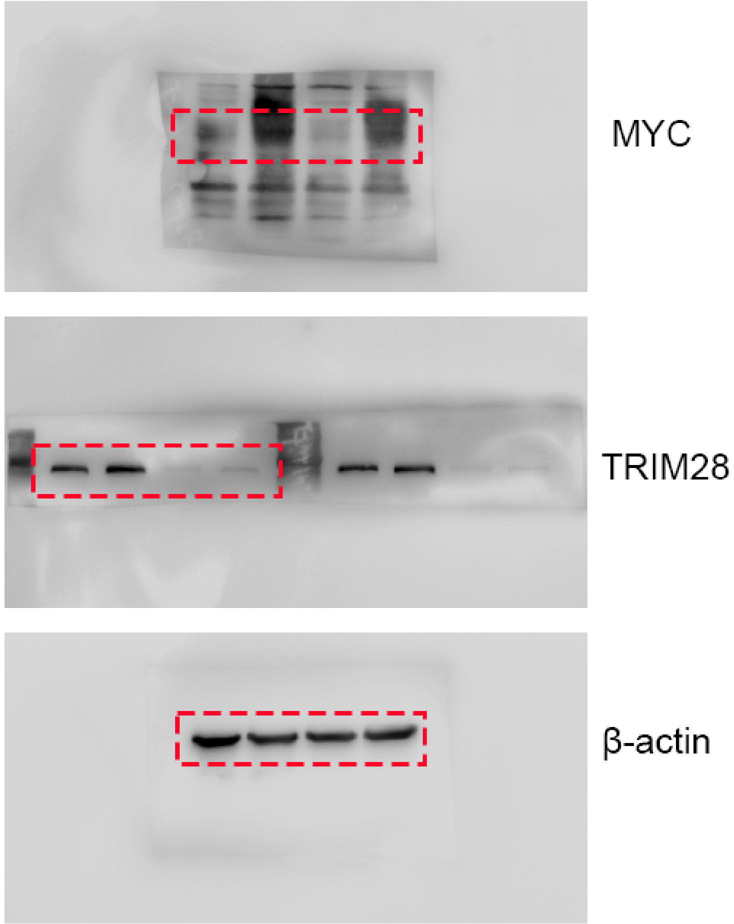

Supplementary Figure 7B

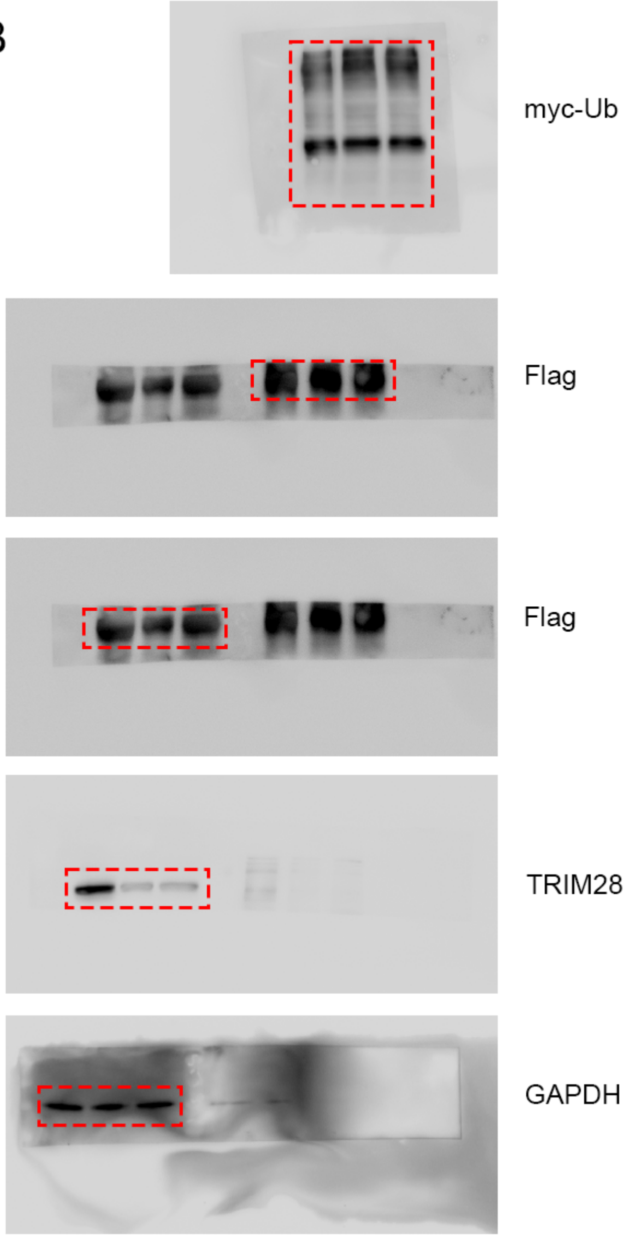

Supplementary Figure 7C

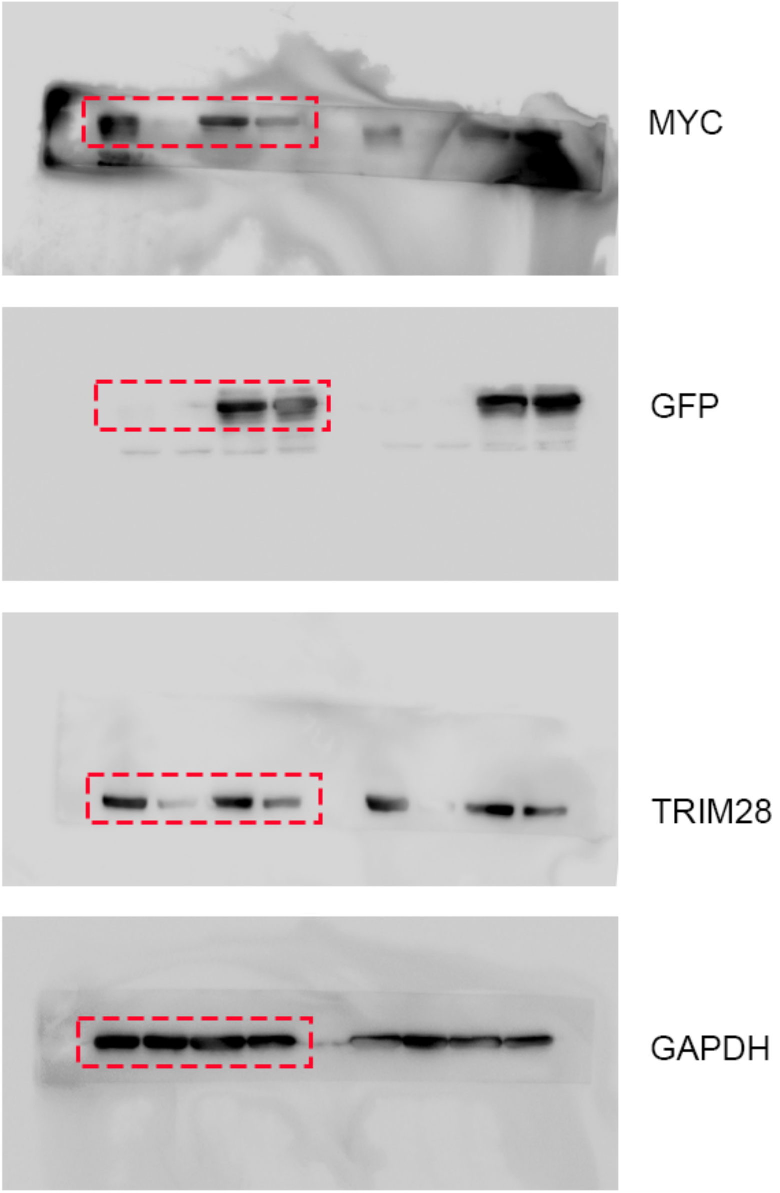

Supplementary Figure 7B

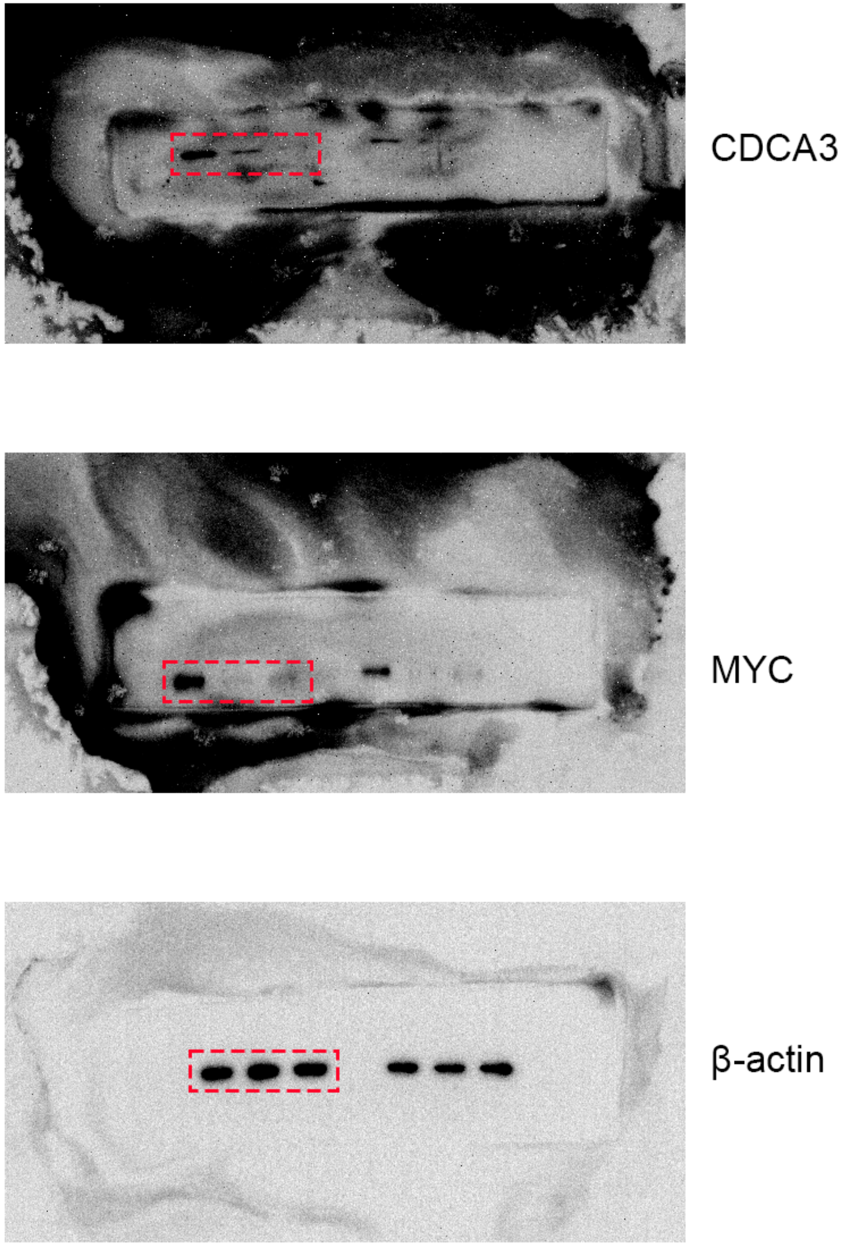

Supplement: Supplementary file 2 — Supplementary Material 2 [file 13046_2025_3325_MOESM2_ESM.pdf]
