# Supplementary material for: CDCA3-MYC positive feedback loop promotes bladder cancer progression via ENO1-mediated glycolysis
Source: J Exp Clin Cancer Res. 2025 Feb 20;44:63. doi: 10.1186/s13046-025-03325-7 (PMC11841255; doi:10.1186/s13046-025-03325-7)
Supplement: Supplementary file 3 — Supplementary Material 3 [file 13046_2025_3325_MOESM3_ESM.docx]

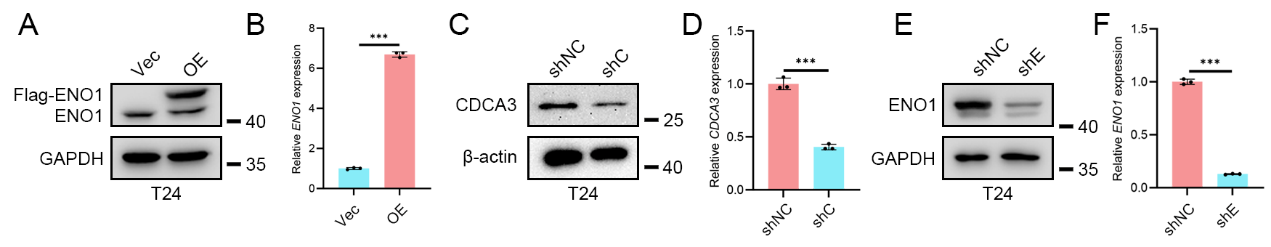


**Supplementary Figure 1. Analysis of transfection efficiency. (A)** WB analysis of overexpressing Flag-ENO1 plasmid in T24 BLCA cells. (B) qRT-PCR results of overexpressing Flag-ENO1 plasmid in T24 BLCA cells. (C) WB analysis of transfecting CDCA3 targeted lentivirus in T24 BLCA cells. (D) qRT-PCR results of transfecting CDCA3 targeted lentivirus in T24 BLCA cells. (E) WB analysis of transfecting ENO1 targeted lentivirus in T24 BLCA cells. (F) qRT-PCR results of transfecting ENO1 targeted lentivirus in T24 BLCA cells.


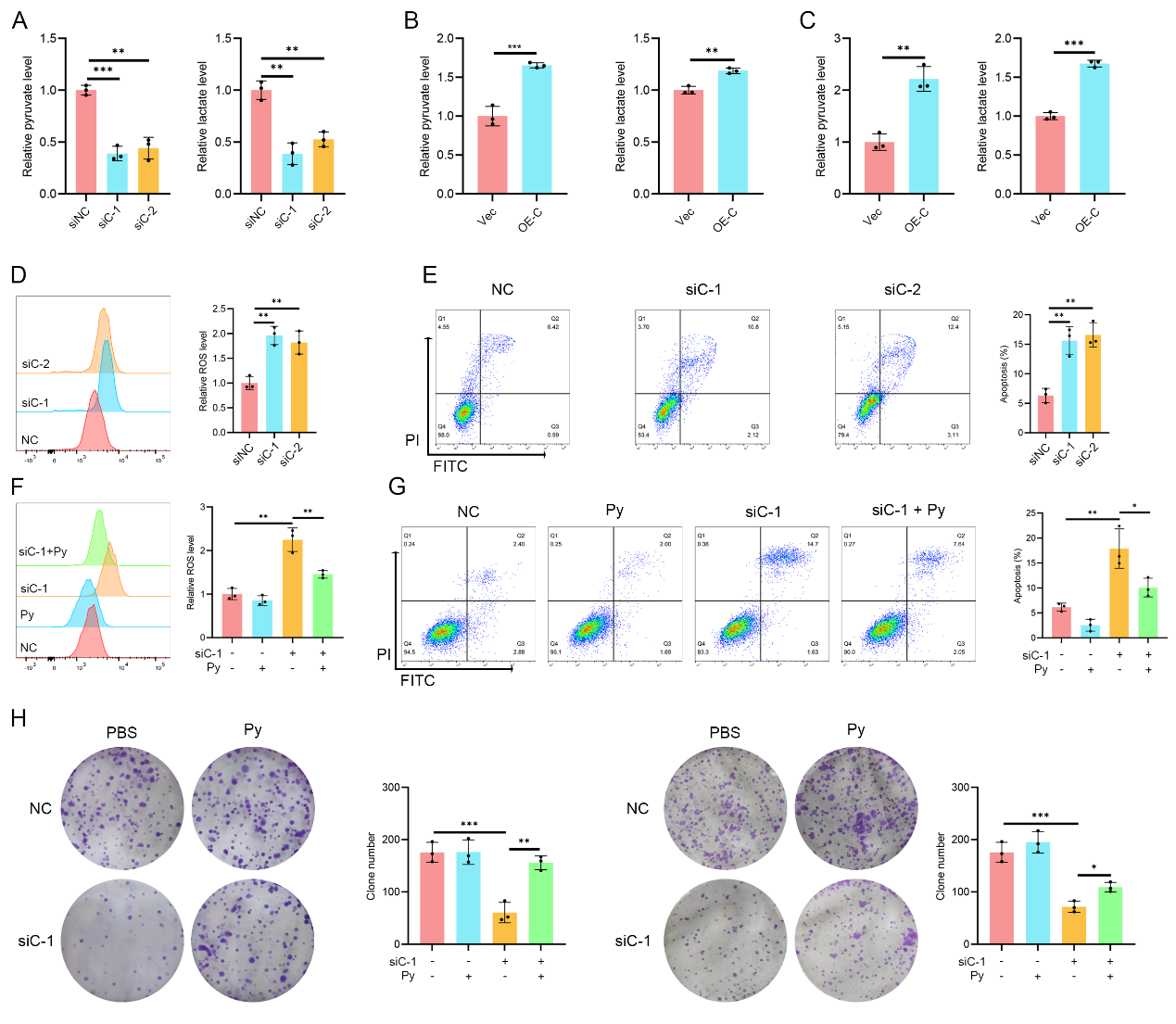


**Supplementary Figure 2. CDCA3 promotes BLCA progression via enhancing glycolysis.**

**(A)** Assessment of intracellular levels of pyruvate and lactate in 5637 BLCA cells following CDCA3 silencing (n = 3). **(B)** Assessment of intracellular levels of pyruvate and lactate in T24 BLCA cells following CDCA3 overexpression (n = 3). **(C)** Assessment of intracellular levels of pyruvate and lactate in 5637 BLCA cells following CDCA3 overexpression (n = 3). **(D)** Assessment of intracellular ROS levels following CDCA3 silencing in UM-UC3 BLCA cells (n = 3). **(E)** Assessment of apoptosis following CDCA3 silencing in UM-UC3 BLCA cells (n = 3). **(F)** Assessment of intracellular ROS levels following the addition of pyruvate (2 mM) to CDCA3-silenced UM-UC3 BLCA cells (n = 3). **(G)** Assessment of apoptosis following the addition of pyruvate (2 mM) to CDCA3-silenced UM-UC3 BLCA cells (n = 3). **(H)** Clone formation assays for T24 (left panel) and 5637 (right panel) BLCA cells with 2 mM pyruvate treatment and CDCA3-silencing (n = 3).


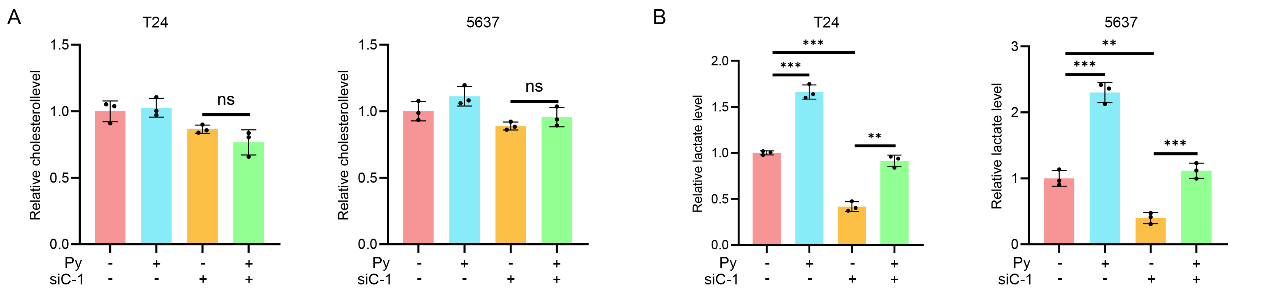


**Supplementary Figure 3. Detection of intracellular lactate and cholesterol. (A)** Detection of intracellular cholesterol in T24 and 5637 BLCA cells after silencing CDCA3 and supplementing pyruvate (2 mM, 24 hours) (n=3). **(B)** Detection of intracellular lactate in T24 and 5637 BLCA cells after silencing CDCA3 and supplementing pyruvate (2 mM, 24 hours) (n=3).


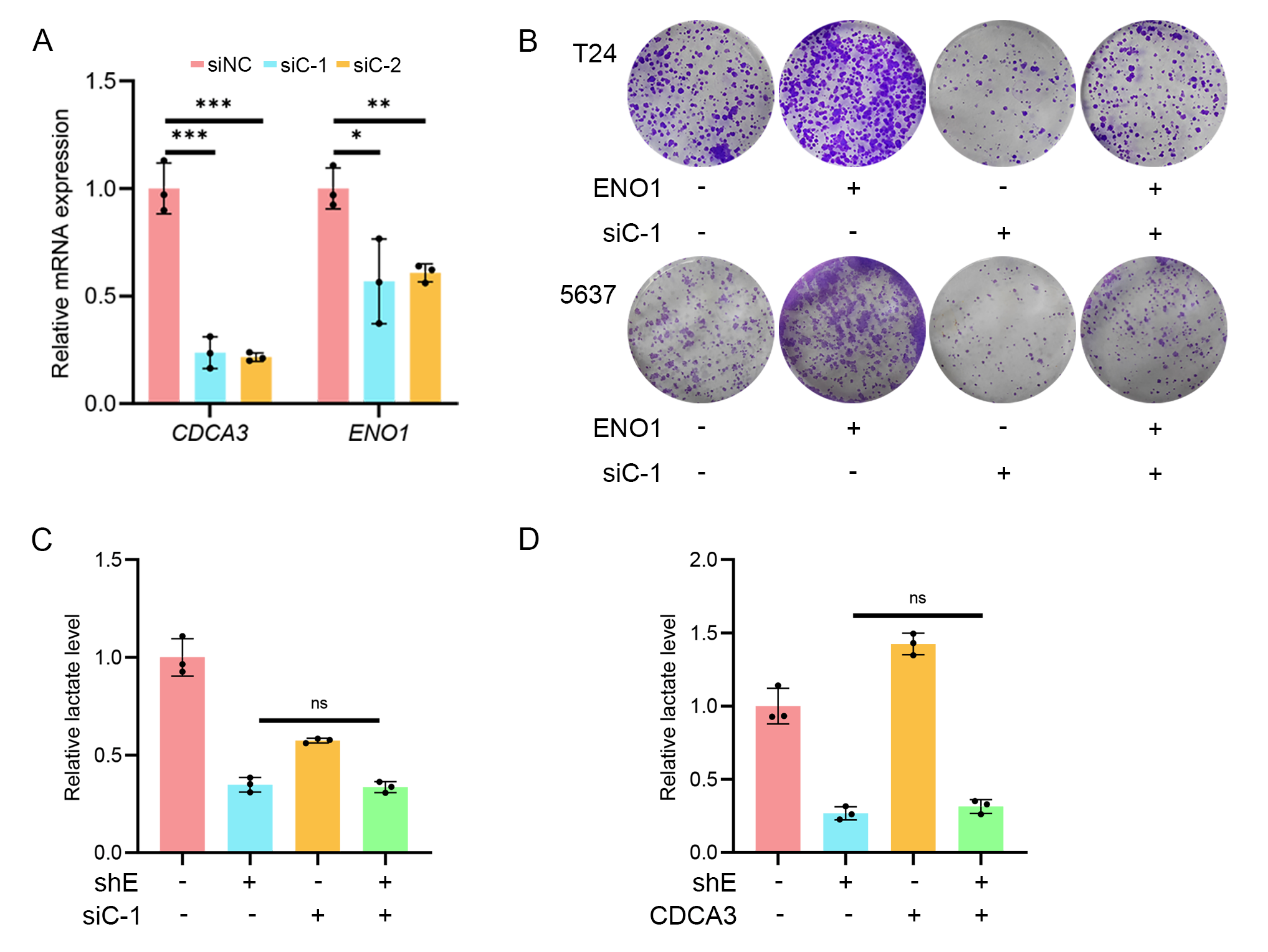


**Supplementary Figure 4. CDCA3 enhances BLCA glycolysis via α-Enolase. (A)** qRT-PCR analysis of ENO1 after silencing CDCA3 in 5637 BLCA cells. **(B)** Clone formation assays for T24 and 5637 BLCA cells with CDCA3-silencing and ENO1-overexpressing. **(C)** Detection of intracellular lactate in shENO1 T24 BLCA cells after silencing CDCA3. **(D)** Detection of intracellular lactate in shENO1 T24 BLCA cells after overexpressing CDCA3.


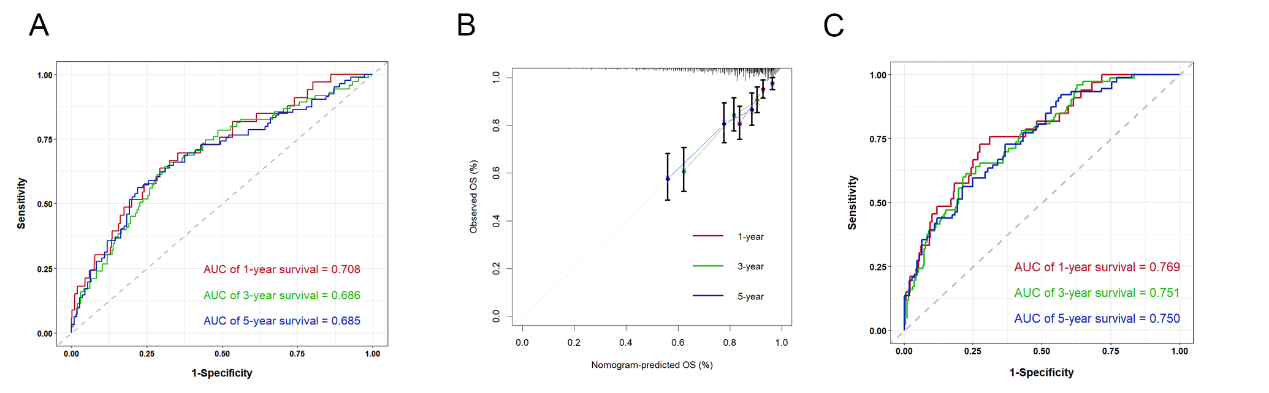


**Supplementary Figure 5.** **Construction of a risk predictive model.**

**(A)** ROC curve analysis of survival predictive model. **(B)** Calibration plots to verify the accuracy of the nomogram model. **(C)** ROC curve analysis of the nomogram model.


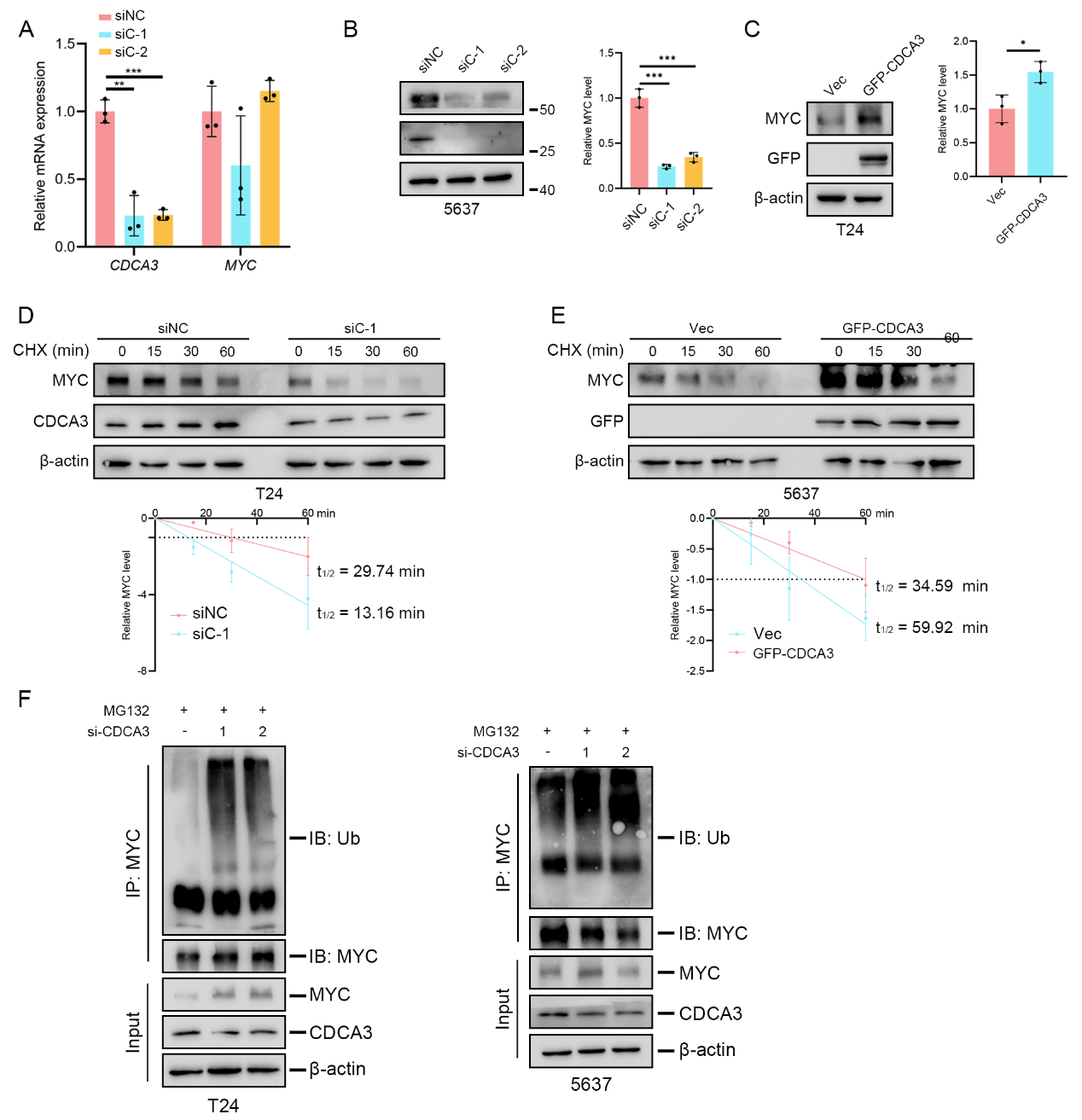


**Supplementary Figure 6.** **CDCA3 maintains MYC protein stability.**

**(A)** qRT-PCR results of *MYC* and *CDCA3* mRNA levels following CDCA3 silencing in 5637 BLCA cells. **(B)** Ubiquitination assay assessing the endogenous ubiquitination levels of MYC following CDCA3 silencing in T24 BLCA cells. **(C)** Ubiquitination assay assessing the endogenous ubiquitination levels of MYC following CDCA3 silencing in 5637 BLCA cells.


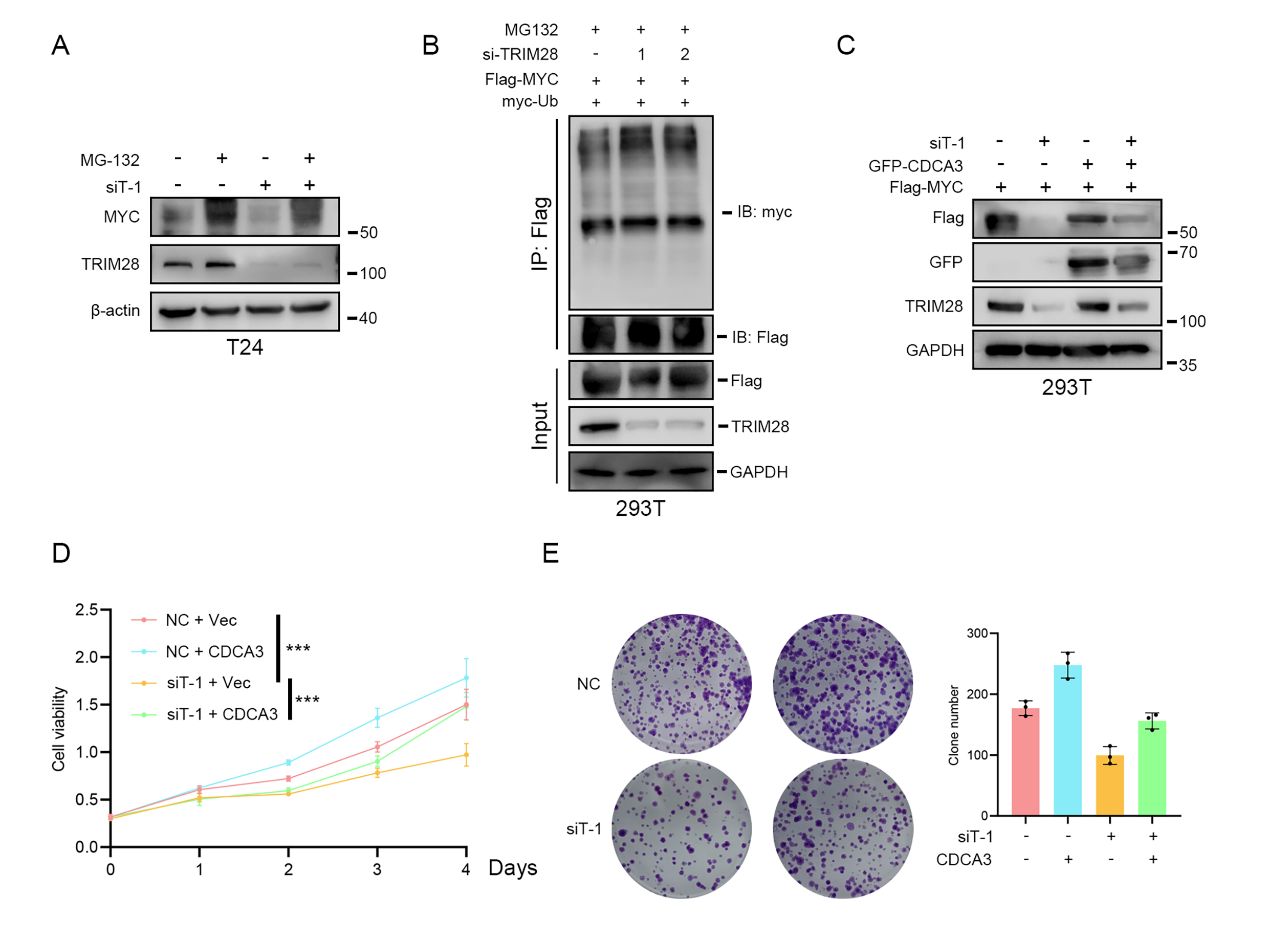


**Supplementary Figure 7.** **CDCA3 recruits TRIM28 to stabilize MYC.**

**(A)** WB results of MYC and TRIM28 protein levels following TRIM28 silencing and MG-132 treatment (10 μM, 4 hrs) in T24 BLCA cells. **(B)** Ubiquitination assay assessing the exogenous ubiquitination levels of MYC following TRIM28 silencing in T24 BLCA cells. **(C)** WB results of Flag-tag, GFP-tag and TRIM28 protein levels following TRIM28 silencing and GFP-CDCA3 overexpressing in 293T cells. (D) MTT assays for T24 BLCA cells with TRIM28 silencing and GFP-CDCA3 overexpressing. (E) Clone formation assays for T24 BLCA cells with TRIM28 silencing and GFP-CDCA3 overexpressing.


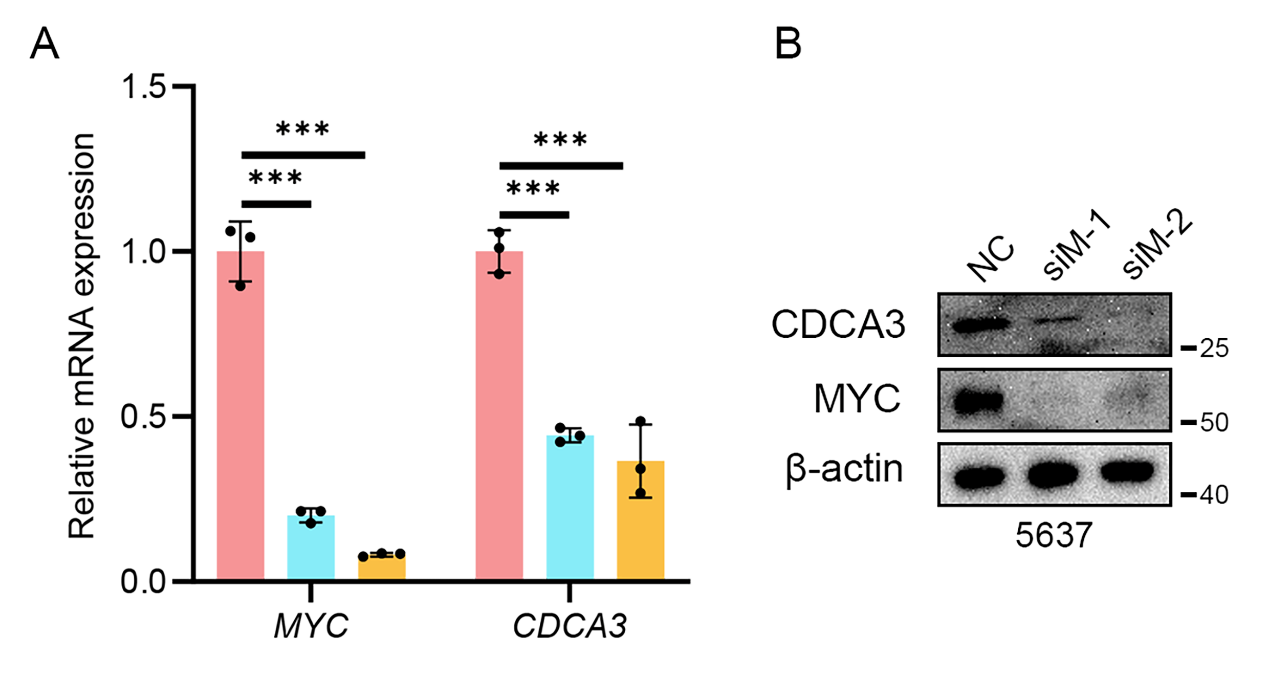


**Supplementary Figure 8. MYC promotes *CDCA3* transcription.**

**(A)** qRT-PCR of *CDCA3* and *MYC* mRNA level after silencing *MYC* in 5637 BLCA cells (n = 3). **(B)** WB results of CDCA3 and MYC protein level after silencing MYC in 5637 BLCA cells.

Supplementary Table 1. Sequence of specific siRNAs and lentivirus

| Target | Sense sequence (5’ - 3’) |
| --- | --- |
| siNC | UUCUCCGAACGUGUCACGUTT |
| siCDCA3-1 (siC-1) | GAGCAACUGGAGGGUCUUAAATT |
| siCDCA3-2 (siC-2) | CAUUCUUGGAACUGGACGACUTT |
| siMYC-1 (siM-1) | GCUUGUACCUGCAGGAUCUTT |
| siMYC-1 (siM-2) | GGAAGAAAUCGAUGUUGUUTT |
| siTRIM28-1 (siT-1) | GCGAUCUGGUUAUGUGCAATT |
| siTRIM28-2 (siT-2) | GCUCUGUUCUCUGUCCUGUCATT |
| shNC | TTCTCCGAACGTGTCACGT |
| shCDCA3 | GCTCTCCTACTCTTGGTAT |
| shENO1 | GCATTGGAGCAGAGGTTTA |

Supplementary Table 2. Primers used for qRT-PCR

| Target | Forward sense (5’ - 3’) | Reverse sense (5’ - 3’) |
| --- | --- | --- |
| *ACTB* | CATGTACGTTGCTATCCAGGC | CTCCTTAATGTCACGCACGAT |
| *CDCA3* | CTGGAGGGTCTTAAACATGCC | CACTGCTGGTCTTCATAGGTG |
| *MYC* | CTGGTGCTCCATGAGGAGA | CCTGCCTCTTTTCCACAGAA |
| *ENO1* | GTTCACAGCCAGTGCAGGAA | GGAGGCAGTTGCAGGACTTC |
| *TRIM28* | TTTCATGCGTGATAGTGGCAG | GCCTCTACACAGGTCTCACAC |

Supplementary Table 3. Antibodies used for WB, Co-IP and IHC

| Target | Species | Company | Dilution |
| --- | --- | --- | --- |
| β-actin | mouse | HuaBio, EM21002 | WB: 1/10000 |
| GAPDH | rabbit | Abways, AB0036 | WB: 1/10000 |
| CDCA3 | rabbit | ImmunoWay, YT0819 | WB: 1/1000 |
| MYC | rabbit | Proteintech, 10828-1-AP | WB: 1/1000  Co-IP: 1 μg/mL |
| TRIM28 | mouse | Proteintech, 66630-1-Ig | WB: 1/1000  Co-IP: 1 μg/mL |
| ENO1 | mouse | Santa Cruz, sc-390163 | WB: 1/5000 |
| HA-tag | mouse | Santa Cruz, sc-57592 | Co-IP: 1 μg/mL |
| HA-tag | rabbit | HuaBio, 0906-1 | WB: 1/1000  Co-IP: 1 μg/mL |
| GFP-tag | mouse | Santa Cruz, sc-9996 | WB: 1/1000 |
| GFP-tag | rabbit | Proteintech, 50430-2-AP | Co-IP: 1 μg/mL |
| Flag-tag | rabbit | HuaBio, 0912-1 | WB: 1/5000 |
| Flag-tag | mouse | Proteintech, 66008-4-Ig | Co-IP: 1 μg/mL |
| myc-tag | rabbit | Proteintech, 16286-1-AP | WB: 1/1000 |
| ubiquitin | rabbit | Proteintech, 10201-2-AP | WB: 1/1000 |
| Ki67 | rabbit | Proteintech, 27309-1-AP | IHC: 1/3000 |
| Secondary antibody | rabbit | ZSGB-Bio, ZB-2301 | WB: 1/10000 |
|  | mouse | ImmunoWay, RS23910 | WB: 1/10000 |

Supplementary Table 4. Chemicals used in experiments.

| Chemical | Source | Concentration |
| --- | --- | --- |
| Pyruvate | Topscience, T7717 | for *in vitro* assays: 2 mM  for *in vivo* assays: 1 g/kg |
| Gemcitabine | Topscience, T0251 | for *in vitro* assays: 500 nM or as noted  for *in vivo* assays: 10 mg/kg |
| MG132 | Topscience, T2154 | 10 μM |
| CHX (Cycloheximide) | MCE, HY-12320 | 50 μg/mL |

Supplementary Table 5. Clinical characteristics of patients in the nomogram model.

| Factor | Group | Alive | Dead | Total Number |
| --- | --- | --- | --- | --- |
| Grade | High | 98 | 55 | 153 |
|  | Low | 196 | 38 | 234 |
| Age | ≥65 | 182 | 67 | 249 |
|  | ＜65 | 112 | 26 | 138 |
| Risk | High | 132 | 61 | 193 |
|  | Low | 162 | 32 | 194 |

Supplementary Table 6. Metabolism related hallmarks via GSE32894 dataset.

| GeneSets | Size | NES | *P* value | FDR q value |
| --- | --- | --- | --- | --- |
| HALLMARK_GLYCOLYSIS | 181 | 2.25 | 0.000 | 0.000 |
| HALLMARK_CHOLESTEROL_HOMEOSTASIS | 70 | 2.02 | 0.000 | 0.003 |
| HALLMARK_HEME_METABOLISM | 159 | 1.04 | 0.374 | 0.680 |
| HALLMARK_FATTY_ACID_METABOLISM | 142 | 1.01 | 0.419 | 0.706 |
| HALLMARK_OXIDATIVE_PHOSPHORYLATION | 173 | 0.99 | 0.459 | 0.731 |
| HALLMARK_XENOBIOTIC_METABOLISM | 172 | 0.99 | 0.481 | 0.706 |

Supplementary Table 7. Metabolism related hallmarks via CCLE dataset.

| GeneSets | Size | NES | *P* value | FDR q value |
| --- | --- | --- | --- | --- |
| HALLMARK_GLYCOLYSIS | 196 | 1.33 | 0.029 | 0.166 |
| HALLMARK_OXIDATIVE_PHOSPHORYLATION | 184 | 1.29 | 0.224 | 0.207 |
| HALLMARK_FATTY_ACID_METABOLISM | 154 | 1.15 | 0.244 | 0.360 |
| HALLMARK_XENOBIOTIC_METABOLISM | 196 | 0.86 | 0.768 | 0.765 |
