# Supplementary material for: CDCA3-MYC positive feedback loop promotes bladder cancer progression via ENO1-mediated glycolysis
Source: J Exp Clin Cancer Res. 2025 Feb 20;44:63. doi: 10.1186/s13046-025-03325-7 (PMC11841255; doi:10.1186/s13046-025-03325-7)
Supplement: Supplementary file 4 — Supplementary Material 4 [file 13046_2025_3325_MOESM4_ESM.docx]

*Reviewer reports:*

*Reviewer #1: Please expand the introduction to provide a more detailed overview of BLCA, focusing on current challenges in its management, including diagnostic limitations and chemotherapy resistance.*

Response: We appreciate the great efforts by the reviewer to evaluate our manuscript. In our revision work, we added the introduction of NMIBC and MIBC and the current situation of BLCA treatment (from line 57 to line 72). We hope our revision work provides a clear description of BLCA.

*To contextualize the importance of the findings, a brief discussion on the clinical relevance of glycolysis and its role in BLCA progression should be included.*

Response: We appreciate this valuable suggestion. We have added a discussion about the implications of glycolysis on BLCA progression within the fourth paragraphs of the “Introduction” section (from line 86 to line 104). This addition highlights how glycolysis contributes to BLCA growth and therapeutic resistance, aligning it with the study’s focus.

*Please clearly define the working hypothesis and research objectives in the introduction to better guide the reader. It should also highlight the study's novelty, mainly how it builds upon or differs from previous research on CDCA3 and MYC in cancer, particularly for BLCA.*

Response: We fully agree with the reviewer’s kind suggestion. The role of CDCA3 in driving the progression of BLCA was previously reported by author Ph.D. Dexin Shen and his colleague Ph.D. Wei Liu. During the experimental design phase, we observed that multiple cell-cycle regulatory proteins influence cellular metabolism, prompting us to investigate whether CDCA3 also regulates the metabolic profile of BLCA (from line 118 to line 127). We have revised the “Introduction” section to include a clear working hypothesis and objectives. Additionally, we have elaborated on the novelty of the study, particularly how our findings build upon prior research on CDCA3 (from line 128 to line 131). This includes a description of CDCA3’s role as a potential regulator of glycolysis in BLCA, which adds a new perspective to its known functions.

*Specify the cell lines used and their origin are representative of high or low-grade BLCA.*

Response: We sincerely appreciate the kind reminder. In the revised manuscript, we have explicitly specified the cell lines used and categorized them based on their representation of high- or low-grade BLCA. These details can be found from line 140 to line 147 in the revised manuscript.

*Additional information related to the risk prediction model, including the type of the analysis methods and patient sample characteristics.*

Response: We appreciate the carefully reading of our manuscript by the reviewer. We added the analysis methods in the “Materials and methods” section from line 249 to line 274. Clinical characteristics statistics of patients included in our risk predictive model were listed in Table 2.

*Provide confidence intervals for the survival model and discuss its accuracy (e.g., AUC, sensitivity, specificity).*

Response: We sincerely thank the reviewer for identifying this oversight. Figures 3B and 3D were updated with confidence intervals. The accuracy of the survival model was updated in Supplementary Figure 5A. We sincerely hope the updated contents enhance the robustness of our analysis.

*Revise for grammatical consistency and clarity. Some sentences in the abstract and body are repetitive (e.g., duplication of the risk model statement).*

Response: We sincerely appreciate your valuable feedback. The manuscript has undergone professional language editing to ensure grammatical accuracy and clarity. Redundant sentences, especially in the abstract and sections discussing the risk model, have been revised or removed. A language editing certificate has been included in the supplementary materials.

Conclusion: We sincerely appreciate the reviewer’s thorough evaluation of our manuscript and valuable suggestions. Your kind suggestions have helped us make up for the shortcomings in our initial manuscript and our group benefit a lot during the revision process. We hope our point-to-point responses address your concerns and improve the overall quality of our work.

*Reviewer #2: In this manuscript Shen D. and colleagues explore the role of the cell cycle-related protein CDCA3 as a metabolic regulatory player in bladder cancer. By means of knockdown experiments in bladder cancer cells in vitro and in mouse xenografts in vivo, the authors uncover a regulatory circuit between CDCA3 and MYC oncogene in the control of glycolytic-dependent bladder cancer growth and sensitivity to the anti-cancer drug gemcitabine. Of interest is also the proposed risk model where CDCA3 and the glycolytic enzyme ENO1 are used as prognostic markers of patients' survival. Despite the interesting results showed in the manuscript, It requires major revision.*

Response: We sincerely appreciate the reviewer’s thorough evaluation of our manuscript and the positive feedback on our findings. Your constructive suggestions have provided valuable insights that will help us improve the quality and clarity of our work. We are grateful for your recognition of the significance of our study and your thoughtful recommendations that highlight areas for improvement.

In the following point-by-point responses, we have addressed each of your concerns in detail and implemented the necessary revisions to enhance the manuscript. We hope our responses adequately address your suggestions and improve the overall quality of our work.

*Major comments:*

1) *Introduction:*

*This section must be totally revised according to the following points: 
- there is a prevalent lack of references (just an example is lane 85: "Researches have previously established that CDCA3 enhances the progression of a range of human malignancies", which refs?)*

Response: We sincerely appreciate the reviewer’s careful reading of our manuscript and for highlighting the need for additional references. As mentioned in lines 108 to 116 of the manuscript, we have introduced the role of CDCA3 in promoting the progression of various malignancies, including prostate cancer, lymphoma, and clear cell renal cell carcinoma (ccRCC). Additionally, CDCA3 has been previously reported to contribute to the progression of bladder cancer by our author Dexin Shen [1, 2].

In response to the reviewer’s valuable suggestion, we have now incorporated appropriate references to support the statement in line 109 and other relevant sections of the manuscript. These references [3-5] highlight the broader oncogenic role of CDCA3 across various cancer types, ensuring a more comprehensive and well-supported discussion.

We greatly appreciate your insightful comments, which have helped us to improve the accuracy and completeness of our manuscript.

*- there is an overall use of vague terms and sentences (e.g. lane 95 "Notably, CDCA3 might influence glycolysis in tumor cells" or lane 100 "CDCA3 engages directly with MYC")*

Response: We sincerely thank the reviewer for the valuable suggestion. To address concerns regarding vague terms and sentence clarity, we have revised the manuscript to enhance precision and ensure grammatical consistency. Ambiguous statements have been refined to provide clearer and more direct explanations, and redundant sentences have been revised or removed where appropriate.

Furthermore, the manuscript has undergone professional language editing to improve readability and clarity. A certificate confirming the completion of language editing has been included in the supplementary materials for reference.

We appreciate the reviewer’s constructive feedback, which has helped us improve the overall quality and clarity of our manuscript.

*- English needs to be revised and a more rigorous scientific phrasing must be used.*

Response: We sincerely thank the reviewer for highlighting this important issue. In response to your valuable suggestion, we have subjected the manuscript to professional language editing to enhance grammatical accuracy, readability, and scientific rigor. The revised manuscript now adheres to a more precise and rigorous scientific writing style.

A certificate confirming the completion of professional language editing has been included in the supplementary materials for your reference.

We greatly appreciate your feedback, which has been instrumental in improving the overall quality of our manuscript.

*2) Material and methods: this section should be improved with more details. A deeper explanation should be made for all paragraphs.*

Response: We sincerely appreciate the reviewer’s thorough evaluation of our manuscript and valuable feedback. In response to your suggestion, we have provided additional details and clarifications in the “Materials and Methods” section to ensure a more comprehensive and precise description of our experimental procedures. The revised section now includes expanded explanations for all methodologies to enhance reproducibility and clarity.

We hope that these revisions meet the reviewer’s expectations and further improve the quality of our manuscript. Thank you again for your insightful comments.

*3) Results:
* Figure 1:
a.  a major issue is the use of one BCLA cell line T24 for demonstrating the initial part relative to figure 1. These key experiments need to be performed at least in another cell line to demonstrate a cell- type independent phenotype.*

Response: We sincerely appreciate the reviewer’s careful investigation of our manuscript and for highlighting this important concern. We would like to clarify that the related experiments have been conducted in an additional BLCA cell line and are presented in Supplementary Figure 2A-C.

We acknowledge that the corresponding figure legends were not clearly referenced in the manuscript, and we apologize for any confusion this may have caused. In the revised manuscript, we have updated the figure legends and references to ensure clarity and accurate representation of the data.

We hope this clarification addresses the reviewer’s concern, and we greatly appreciate your constructive feedback.

*b. the authors interpret the reduced content of pyruvate and lactate as a readout of reduced glycolytic capacity dependent on CDCA3 loss in BCLA cells. This cannot be demonstrated without performing a direct measurement of glycolytic capacity coupled to mitochondrial metabolic profiling (gold standard is ECAR and OCR measurement through seahorse technology, but other specific assays also exist).*

Response: We thank the reviewer for pointing this out. Pyruvate and lactate are pivotal products of glycolysis and the alteration of pyruvate and lactate is able to reflect the fluctuation of glycolytic capacity to some extent. Certainly, as you kindly suggest, a direct measurement of ECAR and OCR more clearly show the glycolytic capacity. We performed the measurement of ECAR and OCR, and found silencing CDCA3 inhibited glycolytic capacity while elevated O_2_ consumption (Figure 1C). Combined the measurement of intracellular pyruvate and lactate, and the alteration of ROS, we believe our work is able to clearly clarify the regulatory role of CDCA3 on BLCA glycolysis.

*c. it is confusing to me to demonstrate the dependency on glycolytic pathway in CDCA3 silenced cells by adding pyruvate to the medium or by its in vivo administration. As the authors say pyruvate is the final glycolytic metabolite, but it* *is also required for other metabolic routes. The pyruvate-dependent rescue could be ideally due to an addiction to this specific metabolite rather than on glycolysis. Otherwise it could be the mere effect of boosting glucose utilization. Can the authors address this specific issue?*

Response: Thank you for raising this important concern. To address this, we provide the following five points clarifying our experimental design and the revisions made in response to your suggestions.

Firstly, we investigated many other articles which once used pyruvate and found the mostly mentioned concentration is 1 mM [6-11] which aligns with our experiments. The highest concentration reported was 20 mM [12]. Our preliminary experiments indicated that exogenous pyruvate supplementation at concentrations between 1-5 mM had no significant effect on BLCA cell proliferation, whereas concentrations above 5 mM inhibited cell growth. Therefore, 2 mM pyruvate is an ideal concentration for our rescue assays.

Secondly, pyruvate has been reported be used in experimental animals and “**supplementation of pyruvate, the end product of glycolysis, significantly restored the delayed onset and slower growth of mammary tumors**” [13] (Park et.al, ***Cell Metab***, 2021 Dec 7;33(12):2380–2397.e9.). Our experiments also demonstrate that pyruvate supplementation is able to rescue the proliferation of xenografted tumors (Figure 1I-M). Therefore, we believe it is reasonable to use pyruvate to perform the rescue assays.

Thirdly, given its high predictive accuracy, GSEA is widely employed to infer gene functions in specific diseases; hence, we used it to predict the role of CDCA3 in BLCA progression. As you correctly noted, pyruvate “*is also required for other metabolic routes*”, Therefore, we re-evaluated our GSEA results to confirm whether CDCA3 is linked to other metabolic processes. We found that “**HALLMARK_GLYCOLYSIS**” is the only metabolism-related gene set positively correlated with CDCA3 in both the GSE32894 and CCLE databases, underscoring the significance of glycolysis in CDCA3-mediated regulation of BLCA. In the revised manuscript, we have included two supplementary tables (Supplementary Table 6 and Supplementary Table 7) detailing the metabolism-related hallmarks.

Fourthly, we reviewed other high-quality articles to identify the relationship between different metabolic pathways and BLCA progression. We discovered several interesting yet contradictory findings. For example, CPT1B, the rate-limiting enzyme of fatty acid β-oxidation, is downregulated in high-grade BLCA compared to low-grade BLCA samples, and the fatty acid β-oxidation product is significantly reduced in high-grade BLCA. Overexpression of CPT1B hampers BLCA growth despite increased β-oxidation activity [14]. However, it has also been reported that treatment with etomoxir, an inhibitor of fatty acid oxidation and CPT-1, suppresses BLCA progression by decreasing ATP and NADPH levels through the inhibition of fatty acid oxidation [15]. Additionally, although DHCR7, a key enzyme in cholesterol synthesis, is reported to be closely associated with BLCA metastasis, silencing or overexpressing DHCR7 had no effect on BLCA growth. Cholesterol supplementation was only shown to promote BLCA metastasis [16]. Further investigation is needed to elucidate the role of cholesterol in promoting BLCA progression.

Fifthly, we fully acknowledge your valid concern regarding the potential activation of other metabolic pathways by pyruvate supplementation. We measured intracellular cholesterol levels, as it may also be regulated by CDCA3, according to Supplementary Table 6. However, silencing CDCA3 resulted in only a minimal decrease in cholesterol, and pyruvate supplementation did not restore the cholesterol level (Supplementary Figure 3A), whereas pyruvate supplementation effectively rescued the decreased lactate level (Supplementary Figure 3B). We attribute this phenomenon to the tendency of BLCA cells to utilize intracellular pyruvate for glycolysis rather than other metabolic pathways to sustain energy production.

To conclude, your constructive comments is pivotal for us to make up the omissions of our work and we hope our explanations are effective in solving your concerns.

** Figure 2:* 
*a. in figure 2B the data about control cells are missing and there is no result in this way*

Response: We sincerely appreciate your reminder. The corresponding data for the control cells have been updated and are now presented in Figure 2B and Supplementary Figure 4A.

*b. Quantification by mean densitometric analysis is missing for WB blot in figure 2C.*

Response: We sincerely appreciate your valuable feedback. The densitometric analysis of the Western blot results in Figure 2C has been conducted, and the corresponding data are now included in Figure 2C.

*c. The authors comment on figure 2D-E saying that ENO1 over-expression boosts lactate levels and clonogenic capacity in the two BLCA cell lines. This is true also for control cells, where CDCA3 is present, so this dependency is not convincing as wild-type cells display the same behavior.*

Response: We sincerely appreciate the reviewer’s insightful comments and respectfully provide clarification. Our study demonstrates that CDCA3 regulates BLCA glycolysis through ENO1, making it appropriate to perform rescue assays by overexpressing ENO1 after silencing CDCA3. The role of ENO1 in promoting tumor progression via enhancing glycolysis has been well-established in various cancers [17-20], and Author Dexin Shen have also reported the pivotal role of ENO1 in promoting BLCA progression [21]. Therefore, it is not surprising that ENO1 overexpression in wild-type cells, where CDCA3 is present, similarly boosts lactate levels and clonogenic capacity. We respectfully hope that this explanation addresses the reviewer’s concerns.

*d. a WB or real-time qPCR showing the efficiency of ENO1 over-expression is totally missing.*

Response: We appreciate the reviewer’s kind suggestion. WB and qRT-PCR assays were conducted to confirm the efficiency of ENO1 overexpression, and the results are now presented in Supplementary Figure 1B.

*About figure 2-related results the authors comment 'Collectively, these results demonstrate that ENO1 mediates the regulation of CDCA3 on BLCA glycolysis'. This is not supported by the data. The authors should perform double knockdown experiments to directly demonstrate this dependency.*

Response: We sincerely appreciate the reviewer’s insightful comments and completely agree with the concern raised. To directly address this issue, we conducted additional experiments by generating an ENO1-silenced T24 cell line using a specific lentivirus (sh-ENO1), with appropriate negative controls (sh-NC). The efficiency of ENO1 silencing was validated through Western blot (WB) and quantitative real-time PCR (qRT-PCR) assays, and the corresponding results are presented in Supplementary Figure 1E-F.

Our results demonstrate that silencing ENO1 significantly reduced intracellular lactate production in T24 cells. Notably, further silencing of CDCA3 in ENO1-silenced cells did not result in any additional reduction in lactate levels (Supplementary Figure 4C). Furthermore, overexpression of CDCA3 failed to restore lactate levels in ENO1-silenced cells (Supplementary Figure 4D). These findings provide compelling evidence that CDCA3 regulates glycolysis in BLCA cells through ENO1.

Mechanistically, ENO1 is a key enzyme in glycolysis, catalyzing the conversion of 2-phosphoglycerate to phosphoenolpyruvate (PEP). Our findings indicate that without ENO1, CDCA3’s regulatory effect on glycolysis is abolished. These results provide direct evidence of the dependency of CDCA3 on ENO1 in regulating glycolysis, furthering our understanding of metabolic regulation in BLCA.

We are grateful for the reviewer’s valuable suggestions, which have significantly contributed to improving the clarity and robustness of our study. We hope that our revisions and additional experimental data sufficiently address the reviewer’s concerns and further strengthen our manuscript.

** Figure 4:*

a. *The methodology used to establish the gemcitabine-resistant T24 cell line should be clearly explained in the manuscript. In particular, this information is completely missed in the Material and Methods section, and in the Results or figure legend sections. Indeed, some relevant informations are missing in the text (e.g. gemcitabile IC50 of* *resistant and parental cell line, the time required to generate the resistance, if the IC50 of resistant cell line is in line with clinically relevant concentrations).*

Response: We sincerely thank the reviewer for their thorough evaluation of our manuscript and for pointing out this important omission. In response, we have supplemented the “Materials and Methods” section (lines 265 to 272) with a detailed description of the methodology used to establish the gemcitabine-resistant T24 cell line. Additionally, the IC50 values for both the resistant and parental cell lines have been incorporated into Figure 4B, providing a clearer representation of the differences in drug sensitivity.

Regarding the clinical relevance of our experimental conditions, we would like to clarify that in bladder instillation chemotherapy, 1 g of gemcitabine hydrochloride is dissolved in 50 mL of saline, resulting in a theoretical concentration of approximately 66.74 mM. This concentration is considerably higher than those used in our in vitro experiments. However, given that intravesical gemcitabine therapy involves a retention time of only 1 to 2 hours, the exposure conditions differ significantly from continuous in vitro treatment, which accounts for the observed discrepancies.

Our research aims to identify novel molecular targets to enhance gemcitabine sensitivity in bladder cancer, and we are committed to further exploring this direction in future studies. We appreciate the reviewer’s valuable feedback and look forward to incorporating their guidance to improve the quality and impact of our work.

*b. The authors use ENO1 overexpression to demonstrate the dependency of gemcitabine sensitivity of CDCA3 silenced cells. In the viability analyses it seems that again ENO1 overexpression rescues cell viability under gemcitabine treatment also in control cells at the same rate as silenced cells. What is about statistics in control cells with and w/o ENO1 over-expression?*

Response: We appreciate the reviewer’s thoughtful comments. Upon revisiting the data presentation, we realized that we mistakenly presented the results in a simplified single-column format, which failed to properly show our results. This oversight led to the reviewer’s misunderstanding, and we apologize for the confusion.

In response to this concern, after re-grouping the data according to whether CDCA3 was silenced, we found that over-expressing of ENO1 almost restored the sensitivity of BLCA to GEM to an siNC group, indicating that ENO1 significantly reversed the drug sensitivity changes caused by knocking down CDCA3 (Rebuttal Figure 1). We also chose to present the results using drug sensitivity curves, which provide a clearer demonstration of the effects. Our updated analysis shows that ENO1 over-expression significantly reversed the increased sensitivity to GEM observed in CDCA3-silenced BLCA cells (shC + Vec) to the controlled cells (shNC + Vec). Therefore, we believe our work clearly shows the role of CDCA3-ENO1 axis in regulating BLCA sensitivity to GEM.

**Rebuttal Figure 1.** Effect of silencing CDCA3 and overexpressing ENO1 on the cell viability of T24 cells under 200 nM GEM treatment (48 hrs, n = 5).

We sincerely hope these revised experiments and statistical analyses address the reviewer’s concerns and provide clearer evidence supporting our conclusions.

*c. Concerning suppl. figure 1 showing the efficacy of lentiviral transduction. Can you provide densitometric analysis? What is the downregulation rate?*

Response: We appreciate the reviewer’s thoughtful comments. We supplemented corresponding qRT-PCR results to further show the efficiency of the lentivirial transduction. We appreciate the reviewer’s valuable suggestions, which has helped improve the rigor of our data presentation.

*d. The in vivo experiment with Gemcitabine treatment shows a strong cancer growth and mice weight reduction induced by treatment alone and by CDCA3 downregulation alone. However, the effect of gemcitabine on CDCA3 silenced tumors paradoxically seems to be lower than that observed in non-silenced ones. How can you explain this apparent lack of synergic or additive effect?*

Response: We sincerely appreciate the reviewer’s thoughtful comments and careful evaluation of our study. We would like to provide a detailed clarification regarding the observed effects of gemcitabine treatment on CDCA3-silenced tumors.

Firstly, we acknowledge the reviewer’s concern regarding the additive effect of gemcitabine on CDCA3-silenced tumors. However, based on the data presented in Figure 4J, we believe that CDCA3-silenced BLCA cells are actually more sensitive to gemcitabine treatment compared to wild-type BLCA cells. To support this, we provide detailed tumor weight data in Rebuttal Table 1.

As we can find in the table, the largest sample in group “shNC + PBS” (1119.4 mg) is 3.13-fold heavier than the largest sample in group “shNC + GEM” (357.9 mg) and the largest sample in group “shC + PBS” (580.8 mg) is 3.22-fold heavier than the largest sample in group “shC + GEM” (180.3 mg). The largest sample in group “shNC + PBS” (1119.4 mg) is 4.83-fold heavier than the smallest sample in group “shNC + GEM” (231.9 mg), and the largest sample in group “shC + PBS” (580.8 mg) is 9.03-fold heavier than the smallest sample in group “shC + GEM” (64.3 mg). The smallest sample in group “shNC + PBS” (815.0 mg) is 3.51-fold heavier than the smallest sample in group “shNC + GEM” (231.9 mg), and the smallest sample in group “shC + PBS” (241.2 mg) is 3.75-fold heavier than the smallest sample in group “shC + GEM” (64.3 mg). We believe the comparison of these data can demonstrate that silencing CDCA3 increases the effect of gemcitabine

Secondly, BLCA is known to be highly heterogeneous, which can lead to variable responses to chemotherapy. This variability is reflected in our xenograft model, where differences in tumor sizes among individual animals likely contributed to the observed variation in treatment response. Despite this inherent variability, the overall trend suggests that CDCA3 silencing enhances gemcitabine sensitivity.

Lastly, we fully acknowledge that tumor heterogeneity is a critical factor influencing chemotherapy outcomes, both in preclinical models and clinical practice. As such, we have listed tumor heterogeneity as an important future direction for our research group.

We hope our explanation, along with the additional data, sufficiently addresses the reviewer’s concerns and clarifies the observed experimental results.

| shNC + PBS | shNC + GEM | shC + PBS | shC + GEM |
| --- | --- | --- | --- |
| 1119.4 mg | 357.9 mg | 580.8 mg | 180.3 mg |
| 1031.4 mg | 289.6 mg | 423.6 mg | 140.8 mg |
| 861.8 mg | 286.6 mg | 324.3 mg | 107.9 mg |
| 835.7 mg | 251.1 mg | 236.6 mg | 91.5 mg |
| 815.0 mg | 231.9 mg | 241.2 mg | 64.3 mg |

**Rebuttal Table 1**. Weight of xenograft samples in Figure 4J

** Figure 5:
a. WB in figure 5B and C showing MYC protein modulation after CDCA3 silencing/overexpression is convincing but again the densitometric analysis is missing.*

Response: We thank the reviewer for the kind reminding. Corresponding densitometric analysis of the WB assays were updated in Figure 5B&C and Supplementary Figure 6B&C.

*b. Analysis of MYC stability in figure 5 D-F lacks densitometric analysis. CDCA3 silencing efficiency in fig. 5B seems to be very low.*

Response: We thank the reviewer for the kind suggestions. Analysis of MYC stability were updated in Figure 5D&E and Supplementary Figure 6D&E. Your kind suggestions help us better demonstrate the regulation of CDCA3 on MYC stability. As for Figure 5B, we updated the results to better present our experiments. We hope our revised experiments satisfactorily address your concerns.

*c. Legend of MYC and CDCA3 mRNA expression is missing in Supplementary Figure 5A. Please, add legend in histogram.*

Response: We sincerely thank the reviewer for the careful evaluation of our manuscript and for pointing out this oversight. We apologize for the omission and have now added the appropriate legend in Supplementary Figure 5A to clearly indicate the different treatment conditions of 5637 BLCA cells.

We appreciate your valuable feedback, which has helped us improve the clarity and completeness of our data presentation.

** Figure 6:
Regarding the IP assay of figure 6B, densitometric analysis must be shown for both IP of MYC and CDCA3. Additionally, it is not clear which sample is input. Input should be loaded of both IgG and IP: MYC/CDCA3 samples, and* *the housekeeping protein of input must be shown. Regarding the immunoprecipitation of MYC, it is ambiguous that MYC protein is present in IgG and in IP: MYC, as well, it is ambiguous that CDCA3 is absent in the input sample and not in IgG sample for IP: CDCA3. Since the molecular weight of MYC and CDCA3 overlaps with IgG, it seems that the band of MYC and CDCA3 in IP is related to IgG instead of MYC and CDCA3.*

Response: We appreciate the reviewer’s thoughtful comments and careful review of our manuscript. Below, we provide a detailed response to the concerns raised:

Firstly, we acknowledge the reviewer’s concern about potential interference from the IgG heavy chain in our initial Co-IP and WB experiments. In our revised experiments, we used secondary antibodies capable of eliminating IgG heavy chain signals, which effectively removed this interference. The revised results in Figure 6B clearly demonstrate the endogenous interaction between CDCA3 and MYC without ambiguity.

Secondly, we have repeated the endogenous Co-IP experiments and included GAPDH as a loading control for the input samples, as requested. Input samples were loaded for both IgG and IP conditions to ensure equal input, and GAPDH was used to verify loading consistency (Figure 6B). We believe this revision adequately addresses the reviewer’s concern.

Thirdly, we carefully reviewed high-quality articles presenting Co-IP results in a manner similar to ours and noted that densitometric analysis is not commonly performed in such cases (Rebuttal Figure 2**-**5) [22-25]. Additionally, because we used secondary antibodies that remove IgG heavy chains in our revised experiments, the resulting bands are clear and unambiguous, making densitometric analysis unnecessary. Moreover, we repeated the Co-IP and WB experiments three times, and we present the other two replicates to demonstrate our revision work (Rebuttal Figure 6). The results were consistent across replicates, further supporting the reliability of our findings. Thus, we believe densitometric analysis is not necessary to demonstrate the interaction between CDCA3 and MYC.

Rebuttal Figure 2 “(E) Co-IP of AR with USP11 (Left panel) or Co-IP of USP11 with AR (Right panel) in Rv1 cells.” ***Proc Natl Acad Sci U S A***. 2024 Jul 30;121(31): e2403331121.

Rebuttal Figure 3 “a, b Reciprocal co-IP analysis of PHGDH and PRMT1 in HEK293T, Huh7 and PLC/PRF/5 cells. IgG was used as a negative control.” ***Nat Commun***. 2023 Feb 23;14(1):1011.

Rebuttal Figure 4 “C) T24 cells were subjected to co-IP by using anti-YEATS4 or anti-IgG to detect endogenous HUWE1.” ***Adv Sci (Weinh)***. 2024 Jun;11(22): e2310146.

Rebuttal Figure 5 “c, d Reciprocal co-IP analysis of PRMT1 and FBXO7 in Huh7 and PLC/PRF/5 cells. IgG was used as a negative control.” ***Nat Commun***. 2024 Jun 5;15(1):4790.

Rebuttal Figure 6. Co-IP assays performed in UM-UC3 and T24 cells. (A) Two replicates performed in UM-UC3 cells. (B) Two replicates performed in T24 cells.

With sincere respect and regards, we hope our revised experiments and explanations satisfactorily address the reviewer’s concerns.

** Figure 7F:* *densitometric analysis of endogenous IP should be addressed.*

Response: We sincerely thank the reviewer’s kind reminder. Similar to the previous question, we used secondary antibodies that remove IgG heavy chains in our revised experiments, the resulting bands are clear and unambiguous, making densitometric analysis unnecessary. Our results clearly demonstrated the interactions between TRIM28, MYC, and CDCA3, and the presence of housekeeping protein GAPDH was used to verify loading consistency (Figure 7F). Similarly, we present the other two replicates to demonstrate our revision work. The results were consistent across replicates, further supporting the reliability of our findings (Rebuttal Figure 7). With sincere regards, we believe our revision work is able to solve your kind concern.

Rebuttal Figure 7. Two replicates performed in T24 cells.

*Minor comments:
- the authors use siC1 or siC2 in the panels to indicate CDCA3 siRNA condition. They use the term NC as a control. It is not stated in the figure legends what 'NC' means. Did the authors use control siRNAs? It is mandatory to specify this point.*

Response: We appreciate the carefully reading of our manuscript by the reviewer. Control siRNA was used and was indicated as “NC” in the initial version of our manuscript. We checked our manuscript and figures and each “NC” was corrected by “siNC”.

*- Figure 3-4. It could be interesting to understand the correlation of CDCA3 and ENO1 expression with the efficacy of gemcitabine-based treatments. Do the authors have any data on the correlation of CDCA3 and ENO1 expression with chemotherapy efficacy in BLCA patients?*

Response: We thank the reviewer for highlighting this interesting and important question. As mentioned in the “Introduction” section (lines –), the progression from non-muscle-invasive bladder cancer (NMIBC) to muscle-invasive bladder cancer (MIBC) is a key factor influencing chemotherapy response and patient prognosis. Previous research by our group and others has shown that the expression of CDCA3 and ENO1 is closely associated with tumor progression and muscle invasion [1, 21], which suggests a potential role for these genes in determining chemotherapy efficacy.

Although we currently lack clinical samples to directly analyze the correlation of CDCA3 and ENO1 expression with gemcitabine efficacy in BLCA patients, our experimental data support this hypothesis.

For instance: Silencing CDCA3 or ENO1 in BLCA cells reduces glycolysis, which may impair the energy supply required for tumor survival under chemotherapy stress. Furthermore, our *in vivo* experiments demonstrate that silencing CDCA3 enhances the sensitivity of BLCA xenografts to gemcitabine treatment.

Based on these findings, we hypothesize that high expression of CDCA3 and ENO1 may correlate with reduced chemosensitivity in BLCA patients. To address this limitation, we plan to collect clinical samples in future studies and perform correlation analyses to validate this hypothesis.

We greatly appreciate the reviewer’s insightful suggestion, which has inspired us to further investigate this important question. We are confident that future work will benefit from these valuable insights.

Rebuttal figure 8. Clinicopathological characteristics of BLCA patients based on *CDCA3* level from GSE13507 dataset [1].

Rebuttal figure 9. Clinicopathological characteristics of BLCA patients based on *ENO1* level from GSE13507 dataset [21].

*- Figure 2A e 8A. In figure 2A and 8A the regression line is missing. Within the correlation analysis of two factors, it is mandatory to insert the regression line for the correlation between two variables.*

Response: We sincerely thank the reviewer for the kind reminding. GEPIA online database did not present the regression line of two selected genes. We investigated the TIMER 2.0 online database (http://timer.cistrome.org/) which provides the regression line for the correlation between two factors. Figure 2A, Figure 8A and Table 1 were updated according to data acquired from TIMER 2.0 database in the revised manuscript.

*- Page 5 line 320: the sentence "Our molecular docking analysis suggests a possible interaction between CDCA3 and 321 MYC (Figure 5A)" is referring to Figure 6A, not 5A.*

Response: We appreciate the carefully reading of our manuscript by the reviewer and we apologize for the mistake. We have corrected the mistake.

*- Page 13, line 338, sentence "By intersecting these seven proteins with proteins that interact with MYC, previously reported by Dr. Qing": the cited reference must be addressed at the end of the sentence.*

Response: We sincerely thank the reviewer’s careful check of the manuscript. We have added the correct references in the corresponding location.

**Rebuttal reference:**

1.	Shen D, Fang Y, Zhou F, Deng Z, Qian K, Wang G, Xiao Y, Ju L, Wang X **The inhibitory effect of silencing CDCA3 on migration and proliferation in bladder urothelial carcinoma**. Cancer Cell Int 2021, **21**(1):257.

2.	Liu W, Shen D, Ju L, Zhang R, Du W, Jin W *et al* **MYBL2 promotes proliferation and metastasis of bladder cancer through transactivation of CDCA3**. Oncogene 2022, **41**(41):4606-4617.

3.	Adams MN, Burgess JT, He Y, Gately K, Snell C, Zhang SD, Hooper JD, Richard DJ, O'Byrne KJ **Expression of CDCA3 Is a Prognostic Biomarker and Potential Therapeutic Target in Non-Small Cell Lung Cancer**. J Thorac Oncol 2017, **12**(7):1071-1084.

4.	Qian W, Zhang Z, Peng W, Li J, Gu Q, Ji D *et al* **CDCA3 mediates p21-dependent proliferation by regulating E2F1 expression in colorectal cancer**. Int J Oncol 2018, **53**(5):2021-2033.

5.	Zou RC, Guo ZT, Wei D, Shi ZT, Ye ZC, Zhai G *et al* **Downregulation of CDCA3 expression inhibits tumor formation in pancreatic cancer**. Neoplasma 2020, **67**(6):1223-1232.

6.	Hartmann N, Giese NA, Giese T, Poschke I, Offringa R, Werner J, Ryschich E **Prevailing role of contact guidance in intrastromal T-cell trapping in human pancreatic cancer**. Clin Cancer Res 2014, **20**(13):3422-3433.

7.	Jiang Y, Chen X, Tian W, Yin X, Wang J, Yang H **The role of TGF-beta1-miR-21-ROS pathway in bystander responses induced by irradiated non-small-cell lung cancer cells**. Br J Cancer 2014, **111**(4):772-780.

8.	Heinicke U, Fulda S **Chemosensitization of rhabdomyosarcoma cells by the histone deacetylase inhibitor SAHA**. Cancer Lett 2014, **351**(1):50-58.

9.	Munagala R, Aqil F, Jeyabalan J, Gupta RC **Tanshinone IIA inhibits viral oncogene expression leading to apoptosis and inhibition of cervical cancer**. Cancer Lett 2015, **356**(2 Pt B):536-546.

10.	Zhang J, Ramadan AM, Griesenauer B, Li W, Turner MJ, Liu C *et al* **ST2 blockade reduces sST2-producing T cells while maintaining protective mST2-expressing T cells during graft-versus-host disease**. Sci Transl Med 2015, **7**(308):308ra160.

11.	Wu S, Cao R, Tao B, Wu P, Peng C, Gao H, Liang J, Yang W **Pyruvate Facilitates FACT-Mediated gammaH2AX Loading to Chromatin and Promotes the Radiation Resistance of Glioblastoma**. Adv Sci (Weinh) 2022, **9**(8):e2104055.

12.	Xue Y, Wang SK, Rana P, West ER, Hong CM, Feng H, Wu DM, Cepko CL **AAV-Txnip prolongs cone survival and vision in mouse models of retinitis pigmentosa**. Elife 2021, **10**.

13.	Park MK, Zhang L, Min KW, Cho JH, Yeh CC, Moon H *et al* **NEAT1 is essential for metabolic changes that promote breast cancer growth and metastasis**. Cell Metab 2021, **33**(12):2380-2397 e2389.

14.	Vantaku V, Dong J, Ambati CR, Perera D, Donepudi SR, Amara CS *et al* **Multi-omics Integration Analysis Robustly Predicts High-Grade Patient Survival and Identifies CPT1B Effect on Fatty Acid Metabolism in Bladder Cancer**. Clin Cancer Res 2019, **25**(12):3689-3701.

15.	Cheng S, Wang G, Wang Y, Cai L, Qian K, Ju L, Liu X, Xiao Y, Wang X **Fatty acid oxidation inhibitor etomoxir suppresses tumor progression and induces cell cycle arrest via PPARgamma-mediated pathway in bladder cancer**. Clin Sci (Lond) 2019, **133**(15):1745-1758.

16.	Zeng Y, Luo Y, Zhao K, Liu S, Wu K, Wu Y *et al* **m6A-Mediated Induction of 7-Dehydrocholesterol Reductase Stimulates Cholesterol Synthesis and cAMP Signaling to Promote Bladder Cancer Metastasis**. Cancer Res 2024, **84**(20):3402-3418.

17.	Wei X, Feng J, Chen L, Zhang C, Liu Y, Zhang Y *et al* **METTL3-mediated m6A modification of LINC00520 confers glycolysis and chemoresistance in osteosarcoma via suppressing ubiquitination of ENO1**. Cancer Lett 2024:217194.

18.	Ma L, Xue X, Zhang X, Yu K, Xu X, Tian X *et al* **The essential roles of m(6)A RNA modification to stimulate ENO1-dependent glycolysis and tumorigenesis in lung adenocarcinoma**. J Exp Clin Cancer Res 2022, **41**(1):36.

19.	Chen B, Deng Y, Hong Y, Fan L, Zhai X, Hu H *et al* **Metabolic Recoding of NSUN2-Mediated m(5)C Modification Promotes the Progression of Colorectal Cancer via the NSUN2/YBX1/m(5)C-ENO1 Positive Feedback Loop**. Adv Sci (Weinh) 2024, **11**(28):e2309840.

20.	Xu X, Chen Y, Shao S, Wang J, Shan J, Wang Y *et al* **USP21 deubiquitinates and stabilizes HSP90 and ENO1 to promote aerobic glycolysis and proliferation in cholangiocarcinoma**. Int J Biol Sci 2024, **20**(4):1492-1508.

21.	Shen D, Deng Z, Liu W, Zhou F, Fang Y, Shan D *et al* **Melatonin inhibits bladder tumorigenesis by suppressing PPARgamma/ENO1-mediated glycolysis**. Cell Death Dis 2023, **14**(4):246.

22.	Pornour M, Jeon HY, Ryu H, Khadka S, Xu R, Chen H *et al* **USP11 promotes prostate cancer progression by up-regulating AR and c-Myc activity**. Proc Natl Acad Sci U S A 2024, **121**(31):e2403331121.

23.	Wang K, Luo L, Fu S, Wang M, Wang Z, Dong L *et al* **PHGDH arginine methylation by PRMT1 promotes serine synthesis and represents a therapeutic vulnerability in hepatocellular carcinoma**. Nat Commun 2023, **14**(1):1011.

24.	Xie M, Zhou L, Li T, Lin Y, Zhang R, Zheng X *et al* **Targeting the KAT8/YEATS4 Axis Represses Tumor Growth and Increases Cisplatin Sensitivity in Bladder Cancer**. Adv Sci (Weinh) 2024, **11**(22):e2310146.

25.	Luo L, Wu X, Fan J, Dong L, Wang M, Zeng Y *et al* **FBXO7 ubiquitinates PRMT1 to suppress serine synthesis and tumor growth in hepatocellular carcinoma**. Nat Commun 2024, **15**(1):4790.
